# The Effects of Dose, Practice Habits, and Objects of Focus on Digital Meditation Effectiveness and Adherence: Longitudinal Study of 280,000 Digital Meditation Sessions Across 103 Countries

**DOI:** 10.2196/43358

**Published:** 2023-09-19

**Authors:** Micah Cearns, Scott R Clark

**Affiliations:** 1 Insight Timer Research Insight Timer Sydney Australia; 2 Discipline of Psychiatry The University of Adelaide Adelaide Australia

**Keywords:** mindfulness, meditation, digital meditation, mindfulness dose-response, meditation dose-response, dose-response, meditation adherence, mindfulness adherence, longitudinal meditation research, outcome, ecological memory assessment, mental well-being, healthy lifestyle, digital health intervention

## Abstract

**Background:**

The efficacy of digital meditation is well established. However, the extent to which the benefits remain after 12 weeks in real-world settings remains unknown. Additionally, findings related to dosage and practice habits have been mixed, and the studies were conducted on small and homogeneous samples and used a limited range of analytical procedures and meditation techniques. Findings related to the predictors of adherence are also lacking and may help inform future meditators and meditation programs on how to best structure healthy sustainable practices.

**Objective:**

This study aimed to measure outcome change across a large and globally diverse population of meditators and meditations in their naturalistic practice environments, assess the dose-response relationships between practice habits and outcome change, and identify predictors of adherence.

**Methods:**

We used ecological momentary assessment to assess participants’ well-being over a 14-month period. We engineered outcomes related to the variability of change over time (equanimity) and recovery following a drop in mood (resilience) and established the convergent and divergent validity of these outcomes using a validated scale. Using linear mixed-effects and generalized additive mixed-effects models, we modeled outcome changes and patterns of dose-response across outcomes. We then used logistic regression to study the practice habits of participants in their first 30 sessions to derive odds ratios of long-term adherence.

**Results:**

Significant improvements were observed in all outcomes (*P*<.001). Generalized additive mixed models revealed rapid improvements over the first 50-100 sessions, with further improvements observed until the end of the study period. Outcome change corresponded to 1 extra day of improved mood for every 5 days meditated and half-a-day-faster mood recovery compared with baseline. Overall, consistency of practice was associated with the largest outcome change (4-7 d/wk). No significant differences were observed across session lengths in linear models (mood: *P*=.19; equanimity: *P*=.10; resilience: *P*=.29); however, generalized additive models revealed significant differences over time (*P*<.001). Longer sessions (21-30 min) were associated with the largest magnitude of change in mood from the 20th session onward and fewer sessions to recovery (increased resilience); midlength sessions (11-20 min) were associated with the largest decreases in recovery; and mood stability was similar across session lengths (equanimity). Completing a greater variety of practice types was associated with significantly greater improvements across all outcomes. Adhering to a long-term practice was best predicted by practice consistency (4-7 d/wk), a morning routine, and maintaining an equal balance between interoceptive and exteroceptive meditations.

**Conclusions:**

Long-term real-world digital meditation practice is effective and associated with improvements in mood, equanimity, and resilience. Practice consistency and variety rather than length best predict improvement. Long-term sustainable practices are best predicted by consistency, a morning routine, and a practice balanced across objects of focus that are internal and external to the body.

## Introduction

### Background

The efficacy of mindfulness meditation through digital and nondigital media is well established [1-4]. However, the extent to which real-world benefits remain after the first 8 to 12 weeks remains unknown [1]. It is also unclear how *much* meditation at *what* level of consistency (dosage and practice habits) is most effective for *whom* [5], whether this changes over the course of a meditator’s journey as they gain experience, and how these factors interact with the types of meditation practiced [6].

In the context of rising rates of mental illness, stress, burnout, and absenteeism worldwide, digital meditation presents itself as a promising supplement to assist in the management of these problems and improve overall well-being [1,7]. However, although digital meditation appears efficacious in structured, controlled, and prescribed environments [7], there are no large-scale studies that have assessed tolerance, adherence, and effectiveness in the *real world*. Therefore, a detailed real-world analysis of the effects of dose and practice habits across a diverse range of meditators and meditation types is required to understand whether digital meditation confers long-term benefits.

Thus far, the findings on the effects of dose and practice habits in controlled environments have been mixed. A meta-analysis found that brief mindfulness-based programs were as effective as higher-dose mindfulness-based programs for psychological distress in working nonclinical populations [8]. In clinical populations, a review of between-session home practice frequency and outcome change identified a dose-response relationship in only half of the reviewed studies [9]. A meta-regression by Strohmaier [5] found dose-response relationships for mindfulness compared with controls associated with program intensity, use, and teacher-trainee face-to-face contact. However, no relationships were found between session length and psychological outcomes after controlling for baselines and making false discovery rate corrections [5]. In a 2-week–long randomized controlled trial (RCT), Strohmaier et al [10] found an effect of mindfulness meditation on trait mindfulness, depression, anxiety, and stress in beginner meditators. Interestingly, evidence favored shorter session lengths for trait mindfulness and anxiety [10].

Even though the effects of dosage are unclear, specific components of meditation also contribute to its effectiveness. For example, different types of meditation contain distinct cognitive and therapeutic mechanisms [11,12] and direct the meditator to different objects of focus [13]. For example, mindfulness of the breath, body scans, and many secular practices place the object of focus internally in the body (interoception), whereas alternative and spiritually and religiously oriented meditations often place the object of focus externally (exteroception), such as on compassionate thoughts for others, a positive self-affirmation, or a mantra [14]. Britton [14] proposed that these different objects of focus may have implications for long-term efficacy and adherence. For example, body-focused interoceptive meditations encourage growth in the insular cortex, a region thought to be involved in bodily perception and emotion. Although interoceptive meditations are more common in digital meditation (and the main focus of research in controlled study environments), a disproportionate practice of these meditations might risk oversensitizing a meditator to bodily and emotional states [11,14]. On the contrary, balancing these practices with those that place the object of focus outside the body may mitigate this risk of oversensitization and help a meditator break patterns of internal rumination [14].

In the same way that meditations vary in their attributes, so do the individual and cultural characteristics of meditators [15,16]. Exploring the use of digital meditation across a large and culturally diverse sample is required to understand the relationship between a meditation practice and outcome change. Indeed, the inconsistency of findings related to dose and practice may be attributable to the use of small and nonrepresentative samples. Furthermore, nonlinear dose-response relationships may have been overlooked in previous studies that used linear techniques, whereas the use of infrequently measured outcomes may have masked the relationship between dosage and outcomes [17]. Large heterogeneous samples, nonlinear modeling techniques, and ecological momentary assessment (EMA; a method that frequently captures how an individual feels over time) [18] can be used to overcome these limitations.

Recently, the use of EMA in mobile meditation studies has increased and demonstrated positive changes in well-being, depression, emotion regulation, and mindfulness [17,19-21] and has shown an increased sensitivity to detect change compared with traditional assessment techniques [17]. A single-item mood (SIM) scale is a common form of EMA that asks a user how they feel and measures their responses on a Likert scale. Given its open-ended nature, it can also be used to create secondary measures. For example, equanimity, a state of psychological stability and composure [22], can be engineered by measuring a meditator’s SIM variability, whereas resilience, a psychological state improved by mindfulness [23] and predictor of well-being [24,25], can be measured by looking at the time it takes for a meditator’s SIM to recover following a decrease from its norm. In addition to studying EMA-based outcomes, understanding adherence and its predictors may help inform future meditators and meditation programs on how to best structure healthy, sustainable practices. Previous research has shown that personality characteristics, depressive symptoms, motivation, trait mindfulness, and the use of EMA itself are positively associated with adherence [24-26]. However, the relationship between a meditator’s early practice habits and adherence remains unknown.

### Objectives

Given the increasing global burden of disability arising from mental health disorders [27], the general efficacy of mindfulness meditation in clinical and nonclinical populations [28], and the ease with which meditative practices can be completed in solitary or group settings, using EMA to answer questions related to practice habits, dosage, effectiveness, and adherence may help personalize mindfulness meditation for meditators [29]. Therefore, we examined the longitudinal association between the total number of meditation sessions completed both overall and per week, session length, the length of time between sessions, ratios of interoceptive and exteroceptive practices, changes in meditator mood, equanimity, resilience, and the odds of long-term practice adherence using the mobile meditation app Insight Timer. The goal was to measure patterns of change over time and determine the optimal dose, frequency, and duration across a diverse set of meditators and meditations while accounting statistically for user characteristics and covariates that might confound these relationships.

## Methods

### Recruitment

New English-speaking Insight Timer users who were aged ≥18 years, completed onboarding (registered for an account and provided information on their characteristics and previous meditation experience), and used Insight Timer’s mood check-in feature were sent an in-app message containing a plain-language statement and informed consent form. Only those who consented to having their in-app use data used for peer-reviewed research purposes were included in the analysis. Insight Timer also collects routine survey data from users for product review, safety, quality assurance, and research purposes. After providing informed consent for deidentified app use analysis, users were linked to an additional survey in the in-app message and completed the even-minded state of mind subscale of the Two-Factor Equanimity Scale. These users were provided with a second plain-language statement and consent form in which they consented to the use of their survey data for quality audit and peer-reviewed research purposes as well as the linking of their survey responses to their app use data. The in-app message containing the plain-language statement, informed consent statement, and user survey was made available to users from November 24, 2021, to December 22, 2021.

### Outcome Variables and Data Structure

#### SIM Scale

Users’ mood was measured in each meditation session using a 5-level single-item Likert SIM scale (a form of EMA), an alternative to traditional clinical assessment that affords repeated measurements in real-life settings, leading to increased reliability and ecological validity [18]. Users were presented with a screen asking the following—“How are you today?”—and responded by selecting 1 of 5 icons representing *terrible*, *bad*, *okay*, *good*, or *great*. See Figure S1 in Multimedia Appendix 1 [30-34] for a visualization and the supplementary methods section in Multimedia Appendix 1 for more information. In addition, see Figure S2 in Multimedia Appendix 1 for a visualization of Insight Timer’s new user onboarding flow at the time of data collection.

#### Shifted Mood Scores and Practice Periods

As users reported their mood before each meditation, we shifted each mood score forward so that each session predicted the following session’s score. For example, meditation session 1 predicted the score for meditation session 2 and so forth. This also allowed for the use of the first score as a baseline covariate. See Figure S3 in Multimedia Appendix 1 for an example of this structure. Although this structure increased our confidence in causative relationships, this became invalidated when there was a large gap between sessions. For instance, if a user did not meditate for 2 weeks, it is implausible that the meditation session from 14 days before would have a predictive effect on the meditator’s current mood. Instead, whatever confounds caused this break would likely be responsible (eg, increased work stress and time spent on other well-being activities). To circumvent this problem, we developed the notion of practice periods.

We defined a practice period as any consecutive period of meditation with no longer than 7 days between sessions. We chose this value as it captured 90% of the distribution for days between sessions and naturally fit within a weekly cycle of everyday habits (Figure S4 in Multimedia Appendix 1). For example, if a user completed 20 meditations, and across them, there was a range of breaks between 1 and 7 days, all 20 of these meditations would be in 1 practice period. If the user then did not meditate for >7 days and then completed another 20 sessions, all completed within ≤7 days, these subsequent sessions would be considered as meditations completed in a second practice period.

Therefore, when we shifted users’ mood scores, we shifted them for each user within their practice period. Thus, each time a user did not meditate for >7 days and returned to meditate, we removed and controlled for their first mood score as a baseline covariate in that practice period and then brought forward every other mood score for prediction. This way, we could control for confounding variables in between sessions without the need to know precisely what they were while mitigating the risk of misattributing outcome change to meditation sessions when significant gaps were present between them. See Figure S3 in Multimedia Appendix 1 for more information.

#### Equanimity

Many authors suggest using equanimity, a psychologically relevant mental state derived from Buddhist literature, as an outcome in contemplative research [22,35]. This state can be defined as [22] “An even-minded state of mind or dispositional tendency toward all experiences or objects, regardless of their affective valence (pleasant, unpleasant, or neutral) or source” [22]. According to this definition, equanimity is associated with less emotional interference [36], greater emotional stability and its underlying neural correlates [37], higher levels of inner peace [38], and reduced general stress [39].

As this state comprises composure and stability, we should expect that, when a meditator’s equanimity increases, their SD of mood should decrease. Therefore, we measured equanimity based on the rolling 5-session SD of the user’s mood (5 sessions represented the median number of sessions within a practice period per user; Figure S5 in Multimedia Appendix 1). We turned this into a standardized measure by sign flipping these values (decreasing SD was now measured by increasing, not decreasing, values) and normalized each value on a range from 1 to 5. This was done to have an equanimity measure on the same scale as the primary mood outcome and to intuitively observe increasing equanimity with positive values and decreasing equanimity with negative values. We established this measure’s convergent and divergent validity using each item on the even-minded state of mind subscale from the Two-Factor Equanimity Scale [30].

#### Resilience: Number of Sessions to Recovery Following a 1-Point Mood Drop

To measure resilience, we created a variable that measured the number of sessions to recovery following a 1-point drop in mood from the meditator’s previous week’s average (see Figure S6 in Multimedia Appendix 1 for the distribution of SIM measurements per week). Specifically, when a meditator’s mood dropped 1 point below their previous week average, a counter started counting until the user reverted to or above their previous week’s mood. With this outcome, we would expect more resilient users to revert sooner. If more meditation sessions increase a user’s resilience over time, we would expect more sessions to be associated with faster reversion. To avoid confounding this measure by users who churned (ceased to continue meditating), we excluded users who had a 1-point mood drop but had not yet returned for another session. We also established this measure’s convergent and divergent validity using each item on the even-minded state of mind subscale of the Two-Factor Equanimity Scale [30] (Multimedia Appendix 1).

#### Adherence

To measure adherence, we looked at meditators’ practice habits in their first 30 sessions. We chose this value as it corresponded to the median number of sessions completed. We then defined adhering users as those who reached the top decile (10%) of the session distribution as this corresponded to completing a high number of sessions (≥150 meditation sessions) while retaining an adequate sample size (365/2084, 17.51% of meditators who completed their first 30 sessions; Figure S7 in Multimedia Appendix 1).

### Predictor Variables and Measures

As we were interested in using guided meditations performed by the respondents and related to their SIM, we removed all meditations related to children as it was possible that it was not the parent completing the meditation. We also removed sleep meditations as we could not verify at what point a user may have fallen asleep relative to the length of a track, which would have confounded any dose-response relationships. As users were free to complete mood check-ins as desired and all in-app use was logged, all remaining data were used in the analyses.

To calculate participants’ total number of meditation sessions, we cumulatively summed the number of sessions completed by each participant. We then harmonized this variable into days per week and days since the last meditation variables using each session’s time stamp and then harmonized the length of each session from seconds to minutes. To measure the effects of performing different proportions of meditations with an object of focus external to the body (eg, compassion and loving-kindness meditation, henceforth referred to as *exteroceptive meditations*) compared with an object of focus internal to the body (eg, mindfulness of the breath and body scans, henceforth referred to as *interoceptive meditations*), we divided the cumulative number of exteroceptive meditations by the cumulative number of interoceptive meditations over time per user (Figure S8 in Multimedia Appendix 1). To measure the effects of performing multiple practice types, we summed the number of distinct meditation types performed per meditator as they completed their sessions. Henceforth, these variables are referred to as our predictor variables.

To reduce the possibility of spurious outliers, we applied trimming using the box plot method to the total number of meditation sessions completed and each meditation session length such that responses greater than 291 meditation sessions and 30 minutes were removed from the analyses. Total meditation sessions and session lengths were harmonized to the nearest 5 minutes and 5 meditation sessions (Figures S9 and S10 in Multimedia Appendix 1) to reduce sparsity and minimize estimation errors.

In subsequent analyses, we used these variables to predict our mood, equanimity, and resilience outcomes. In all analyses, we controlled for the following onboarding covariates: why a meditator was meditating (managing stress, anxiety, or sadness or improving their well-being), content preferences, previous meditation experience, and age. From the meditators’ mood check-ins, we controlled for their baseline mood for each practice period and their mood attributions. We also controlled for content-related covariates that could confound the relationship between dosage and outcome change. These included the ratings and number of ratings, play counts, meditation topic sentiment (eg, positivity focused, problem focused, or techniques), worldview of the meditation (eg, scientific or secular, Buddhist, religious, or of other spiritual origins), and practice type of each meditation (eg, Vipassana, loving-kindness, guided imagery, and mindfulness of the breath; see the supplementary methods 3 section in Multimedia Appendix 1 for more information). Henceforth, these variables are referred to as the full covariate set.

### Statistical Analysis

Outcome data were hierarchical in nature (users came from 103 countries) and contained repeated measures (multiple guided meditation sessions and outcome measurements per user). For linear models of outcome change, we used hierarchical mixed-effects models with longitudinal mood, equanimity, and resilience scores as dependent variables. We used random effects to nest each participant in their continent of use while fitting random intercepts for each participant. Next, we used fixed effects to model our predictor variables while controlling for the full covariate set.

We then repeated these analyses using generalized additive mixed models to observe nonlinear relationships for each outcome. The model used penalized thin-plate regression splines with parametric regressors to control for each covariate. We plotted the fitted smoothed coefficients with 95% CIs for the number of completed meditation sessions by the number of days meditated per week, days since the last meditation session, length of each session (our practice habit variables), ratio of exteroceptive to interoceptive sessions, and number of distinct practice types completed. To ensure adequate sample sizes at higher session time points and practice habits, we calculated the relative frequencies of each time point for each level of our practice habit variables and used them to derive session time point cutoffs for our generalized additive mixed models (see the supplementary methods 4 section, Tables S1-S3, and Figure S9 in Multimedia Appendix 1).

Finally, we used logistic regression to model adherence, regressing our predictor variables and full covariate set from the first 30 meditation sessions onto a binary outcome representing whether a meditator reached their 150th session.

### Ethics Approval

This study was approved by the University of Adelaide ethics committee (approval H-2023-111).

### Informed Consent and Resources

All participants provided informed consent. Mental health and crisis resources were provided to all participants via the in-app message, the survey, and Insight Timer’s website and mobile app [40].

## Results

### Sample Characteristics

Overall, 10,409 participants from 103 countries took part in the study between May 18, 2021, and August 14, 2022. During this time, they completed 289,630 meditation sessions with associated mood check-ins for a total of 3,331,079 minutes meditated across 1392 unique practice type combinations. Users had an average age of 37.5 (SD 12.6) years and checked in and meditated an average of 44.2 (SD 61.9; median 19, IQR 6-55) times at an average of 1.5 (SD 0.5; median 1.3, IQR 1.74-3.69) sessions per week with an average of 1 (SD 0.8; median 1.2, IQR 0.3-1.6) day since their last meditation. A mean of 5.3 (SD 3.8; median 4, IQR 2-8) practice periods was completed, with an average of 21.8 (SD 23.5; median 16, IQR 6.9-28.9) days between them.

In total, 68.41% (7121/10,409) of users were using Insight Timer to manage anxiety, 65.28% (6795/10,409) were using it to manage stress, 36.69% (3819/10,409) were using it to manage sadness, and 63.17% (6575/10,409) were using it to improve their well-being (multiple selections were permitted to users during onboarding). Of these 10,409 participants, 2033 (19.53%) had no experience of meditation, 2457 (23.6%) had experience with web-based meditation courses, 7282 (69.96%) had experience with meditation apps, 2911 (27.97%) had experience with local meditation classes, 1490 (14.31%) had experience with mentoring, and 1455 (13.98%) had experience at meditation retreats. See Tables 1 and 2 for sample characteristics, the supplementary results in Multimedia Appendix 1 for additional participant characteristics and outcome intercorrelations, Table S4 in Multimedia Appendix 1 for participant counts and mood scores by country, and Tables S5 and S6 in Multimedia Appendix 1 for the even-minded state of mind subscale of the Two-Factor Equanimity Scale subsample characteristics.

**Table 1:** Continuous variable descriptive statistics for the mood sample, the equanimity subsample, and the resilience subsample (N=10,409).

	Full mood score sample			Equanimity score sample			Resilience score sample	
	Participants, n (%)	Mean (SD)	Participants, n (%)		Mean (SD)	Participants, n (%)		Mean (SD)
Age (years)	10,409 (100)	37.45 (12.62)	5475 (52.59)		37.65 (12.64)	1972 (18.94)		38.41 (12.53)
Baseline mood score	10,409 (100)	3.34 (0.72)	5475 (52.59)		3.4 (0.8)	1972 (18.94)		3.46 (0.66)
Number of practice periods	10,409 (100)	5.26 (3.84)	5475 (52.59)		4.39 (3.51)	1972 (18.94)		7.82 (3.97)
Days between practice periods	10,409 (100)	21.82 (23.5)	5475 (52.59)		15.09 (24.83)	1972 (18.94)		14.57 (14.17)
Number of mood check-ins	10,409 (100)	3.4 (0.64)	5475 (52.59)		3.53 (0.64)	1972 (18.94)		3.56 (0.51)
Equanimity score	5475 (52.59)	4.04 (0.48)	5475 (52.59)		4.04 (0.48)	1967 (18.89)		4.01 (0.38)
Resilience score	1972 (18.94)	1.55 (0.31)	1967 (18.89)		1.55 (0.31)	1972 (18.94)		1.55 (0.31)
Number of meditation sessions	10,409 (100)	44.24 (61.85)	5475 (52.59)		62.97 (69.72)	1972 (18.94)		125.45 (77.41)
Session length (min)	10,409 (100)	11.45 (4.11)	5475 (52.59)		11.53 (4.29)	1972 (18.94)		11.68 (3.73)
Days per week	10,409 (100)	1.48 (0.51)	5475 (52.59)		2.13 (0.65)	1972 (18.94)		2.08 (0.49)
Days since last meditation	10,409 (100)	1.07 (0.76)	5475 (52.59)		2.05 (1.06)	1972 (18.94)		1.62 (0.37)
Rating score	10,409 (100)	4.72 (0.12)	5475 (52.59)		4.72 (0.13)	1972 (18.94)		4.74 (0.1)
Rating count	10,409 (100)	180,000 (190,000)	5475 (52.59)		170,000 (210,000)	1972 (18.94)		150,000 (160,000)
Play count	10,409 (100)	4,300,000 (5,100,000)	5475 (52.59)		4,100,000 (5,800,000)	1972 (18.94)		3,900,000 (4,600,000)
Number of practice types	10,409 (100)	4.73 (2.98)	5475 (52.59)		5.95 (2.9)	1972 (18.94)		7.68 (3.0)
Exteroceptive to interoceptive session ratio	8636 (82.96)	4.17 (11.19)	4862 (46.70)		5.59 (13.78)	1837 (17.64)		8.82 (20.8)

**Table 2:** Categorical variable descriptive statistics. As statistics are by user, not session, modes are reported for session-related characteristics (practice type, worldview, and orientation).

			Full mood score sample					Equanimity score sample					Resilience score sample		
			Participants, (n=10,409), n (%)		Mood, mean (SD)		Participants, (n=5475) n (%)			Mood, mean (SD)		Participants, (n=1972) n (%)			Mood, mean (SD)
**Continent**															
	Africa	155 (1.49)		3.52 (0.58)		94 (1.72)			3.56 (0.54)		43 (2.18)			3.51 (0.47)	
	Asia	396 (3.8)		3.46 (0.78)		184 (3.36)			3.52 (0.64)		58 (2.94)			3.57 (0.56)	
	Australia and Oceania	982 (9.43)		3.38 (0.65)		528 (9.64)			3.46 (0.53)		172 (8.72)			3.58 (0.51)	
	Europe	2279 (21.89)		3.33 (0.64)		1270 (23.2)			3.41 (0.55)		473 (23.99)			3.48 (0.53)	
	North America	6256 (60.1)		3.42 (0.63)		3193 (58.32)			3.52 (0.53)		1141 (57.86)			3.59 (0.5)	
	South America	341 (3.28)		3.57 (0.63)		206 (3.76)			3.63 (0.62)		85 (4.31)			3.67 (0.55)	
**Experience**															
	Local class (yes)	2911 (27.97)		3.43 (0.6)		1557 (28.44)			3.53 (0.52)		585 (29.67)			3.58 (0.5)	
	Meditation apps (yes)	7282 (69.96)		3.43 (0.63)		3968 (72.47)			3.51 (0.54)		1500 (76.06)			3.56 (0.52)	
	Mentoring (yes)	1490 (14.31)		3.5 (0.62)		847 (15.47)			3.58 (0.54)		324 (16.43)			3.63 (0.51)	
	No experience (yes)	2033 (19.53)		3.28 (0.68)		950 (17.35)			3.4 (0.57)		275 (13.95)			3.53 (0.5)	
	Web-based course (yes)	2457 (23.6)		3.47 (0.63)		1358 (24.8)			3.56 (0.54)		518 (26.27)			3.61 (0.5)	
	Retreats (yes)	1455 (13.98)		3.5 (0.61)		790 (14.43)			3.56 (0.53)		293 (14.86)			3.62 (0.48)	
**Practice type**															
	Alternative	5455 (52.41)		3.46 (0.6)		3005 (54.89)			3.55 (0.55)		1294 (65.62)			3.61 (0.5)	
	Body scan	403 (3.87)		3.22 (0.68)		222 (4.05)			3.36 (0.49)		75 (3.8)			3.31 (0.45)	
	Breathing meditation	681 (6.54)		3.32 (0.71)		344 (6.28)			3.41 (0.55)		76 (3.85)			3.53 (0.44)	
	Compassion meditation	185 (1.78)		3.24 (0.67)		104 (1.9)			3.28 (0.57)		17 (0.86)			3.16 (0.52)	
	Contemplation	281 (2.7)		3.32 (0.68)		170 (3.11)			3.44 (0.51)		43 (2.18)			3.36 (0.51)	
	Guided imagery	981 (9.42)		3.34 (0.7)		454 (8.29)			3.44 (0.53)		108 (5.48)			3.48 (0.53)	
	Loving-kindness (metta)	79 (0.76)		3.34 (0.67)		39 (0.71)			3.51 (0.65)		6 (0.3)			3.51 (0.46)	
	MBCT^a^ or MBSR^b^	67 (0.64)		3.1 (0.66)		32 (0.58)			3.34 (0.59)		4 (0.2)			3.14 (0.42)	
	Mindfulness meditation	1538 (14.78)		3.32 (0.64)		628 (11.47)			3.42 (0.52)		200 (10.14)			3.47 (0.53)	
	Positive affirmations	479 (4.6)		3.59 (0.6)		358 (6.54)			3.59 (0.53)		123 (6.24)			3.7 (0.51)	
	Relaxation meditation	131 (1.26)		3.21 (0.83)		65 (1.19)			3.28 (0.53)		15 (0.76)			3.3 (0.53)	
	Vipassana	129 (1.24)		3.42 (0.71)		54 (0.99)			3.31 (0.39)		11 (0.56)			3.2 (0.38)	
**Meditation worldview**															
	Hinduism	9 (0.09)		3.46 (0.84)		3 (0.05)			3.33 (0.53)		—^c^			—	
	Buddhism	727 (6.98)		3.34 (0.68)		348 (6.36)			3.45 (0.52)		42 (2.13)			3.59 (0.54)	
	Islam	—		—		—			—		—			—	
	Christianity	87 (0.84)		3.33 (0.63)		57 (1.04)			3.55 (0.57)		15 (0.76)			3.58 (0.38)	
	Judaism	2 (0.02)		3.83 (0.24)		2 (0.04)			3.61 (0.06)		—			—	
	Modernism	2485 (23.87)		3.26 (0.68)		1056 (19.29)			3.34 (0.54)		240 (12.17)			3.36 (0.49)	
	Niches	1184 (11.37)		3.55 (0.7)		724 (13.22)			3.6 (0.57)		204 (10.34)			3.72 (0.53)	
	Other	4968 (47.73)		3.43 (0.61)		2677 (48.89)			3.53 (0.53)		1205 (61.11)			3.57 (0.5)	
	Taoism	5 (0.05)		3.48 (0.86)		4 (0.07)			3.66 (0.83)		1 (0.05)			3.07 (nan)	
**Meditation orientation**															
	Niches	2659 (25.55)		3.5 (0.64)		1583 (28.91)			3.57 (0.56)		566 (28.7)			3.63 (0.52)	
	Positivity based	3135 (30.12)		3.54 (0.57)		1857 (33.92)			3.59 (0.5)		725 (36.76)			3.67 (0.45)	
	Problem focused	3319 (31.89)		3.2 (0.65)		1454 (26.56)			3.31 (0.54)		553 (28.04)			3.36 (0.52)	
	Techniques	1296 (12.45)		3.41 (0.66)		581 (10.61)			3.46 (0.53)		128 (6.49)			3.55 (0.49)	
**Reason for meditating**															
	Anxiety (yes)	7121 (68.41)		3.33 (0.63)		3731 (68.15)			3.43 (0.53)		1345 (68.2)			3.5 (0.51)	
	Sadness (yes)	3819 (36.69)		3.25 (0.65)		1981 (36.18)			3.35 (0.53)		679 (34.43)			3.43 (0.52)	
	Stress (yes)	6795 (65.28)		3.35 (0.64)		3529 (64.46)			3.45 (0.54)		1268 (64.3)			3.51 (0.5)	
	Well-being (yes)	6575 (63.17)		3.41 (0.63)		3500 (63.93)			3.5 (0.54)		1273 (64.55)			3.56 (0.51)	

^a^MBCT: mindfulness-based cognitive therapy.

^b^MBSR: mindfulness-based stress reduction.

^c^No mode value (content characteristic not the most common for any participant).

### Linear Outcome Change

#### Mood

A higher number of meditation sessions was significantly associated with higher mood scores in the following session. For every 5 meditation sessions completed, mood increased by 0.0005 (*P*<.001), whereas for every extra day meditated per week, mood increased by 0.0126 (*P*<.001). A higher baseline mood for each practice period was associated with a higher overall mood within a practice period (β=.1861; *P*<.001), whereas every extra day between practice periods led to a decrease in mood of 0.0006 (*P*<.006). A higher ratio of exteroceptive to interoceptive meditations was associated with significantly higher mood scores in subsequent sessions (β=.0034; *P*=.003), as was a higher variety of practice types (β=.0068; *P*<.001). There were no significant associations between session length, the number of days since the last meditation session, or the number of practice periods completed and mood. See Table 3 for all results.

**Table 3:** Linear model coefficients for mood change over time.

			β (95% CI)		*P* value		*Q* value^a^
Intercept			2.931 (2.816 to 3.046)		<.001		<0.001
Baseline mood			.1861 (.1809 to .1913)		<.001		<0.001
Session number			.0005 (.0005 to .0006)		<.001		<0.001
Session length (min)			.0004 (−.0002 to .0010)		.19		0.266
Days meditated per week			.0126 (.0103 to .0149)		<.001		<0.001
Days since last meditation			−.0011 (−.0029 to .0008)		.26		0.351
Practice period			.0004 (−.0015 to .0022)		.70		0.813
Days between practice periods			−.0006 (−.0007 to −.0004)		<.001		<0.001
Exteroceptive to interoceptive session ratio			.0034 (.0012 to .0056)		.003		0.005
Number of practice types completed			.0068 (.0046 to .0089)		<.001		<0.001
Meditation rating			.0087 (.0052 to .0123)		<.001		<0.001
Number of ratings			−.0125 (−.0219 to −.0031)		.009		0.017
Meditation play count			.0124 (.0028 to .0220)		.01		0.019
Age			−.0431 (−.0547 to −.0316)		<.001		<0.001
**Reason for meditating**							
	Anxiety (yes)	−.1296 (−.1550 to −.1041)		<.001		<0.001	
	Stress (yes)	−.0407 (−.0653 to −.0162)		.001		0.003	
	Sadness (yes)	−.1321 (−.1554 to −.1088)		<.001		<0.001	
	Well-being (yes)	.0313 (.0089 to .0537)		.006		0.012	
**Time of day meditated**							
	Night	—^b^		—		—	
	Morning	.0250 (.0149 to .0350)		<.001		<0.001	
	Day	−.0088 (−.0201 to .0024)		.12		0.185	
**Previous experience types**							
	No experience	−.0571 (−.1013 to −.0129)		.01		0.019	
	Meditation apps	−.0006 (−.0362 to .0350)		.98		0.975	
	Web-based course	.0233 (−.0033 to .0499)		.09		0.132	
	Local class	−.0125 (−.0386 to .0135)		.35		0.465	
	Retreats	.0312 (−.0017 to .0642)		.06		0.101	
	Mentoring	.0411 (.0094 to .0728)		.01		0.019	
**Meditation orientation**							
	Positivity based	—		—		—	
	Niches	−.0019 (−.0172 to .0134)		.81		0.876	
	Problem focused	−.0538 (−.0624 to −.0453)		<.001		<0.001	
	Techniques	−.0140 (−.0241 to −.0040)		.006		0.012	
**Practice type**							
	Breathing meditation	—		—		—	
	Body scan	.0047 (−.0181 to .0276)		.68		0.785	
	Compassion meditation	−.0202 (−.0399 to −.0005)		.04		0.088	
	Contemplation	.0017 (−.0176 to .0210)		.86		0.919	
	Guided imagery	−.0069 (−.0214 to .0075)		.35		0.504	
	Loving-kindness (metta)	.0110 (−.0138 to .0359)		.39		0.542	
	MBCT^c^ or MBSR^d^	−.0061 (−.0350 to .0228)		.68		0.785	
	Mindfulness meditation	.0034 (−.0104 to .0173)		.63		0.785	
	Other	.0041 (−.0090 to .0172)		.54		0.710	
	Positive affirmations	.0114 (−.0041 to .0269)		.15		0.242	
	Relaxation meditation	−.0261 (−.0472 to −.0050)		.02		0.033	
	Vipassana	.0054 (−.0195 to .0302)		.67		0.785	
**Meditation worldview**							
	Hinduism	—		—		—	
	Buddhism	.0024 (−.0789 to .0837)		.95		0.973	
	Christianity	−.0078 (−.0946 to .0790)		.86		0.914	
	Islam	−.1429 (−.4462 to .1605)		.36		0.466	
	Judaism	−.0315 (−.1652 to .1023)		.65		0.783	
	Modernism	−.0040 (−.0849 to .0769)		.92		0.960	
	Niches	.0104 (−.0699 to .0907)		.80		0.876	
	Other	.0106 (−.0701 to .0914)		.80		0.876	
	Taoism	.0777 (−.0355 to .1908)		.18		0.260	
**Mood attributions**							
	Exercise	.0439 (.0331 to .0547)		.001		<0.001	
	Family	.0275 (.0186 to .0364)		<.001		<0.001	
	Finances	−.0279 (−.0412 to −.0146)		<.001		<0.001	
	Food or diet	.0265 (.0139 to .0391)		<.001		<0.001	
	Friends	.0418 (.0313 to .0523)		<.001		<0.001	
	Health	−.0336 (−.0424 to −.0248)		<.001		<0.001	
	Relationships	−.0257 (−.0347 to −.0167)		<.001		<0.001	
	Sleep	.0094 (.0004 to .0184)		.04		0.068	
	Spirituality	.0839 (.0733 to .0945)		<.001		<0.001	
	Study	−.0052 (−.0196 to .0092)		.48		0.616	
	Travel	.0506 (.0337 to .0675)		<.001		<0.001	
	Work	−.0299 (−.0383 to −.0215)		<.001		<0.001	

^a^False discovery rate correction for multiple testing.

^b^Variable reference level.

^c^MBCT: mindfulness-based cognitive therapy.

^d^MBSR: mindfulness-based stress reduction.

#### Equanimity

After controlling for the full covariate set and the rolling 5-session average of mood (β=.0318; *P*<.001), a similar pattern of change was observed for equanimity. In addition, both equanimity and resilience measures showed convergent and divergent validity using the even-minded state of mind subscale of the Two-Factor Equanimity Scale. See the supplementary results section in Multimedia Appendix 1. For every 5 meditation sessions completed, equanimity increased by 0.0006 (*P*<.001), whereas for every extra day meditated per week, equanimity increased by 0.002 (*P*=.02). A higher baseline mood for each practice period was associated with lower equanimity within a practice period (β=−.0318; *P*<.001). In contrast, every extra practice period completed was associated with increasing equanimity (β=.0028; *P*=.003). For every extra day since the last meditation session within a practice period, equanimity decreased by 0.0121 (*P<*.001)*.* A higher ratio of exteroceptive to interoceptive meditations was associated with significantly higher equanimity (β=.0012; *P*<.001), as was a higher variety of practice types (β=.0095; *P<*.001). No significant effects were observed for days between practice periods and session length. See Table 4 for all results.

**Table 4:** Linear model coefficients for equanimity change over time (the rolling 5-session SD of meditators’ mood).

			β (95% CI)		*P* value		*Q* value^a^
Intercept			2.665 (2.565 to 2.766)		<.001		<0.001
Session time point			.0006 (.0006 to .0007)		<.001		<0.001
Rolling 5-session mood mean			.3802 (.3740 to .3865)		<.001		<0.001
Baseline mood			−.0318 (−.0371 to −.0265)		<.001		<0.001
Session length (min)			−.0005 (−.0010 to .0001)		.10		0.210
Days per week			.0020 (.0003 to .0038)		.02		0.064
Days since last meditation			−.0121 (−.0139 to −.0104)		<.001		<0.001
Practice period			.0028 (.0009 to .0046)		.003		0.012
Days between practice periods			.0001 (−.0002 to .0003)		.51		0.642
Exteroceptive to interoceptive session ratio			.0012 (.0009 to .0014)		<.001		<0.001
Number of practice types completed			.0095 (.0074 to .0117)		<.001		<0.001
Rating score			−.0009 (−.0039 to .0022)		.58		0.710
Rating count			−.0146 (−.0243 to −.0048)		.003		0.013
Play count			.0141 (.0041 to .0242)		.006		0.019
Age			.0833 (.0708 to .0958)		<.001		<0.001
**Reason for meditating**							
	Anxiety (yes)	.0211 (−.0065 to .0488)		.13		0.240	
	Stress (yes)	.0179 (−.0087 to .0444)		.19		0.291	
	Sadness (yes)	−.0021 (−.0276 to .0233)		.87		0.957	
	Well-being (yes)	−.0009 (−.0252 to .0235)		.94		0.959	
**Time of day meditated**							
	Night	—^b^		—		—	
	Morning	.0367 (.0278 to .0455)		<.001		<0.001	
	Day	−.0026 (−.0127 to .0075)		.62		0.722	
**Previous experience types**							
	No experience	.0326 (−.0161 to .0813)		.19		0.291	
	Meditation apps	−.0064 (−.0453 to .0325)		.75		0.840	
	Local class	−.0243 (−.0525 to .0039)		.09		0.198	
	Web-based course	−.0185 (−.0472 to .0102)		.21		0.302	
	Retreats	−.0094 (−.0450 to .0261)		.60		0.722	
	Mentoring	−.0177 (−.0515 to .0161)		.31		0.414	
**Meditation orientation**							
	Positivity based	—		—		—	
	Niches	.0002 (−.0134 to .0138)		.98		0.977	
	Problem focused	−.0157 (−.0235 to −.0080)		<.001		<0.001	
	Techniques	.0070 (−.0022 to .0162)		.13		0.240	
**Practice types**							
	Breathing meditation	—		—		—	
	Body scan	.0173 (−.0035 to .0381)		.10		0.210	
	Compassion meditation	.0203 (.0029 to .0376)		.02		0.063	
	Contemplation	.0008 (−.0160 to .0175)		.93		0.958	
	Guided imagery	.0185 (.0058 to .0313)		.004		0.016	
	Loving-kindness (metta)	.0086 (−.0132 to .0305)		.44		0.564	
	MBCT^c^ or MBSR^d^	.0169 (−.0091 to .0429)		.20		0.302	
	Mindfulness meditation	.0092 (−.0032 to .0216)		.15		0.258	
	Positive affirmations	.0070 (−.0065 to .0205)		.31		0.414	
	Relaxation meditation	−.0035 (−.0222 to .0152)		.71		0.817	
	Vipassana	.0323 (.0095 to .0551)		.006		0.019	
	Other	.0109 (−.0007 to .0225)		.07		0.152	
**Meditation worldview**							
	Hinduism	—		—		—	
	Buddhism	.0414 (−.0287 to .1116)		.25		0.354	
	Christianity	.0857 (.0110 to .1604)		.03		0.064	
	Islam	−.1201 (−.4030 to .1628)		.41		0.532	
	Judaism	.0822 (−.0314 to .1958)		.16		0.261	
	Modernism	.0534 (−.0162 to .1230)		.13		0.240	
	Niches	.0498 (−.0193 to .1190)		.16		0.261	
	Other	.0548 (−.0146 to .1242)		.12		0.240	
	Taoism	.0891 (−.0096 to .1877)		.08		0.173	
**Mood attributions**							
	Exercise	.0024 (−.0069 to .0117)		.62		0.722	
	Family	−.0081 (−.0158 to −.0004)		.04		0.097	
	Finances	.0006 (−.0110 to .0122)		.92		0.958	
	Food	.0172 (.0064 to .0281)		.002		0.008	
	Friends	.0006 (−.0084 to .0096)		.90		0.957	
	Health	−.0179 (−.0255 to −.0103)		<.001		<0.001	
	Relationships	−.0248 (−.0326 to −.0170)		<.001		<0.001	
	Sleep	−.0054 (−.0131 to .0023)		.17		0.276	
	Spirituality	.0006 (−.0085 to .0097)		.89		0.957	
	Studies	.0164 (.0038 to .0290)		.01		0.033	
	Travel	−.0150 (−.0292 to −.0007)		.04		0.097	
	Work	.0129 (.0056 to .0201)		<.001		0.003	

^a^False discovery rate correction for multiple testing.

^b^Variable reference level.

^c^MBCT: mindfulness-based cognitive therapy.

^d^MBSR: mindfulness-based stress reduction.

#### Resilience

A higher number of completed meditations was significantly associated with a lower number of sessions to recovery following a 1-point drop in mood. For every 5 meditation sessions completed, the number of sessions to recovery decreased by −0.0016 (*P*<.001), whereas for every extra day meditated per week, the number of sessions to recovery decreased by −0.0254 (*P*=.003). For every extra day since the last meditation session within a practice period, the number of sessions to recovery decreased by −0.0652 (*P<*.001), whereas for every extra practice period completed, the number of sessions to recovery decreased by −0.0081 (*P*<.001). After controlling for the number of completed sessions alongside the full covariate set, a higher number of completed practice types was associated with a lower number of sessions to recovery (β=−.0125; *P*<.001). No significant effects were observed for higher baseline mood, days between practice periods, exteroceptive to interoceptive session ratios, or session length. See Table 5 for all results.

**Table 5:** Linear model coefficients for changes in resilience over time (the number of sessions to recovery following a 1-point drop in mood from the previous week’s average).

		β (95% CI)	*P* value	*Q* value^a^
Intercept		2.003 (1.745 to 2.261)	<.001	<0.001
Session time point		−.0016 (−.0018 to −.0014)	<.001	<0.001
Baseline mood		−.0144 (−.0294 to .0006)	.06	0.271
Session length (min)		−.0017 (−.0048 to .0014)	.29	0.538
Days per week		−.0254 (−.0421 to −.0088)	.003	0.028
Days since last meditation		−.0652 (−.0774 to −.0530)	<.001	<0.001
Practice period		−.0081 (−.0119 to −.0044)	<.001	<0.001
Days between practice periods		.0012 (−.0061 to .0085)	.75	0.885
Exteroceptive to interoceptive session ratio		−.0010 (−.0034 to .0014)	.42	0.700
Number of practice types completed		−.0125 (−.0178 to −.0073)	<.001	<0.001
Rating score		.0018 (−.0108 to .0145)	.78	0.885
Rating count		−.0072 (−.0432 to .0289)	.70	0.870
Play count		−.0133 (−.0510 to .0245)	.49	0.737
Age		−.0098 (−.0228 to .0031)	.14	0.358
**Reason for meditating**				
	Anxiety (yes)	.0197 (−.0088 to .0481)	.18	0.420
	Stress (yes)	−.0151 (−.0425 to .0123)	.28	0.538
	Sadness (yes)	.0219 (−.0047 to .0484)	.11	0.304
	Well-being (yes)	−.0051 (−.0305 to .0202)	.69	0.870
**Time of day**				
	Night	—^b^	—	—
	Day	.0008 (−.0397 to .0413)	.97	0.986
	Morning	−.0094 (−.0396 to .0209)	.54	0.784
**Previous experience types**				
	No experience	−.0576 (−.1100 to −.0053)	.03	0.244
	Meditation apps	−.0380 (−.0784 to .0023)	.07	0.271
	Local class	−.0023 (−.0308 to .0261)	.87	0.926
	Web-based course	−.0168 (−.0458 to .0122)	.26	0.538
	Retreats	.0140 (−.0217 to .0498)	.44	0.716
	Mentoring	−.0096 (−.0439 to .0246)	.58	0.792
**Meditation orientation**				
	Positivity based	—	—	—
	Niches	.0276 (−.0199 to .0750)	.25	0.538
	Problem focused	.0000 (−.0317 to .0318)	>.99	0.998
	Techniques	.0123 (−.0375 to .0621)	.63	0.838
**Practice types**				
	Breathing meditation	—	—	—
	Body scan	−.0060 (−.0832 to .0712)	.88	0.926
	Compassion meditation	.0101 (−.0595 to .0797)	.78	0.885
	Contemplation	.0585 (−.0085 to .1256)	.09	0.271
	Guided imagery	.0530 (.0002 to .1057)	.049	0.267
	Loving-kindness (metta)	.0985 (−.0256 to .2225)	.12	0.326
	MBCT^c^ or MBSR^d^	.1190 (−.0188 to .2568)	.09	0.271
	Mindfulness meditation	.0302 (−.0240 to .0845)	.28	0.538
	Other	.0454 (−.0014 to .0921)	.06	0.271
	Positive affirmations	.0685 (.0054 to .1316)	.03	0.244
	Relaxation meditation	.1238 (.0015 to .2461)	.047	0.267
	Vipassana	.1750 (−.0170 to .3669)	.07	0.271
**Meditation worldview**				
	Hinduism	—	—	—
	Buddhism	.0730 (−.1658 to .3119)	.55	0.784
	Christianity	−.0588 (−.3221 to .2044)	.66	0.862
	Judaism	−.0208 (−.5908 to .5491)	.94	0.975
	Modernism	.0415 (−.1940 to .2770)	.73	0.885
	Niches	.0841 (−.1521 to .3203)	.49	0.737
	Other	.0685 (−.1664 to .3035)	.57	0.792
	Taoism	−.2478 (−.8230 to .3273)	.40	0.700
**Mood attributions**				
	Exercise	−.0102 (−.0828 to .0623)	.78	0.885
	Family	.0385 (−.0052 to .0822)	.08	0.271
	Finances	.0363 (−.0275 to .1001)	.27	0.538
	Food	−.0482 (−.1219 to .0256)	.20	0.463
	Friends	−.0585 (−.1236 to .0065)	.08	0.271
	Health	.0392 (.0024 to .0759)	.04	0.244
	Relationships	.0144 (−.0235 to .0523)	.46	0.720
	Sleep	−.0368 (−.0771 to .0036)	.07	0.271
	Spirituality	.0451 (−.0185 to .1086)	.16	0.411
	Studies	−.0362 (−.1099 to .0376)	.34	0.612
	Travel	−.0113 (−.1537 to .1311)	.88	0.926
	Work	.0147 (−.0208 to .0501)	.42	0.700

^a^False discovery rate correction for multiple testing.

^b^Variable reference level.

^c^MBCT: mindfulness-based cognitive therapy.

^d^MBSR: mindfulness-based stress reduction.

#### Adherence

**Figure 1:**
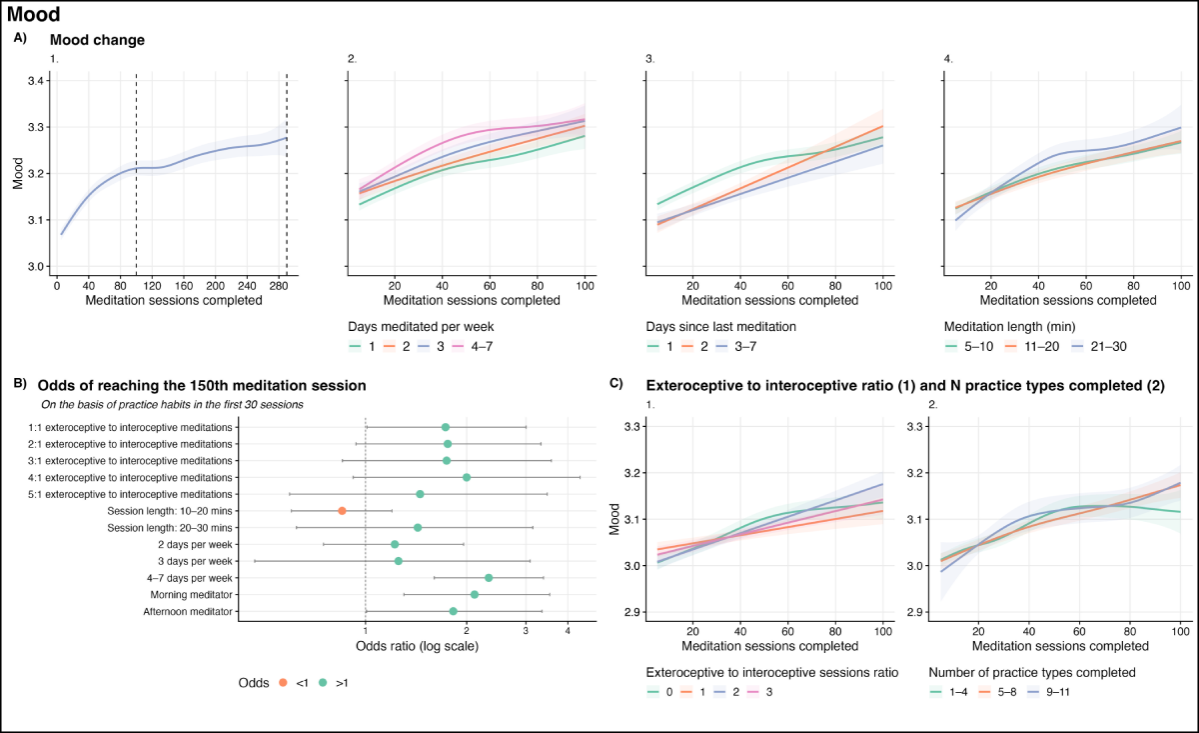
Nonlinear mood change over time by practice habits (A and C) and the odds of adherence (reaching the 150th meditation session; B).

Meditators with a balanced practice (completed 1 exteroceptive meditation for every 1 interoceptive meditation) in their first 30 sessions were 1.73 times as likely to make it to their 150th meditation session (odds ratio 1.73; *P*=.048) compared with those who predominantly completed interoceptive meditations. Those who meditated 4 to 7 days per week were 2.3 times more likely to make it to their 150th session compared with those who only meditated 1 day per week (*P*<.001). Compared with those who meditated in the evening, morning and daytime meditators were 2.1 (*P*=.003) and 1.8 (*P*=.049) times as likely to make it to the 150th meditation session, respectively. Finally, there were no significant differences in adherence across practice types, orientation, or worldview; the number of practice types completed; or session length. In addition, there were no significant differences in adherence for meditators’ baseline mood, mood attributions, previous experience, or reason for meditating. See Figure 1 and Table 6 for all results.

**Table 6:** Logistic regression coefficients for adherence. Odds ratios (ORs) represent changes in each variable in the first 30 meditation sessions and the subsequent odds of making it to the 150th meditation session (yes or no).

			OR (95% CI)		*P* value		*Q* value^a^
**Exteroceptive to interoceptive session ratio**							
	0:1	—^b^		—		—	
	1:1	1.730 (1.011-3.004)		.048		0.531	
	2:1	1.756 (0.9363-3.327)		.08		0.531	
	3:1	1.743 (0.8532-3.573)		.13		0.594	
	4:1	1.999 (0.9188-4.347)		.08		0.531	
	5:1	1.453 (0.5942-3.473)		.41		0.952	
	6:1	1.056 (0.4546-2.408)		.90		0.996	
	7:1	0.2100 (0.0106-1.330)		.17		0.659	
	8:1	1.571 (0.6070-3.992)		.35		0.952	
	9:1	4.168 (0.7453-23.70)		.10		0.594	
Baseline mood			1.187 (0.9461-1.494)		.14		0.594
**Session length (min; factorized)**							
	5-10	—		—		—	
	10-20	0.8517 (0.6016-1.199)		.36		0.952	
	20-30	1.430 (0.6228-3.146)		.38		0.952	
**Days per week (factorized)**							
	1	—		—		—	
	2	1.222 (0.7497-1.958)		.41		0.952	
	3	1.253 (0.4680-3.087)		.64		0.996	
	4-7	2.326 (1.601-3.385)		<.001		<0.001	
**Days since last meditation (factorized)**							
	1	—		—		—	
	2	0.5333 (0.2611-1.005)		.07		0.531	
	3-4	0.5571 (0.1542-1.560)		.31		0.952	
	5-7	0.0000 (0.0000-189,68^+04^)		.98		0.996	
Practice period			0.5978 (0.5361-0.6615)		<.001		<0.001
Days between practice periods			1.001 (0.992-1.010)		.75		0.996
**Number of practice types completed (factorized)**							
	1-4	—		—		—	
	5-8	1.026 (0.6486-1.641)		.92		0.996	
	9-12	1.194 (0.6374-2.241)		.58		0.996	
Rating score			0.8350 (0.0997-7.466)		.87		0.996
Rating count			1.000 (1.000-1.000)		.31		0.952
Play count			1.000 (1.000-1.000)		.52		0.996
Age (years)			1.025 (1.014-1.037)		<.001		<0.001
Session count			0.997 (0.973-1.021)		.81		0.996
Mood score			1.053 (0.7565-1.467)		.76		0.996
**Reason for meditating**							
	Anxiety	1.087 (0.7738-1.533)		.63		0.996	
	Stress	0.8699 (0.6297-1.204)		.40		0.952	
	Sadness	1.002 (0.7251-1.381)		.99		0.996	
	Well-being	1.035 (0.7648-1.405)		.83		0.996	
**Time of day**							
	Night	—		—		—	
	Day	1.824 (1.007-3.349)		.049		0.531	
	Morning	2.110 (1.300-3.532)		.003		0.059	
**Previous experience types**							
	No experience	0.8550 (0.4625-1.587)		.62		0.996	
	Meditation apps	0.992 (0.6209-1.615)		.97		0.996	
	Web-based course	1.360 (0.966-1.909)		.08		0.531	
	Local class	0.972 (0.6868-1.371)		.87		0.996	
	Retreats	0.996 (0.6490-1.514)		.99		0.996	
	Mentoring	0.958 (0.6274-1.448)		.84		0.996	
**Meditation orientation**							
	Positivity based	—		—		—	
	Niches	0.7934 (0.4176-1.473)		.47		0.996	
	Problem focused	1.029 (0.7091-1.489)		.88		0.996	
	Techniques	1.513 (0.8783-2.573)		.13		0.594	
**Practice types**							
	Breathing meditation	—		—		—	
	Body scan	0.9458 (0.2895-2.840)		.92		0.996	
	Compassion meditation	1.659 (0.5185-4.888)		.37		0.952	
	Contemplation	0.8918 (0.2426-2.874)		.85		0.996	
	Guided imagery	1.176 (0.6182-2.261)		.62		0.996	
	MBCT^c^ or MBSR^d^	0.992 (0.1063-6.167)		.99		0.996	
	Mindfulness meditation	1.118 (0.6316-2.015)		.71		0.996	
	Other	1.225 (0.6897-2.217)		.50		0.996	
	Positive affirmations	1.894 (0.8516-4.196)		.12		0.594	
	Relaxation meditation	0.5310 (0.0253-3.988)		.59		0.996	
**Meditation worldview**							
	Modernism	—		—		—	
	Buddhism	0.7467 (0.2973-1.714)		.51		0.996	
	Christianity	0.7450 (0.0966-3.775)		.74		0.996	
	Niches	1.492 (0.7093-3.128)		.29		0.952	
	Other	1.219 (0.8067-1.856)		.35		0.952	
**Mood attributions**							
	Work	0.9359 (0.6530-1.332)		.72		0.996	
	Exercise	0.7836 (0.4647-1.297)		.35		0.952	
	Family	1.329 (0.9089-1.932)		.14		0.594	
	Finances	1.205 (0.6759-2.099)		.52		0.996	
	Food	0.5441 (0.2757-1.033)		.07		0.531	
	Friends	1.095 (0.6878-1.723)		.70		0.996	
	Health	1.047 (0.7212-1.510)		.81		0.996	
	Relationship	1.106 (0.7478-1.625)		.61		0.996	
	Sleep	1.173 (0.7901-1.728)		.42		0.952	
	Spirituality	0.9386 (0.5828-1.491)		.79		0.996	
	Studies	0.7134 (0.3631-1.337)		.31		0.952	
	Travel	1.034 (0.4491-2.224)		.93		0.996	

^a^False discovery rate correction for multiple testing.

^b^Variable reference level.

^c^MBCT: mindfulness-based cognitive therapy.

^d^MBSR: mindfulness-based stress reduction.

### Nonlinear Outcome Change

**Figure 2:**
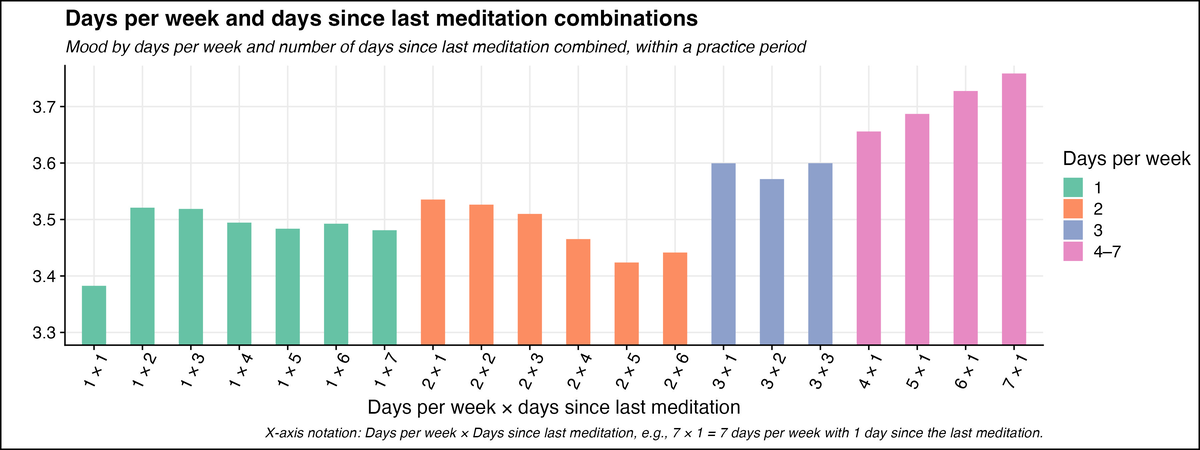
Average mood by days per week and days since last meditation session combinations. X-axis notation: days per week × days since last meditation (eg, 7 × 1 = 7 d/wk with 1 d since the last meditation). Overall, more consistent practices were associated with higher moods.

**Figure 3:**
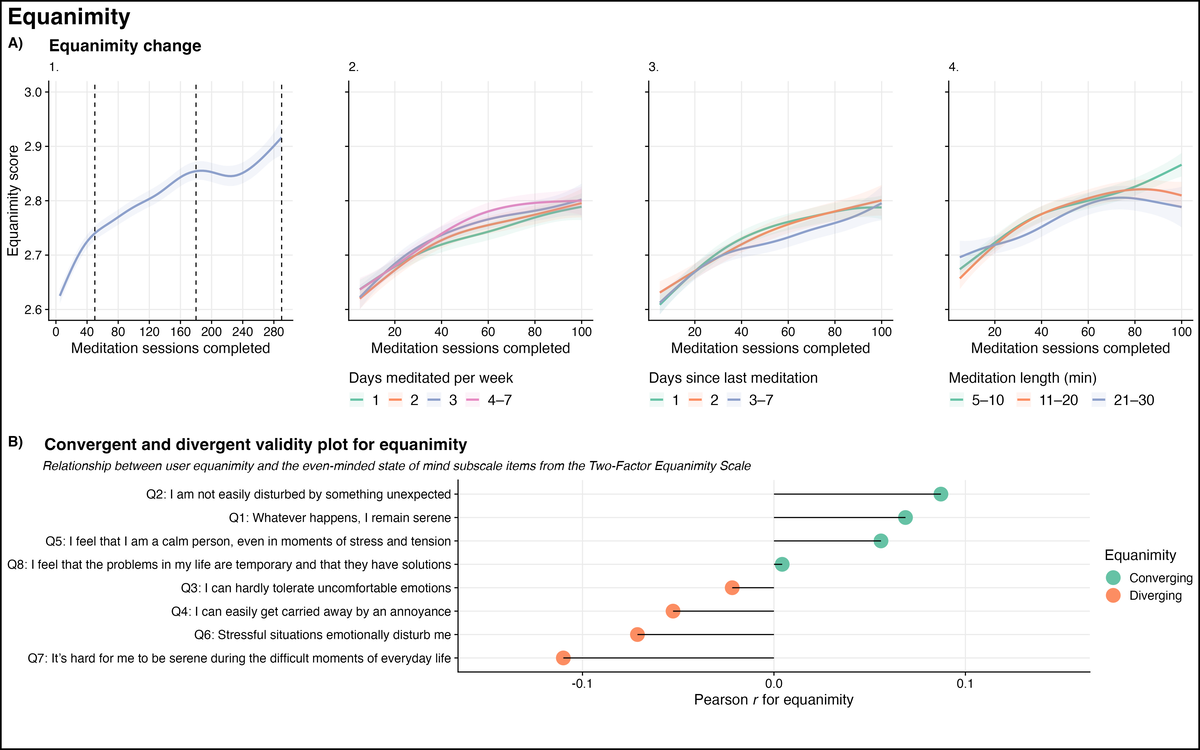
Nonlinear equanimity change over time by practice habits (A) and convergent and divergent validity using the Two-Factor Equanimity Scale even-minded state of mind subscale (B).

Building on these findings, we observed significant nonlinear relationships between our predictor and outcome variables. After controlling for the full covariate set, smoothed regression splines revealed that the largest gains in mood, equanimity, and resilience were observed within approximately the first 100 sessions, where up to 50% of improvement was achieved. These improvements were sustained and continued until the 290th session, at which point improvements were still increasing (*P*<.001 for all outcomes; graph 1 in Figures 1A, 2A, and 3A).

Improvements in outcomes were noted regardless of the number of days meditated per week. However, compared with a practice of 1 day per week, 4 to 7 days was associated with the highest mood scores in the following sessions for all time points, followed by 2 and 3 days per week (*P*<.001 for all levels). These observations were further supported when looking at the number of days since a meditator’s last session. Compared with 1 day since the last meditation session (eg, meditating over consecutive days), 2 and 3 to 7 days since the previous sessions were associated with lower mood scores over time (*P*<.001 and *P*=.02, respectively; graphs 2 and 3 in Figure 1A). See Figure 2 for average mood by days per week and days since last meditation session combinations.

A similar pattern was observed for equanimity; however, the largest increases were observed in the first 50 sessions. These improvements were sustained and continued until the 290th session, at which point improvements were still increasing (*P*<.001). As meditators progressed and completed more sessions, the largest increases in equanimity were observed from the 50th session onward for meditators practicing 4 to 7 days per week compared with those practicing 1 day per week (*P*<.001). Practicing 2 and 3 days per week followed the same trend (*P*<.001); however, the effects were smaller. The number of days since the last meditation session followed this same trend. Compared with 1 day since the last session, equanimity scores were significantly lower for 2 and 3 to 7 days (*P*<.001; graphs 2 and 3 in Figure 3A).

**Figure 4:**
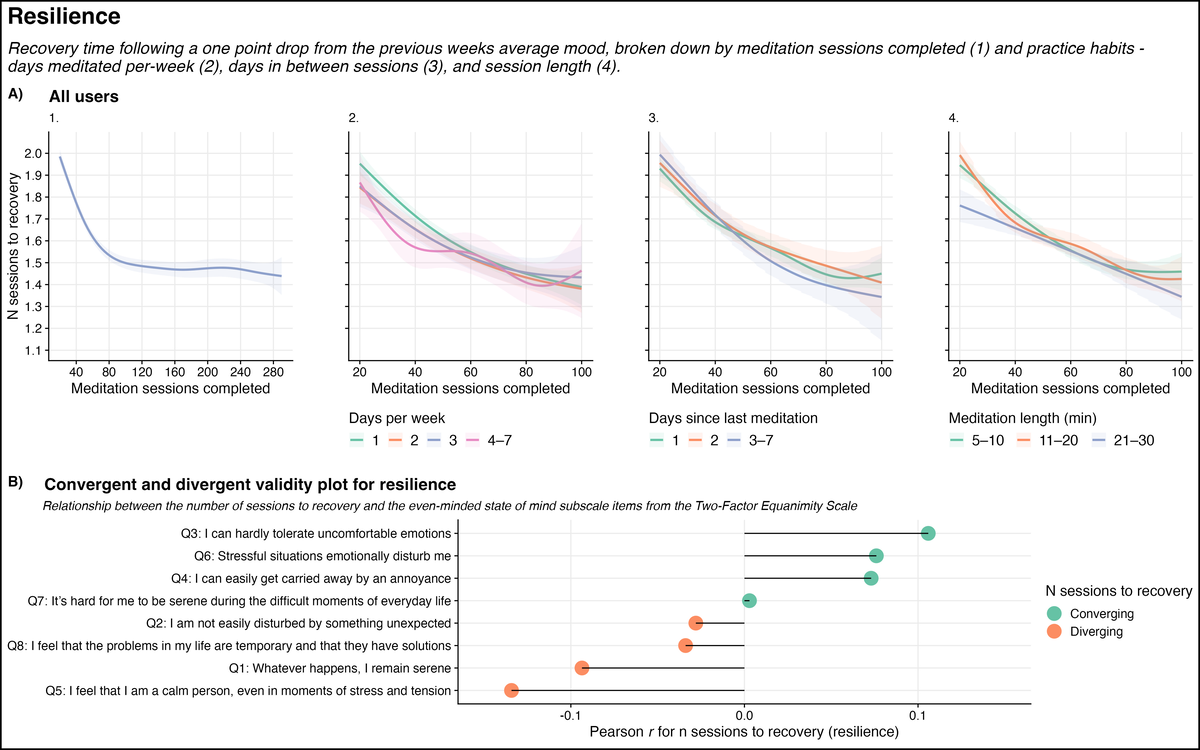
Nonlinear resilience change over time by practice habits (A) and convergent and divergent validity using the Two-Factor Equanimity Scale even-minded state of mind subscale (B).

For the number of sessions to recovery following a drop in mood (resilience), most of the effect was observed within the first 80 to 100 sessions (*P*<.001). These improvements were maintained through to the 290th session. Recovery times decreased consistently for all levels of days meditated per week and days since the last session. Until the 40th session, this effect was largest for more consistent practices (4-7 d/wk and 1 d since the previous meditation session; *P*<.001). From the 40th session onward, recovery rates were similar for all levels of practice (graphs 2 and 3 in Figure 4A). 

Although there were no significant relationships between session length and outcome change in the linear models, plotting change over time by session length in nonlinear models revealed significant and varied relationships across outcomes. Meditations of all lengths were associated with improvements in outcomes (*P*<.001). Shorter and midlength sessions (5-10 and 11-20 min) were associated with higher mood scores until the 20th meditation session, whereas longer sessions (21-30 min) were associated with higher moods from the 20th to the 290th session when compared with 5- to 10-minute sessions (*P*<.001; graph 4 in Figure 1A).

Equanimity changes were similar for all session lengths over time; however, shorter sessions were associated with higher equanimity scores from the 80th session onward (*P*<.001) when compared with longer ones (21-30 min). Faster recovery following a mood drop (resilience) was associated with longer session lengths at all time points (11-20–min sessions: *P*<.001; 21-30–min sessions: *P*=.001) when compared with 5- to 10-minute sessions (graph 4 in Figures 3A).

Finally, we observed significant nonlinear differences for different exteroceptive to interoceptive meditation ratios over time. Compared with those who predominantly completed interoceptive meditations, those who completed a higher portion of exteroceptive meditations (specifically, 2:1 exteroceptive to interoceptive) had higher mood scores from the 80th session onward (*P*<.001). In addition, a higher number of practice types was associated with higher mood scores from the 80th session onward (5-8 practice types: *P*<.001; 9-11 practice types: *P*=.001; graphs 1 and 2 in Figure 1C).

## Discussion

### Principal Findings

This study showed meaningful and significant longitudinal associations between digital meditation practice and outcome change in the largest ecologically valid sample to date. After controlling for a large range of covariates, we found that consistency of practice and balance between internally and externally focused meditations were the strongest predictors of outcome change and adherence in linear models. Breaking down meditators’ trajectories by their practice habits in nonlinear models confirmed these findings and revealed further idiosyncrasies across practice habits and outcome changes over time, particularly in relation to session length, ratios of internally to externally focused meditations, and the number of distinct practice types completed.

Overall, we observed an approximate mood score increase of 0.2 to 0.3 over the study period (Figures 1 and 2). Placing this change in the context of real-world mood improvement corresponds to approximately 1 extra day of improved mood for every 5 meditation sessions completed at the end of the study period. For example, if a meditator checked in with a score of 3 (*feeling OK*) across 5 meditation sessions, this would result in an average mood score of 3. If the same meditator then checked in with a mood score of 3 for 4 out of 5 meditations and a score of 4 (*feeling good*) for 1 out of 5 meditations, this would correspond to an average mood score of 3.2 for a total change magnitude of 0.2, as was observed over this study period. Given that the study sample comprised a nonclinical population (not specifically targeted to meditators experiencing psychopathology), this degree of improvement is arguably of both statistical and real-world significance.

More broadly, our findings tell an interesting story in relation to a meditator’s emotional change over the course of their practice. Specifically, although overall mood increased consistently at all time points, practice consistency (4-7 days meditated per week) first afforded faster mood recovery (increased resilience), which then transitioned to increased mood stability (equanimity) over time (from the 40th session onward). This pattern of change suggests that increased resilience may act as a prerequisite building block for the attainment of increased equanimity later in a meditator’s practice. Interestingly, a similar multiphasic pattern of change was observed in a cohort of participants engaged in a 6-month web-based coaching program. In the first 3 months, larger increases were observed in emotional regulation, prospection, self-awareness, self-efficacy, social connection, and stress management, whereas in the following 3 months, larger increases were observed in life satisfaction, purpose and meaning, and resilience [41].

### Comparison With Prior Work

Placing these dosage and practice habit findings in the context of previous literature helps disentangle some of the mixed results of previous dose-response studies. For example, the meta-regression by Strohmaier [5] found generally conflicting results across studies, with some favoring longer sessions, others favoring shorter ones, and others finding no significant differences. Following this meta-regression, Strohmaier et al [10] conducted an RCT finding that shorter meditation sessions were significantly associated with decreased stress in beginner meditators yet observed no differences across psychological outcomes. In our analyses, our linear models aligned with the subset of literature that found no significant differences between session length and overall change. However, when studying and plotting nonlinear curves of change over time, we observed an association between marginally higher mood scores and shorter session lengths over the first 20 sessions of practice. This might explain the findings of Strohmaier et al [10] given that their RCT was only 2 weeks in duration. Moreover, we then observed the largest statistically significant positive associations between longer meditation sessions and mood change from the 20th session onward. Therefore, the relationship between outcome change and session length may be dependent on how far a meditator has progressed in their current practice. Thus, session length effects may not emerge until a requisite amount of practice has been completed. Future RCTs should aim to extend their window of observation and consider graded progression of dosage as meditators gain experience.

Overall, our findings suggest that it was the consistency of practice, not the length of individual sessions, that was the most important predictor of change. Previous studies have found that many beginner meditators have difficulty sustaining a meditative practice when forced to complete longer sessions [5,42,43]. Although the consensus is that higher dosages are associated with more favorable outcomes [44], our results suggest that shorter sessions may be better for beginner meditators and midlength and longer sessions may be better for meditators as they gain experience [5,10]. Moreover, given the self-regulatory nature of digital practice, it seems that some meditators are capable of effectively self-regulating the required practice amounts and session lengths. It may be that those who have less favorable outcomes or churn are attempting to complete patterns of practice beyond their acquired ability. Given this potential dissonance and the high rates of attrition seen in structured programs of longer lengths (eg, mindfulness-based stress reduction and mindfulness-based cognitive therapy programs) [43], it may be prudent for practitioners to encourage consistency and longevity of practice over length and tailor programs to individuals’ needs.

Regarding objects of focus, predominantly completing exteroceptive meditations from the 80^th^ session onward was associated with the highest mood scores (specifically, a ratio of 2:1 exteroceptive to interoceptive meditations). Moreover, a balanced practice of interoceptive and exteroceptive meditations (1:1) was associated with the highest odds of adherence. Studying the characteristics of meditators predominantly completing either meditation type revealed that those meditating for anxiety were significantly less likely to predominantly practice exteroceptive meditations, whereas those meditating for sadness were significantly more likely to predominantly practice exteroceptive meditations (see the supplementary results section in Multimedia Appendix 1). An explanation may be that those meditating for anxiety were more likely to practice internally focused meditations as these allow the meditator to manage, decenter, accept, and let go of feelings of anxiety in their body. On the contrary, those meditating for sadness may be more likely to practice externally focused meditations as these allow them to break out of internal states of rumination and reorient themselves to a healthier state of mind. Clinical and neurobiological findings lend support to this view.

Mechanistically, deficits in interoception (awareness of internal bodily states) are associated with hypoactivation of the insular cortex, a brain structure that corresponds to perceptual self-awareness [14]. For meditators with heightened anxiety and interoceptive deficits who avoid or fight internal anxiety states, reorienting, accepting, and diffusing these sensations may provide a way to relieve this anxiety and balance insular cortex activation. For example, de Jong et al [45] found that increases in interoception were associated with greater well-being in patients with chronic pain who avoided sensations in their bodies. Although it is a distinct pathology, it shares significant comorbidity with anxiety disorders [46]. In addition, avoidance and a lack of decentered acceptance of anxiety states have been shown to moderate anxiety symptoms (known as *experiential avoidance*) [47-49]. This observation and a subsequent intervention composed of interoceptive awareness combined with acceptance of the bodily experience are core tenants of acceptance and commitment therapy, a clinically validated mindfulness-based intervention targeted at treating anxiety disorders [49-51].

In contrast, ruminative behaviors are a common symptom of depression, leaving individuals unable to disengage from the internal processing of maladaptive thought patterns [52]. Research suggests that an altered basal neural resting state, characterized by increased neural activity in brain regions encompassing the default mode network, might account for this increased internal self-focus [12,53]. Moreover, hyperactivation of the insular cortex, a component of the default mode network, has been linked to greater depressive rumination [11] and increased self-focus [54] and sadness [14]. Therefore, practicing exteroceptive meditations may assist those meditating to manage feelings of sadness in breaking maladaptive patterns of self-focus and rumination and reorient to a healthier state of mind [55].

Alternatively, maintaining a balanced practice composed of different mindfulness-related processes might help counterbalance the effects of different practice types [14]. For example, rather than purely focusing on the most common forms of meditation practiced in the West (eg, mindfulness of the breath and body scans [interoceptive practice types]), exploring a broader range of practices that orient the meditator to external stimuli (exteroceptive practices), such as loving-kindness meditation, metta meditation, mantras, or general objects of focus outside the body, may help counterbalance the effects of interoceptive practices and create a healthy balance of focus and insular cortex activation [14]. When focusing on adherence, our results support this hypothesis, finding that adhering to a long-term practice (reaching at least the 150th session) was best predicted by having a meditation practice that was equally balanced between exteroceptive and interoceptive (1:1) meditations. In addition to objects of focus, we observed that meditators completed 1392 unique practice type combinations across the study period, suggesting that they completed practices that were unique to their preferences and characteristics. For meditators who made it past the 80^th^ session, completing a higher number of practice types was significantly associated with the largest improvements in mood (after controlling for the total number of completed sessions and all other covariates). Thus, those who chose to branch out and explore new practices as they gained experience were likely to observe the largest mood improvement.

### Future Directions

Although there appears to be a benefit in performing a diverse range of practice types with distinct objects of focus, future works should explore how other components of meditation might effect change. Previous research suggests that different meditations contain distinct cognitive and therapeutic mechanisms (eg, meta-awareness, cognitive reappraisal, and perspective taking), which may help explain their therapeutic efficacy [13]. Identifying and studying which therapeutic mechanisms are most effective for whom and how should be explored in future studies. To do this accurately at scale will require the use of topic modeling and machine learning approaches to identify semantically similar therapeutic mechanisms and objects of focus and for whom they are best suited.

In addition to exploring these mechanisms, different meditations contain distinct nontherapeutic components related to their cultural, religious, spiritual, and historical underpinnings that assist in delivering a meditation’s therapeutic components [56]. Understanding how a nontherapeutic component might act to better deliver therapeutic mechanisms may help further explain our findings and improve the personalization of meditations for distinct groups of meditators (eg, from different cultural and religious backgrounds). This could also lead to the creation of entirely new meditative practices that are optimized for distinct mental and emotional needs, cultural and religious characteristics, and other transdiagnostic characteristics of meditators. This is particularly important given emerging evidence suggesting that mental health interventions adapted to cultural norms, religious and spiritual beliefs, and language preferences display superior efficacy and adherence to those of their nonadapted counterparts [57,58].

Finally, we observed that those who predominantly completed their meditation practice in the morning were more than twice as likely to adhere to their practice compared with those who practiced in the evening (see the supplementary results in Multimedia Appendix 1). Although there is a lack of literature pertaining to optimal practice times, yogic philosophy purports that morning practices completed in the “ambrosial hours” (2.5 hours before sunrise) are best suited to contemplative practice [59]. Interpreted through a secular lens, this may be due to the stillness and lack of interruption in these hours as well as meditating with the intention of completing practice for practice’s sake rather than in response to stressful phenomena or life events. However, it will be important for future studies to disentangle the extent to which morning practices are causally related to adherence or, instead, are a biproduct of hidden variables (eg, better mental health or personality characteristics) that might explain this result.

### Strengths

Overall, this is the largest study of longitudinal meditation effectiveness and adherence to date. We maximized the external and ecological validity of our findings through the use of instantaneous in-app reporting and global participation of meditators and validated our resilience and equanimity outcomes on a validated scale [30]. The use of EMA in a naturalistic setting helped address the memory and social desirability biases that have been prevalent in previous research (eg, the retrospective overinflation of session lengths to appeal to instructors and inaccurate recollection of session lengths and counts because of retrospective reporting) [5]. We were also able to capture, engineer, and interpret a range of meaningful covariates and confounds and assess their impact on the trajectories of meditators over time. We engineered novel features to deal with intermittent patterns of meditation and used nonlinear methods to understand the impacts of dosage and practice habits at particular points in a meditator’s progression.

### Limitations

This study has a number of limitations. First, those who consented to the use of their in-app and survey data likely represent a nonrandom sample of both Insight Timer meditators and meditators more broadly. In addition, those who used the mood check-in feature again likely represent a more homogeneous subsample of meditators, for example, those who are biased toward meditating explicitly for mood improvement or outcome change. Although we engineered additional features, meditators disinterested in mood tracking and its perceived utility may have opted out of its use, leading to a systematic underrepresentation of users from different facets of the population. Second, although we controlled for meditators’ onboarding characteristics (reason for use, previous experience types, and age), other user characteristics such as gender, socioeconomic status, length of experience in months or years, and psychopathology were not collected during onboarding because of their sensitivity.

To overcome these limitations, future works should aim to study longitudinal effectiveness and dose-response in controlled environments using detailed multi-item measures without the use of a convenience sample and routine data. For example, assessing whether our findings replicate on a random sample of meditators would increase confidence in our findings. Failure to replicate might imply true failure (eg, type 1 error) or the presence of distinct subsets of meditators who have different outcomes based on their transdiagnostic and mental characteristics. Third, although this work has high external and ecological validity given the large sample of diverse global meditators, it is possible that these effects may only be specific to digital meditation rather than in-person meditations guided by a teacher, for example, at a meditation retreat. Future works should aim to assess the generalizability of these findings to face-to-face teaching contexts. Finally, as this work was exploratory in nature, our interpretations relied on the nominal significance of the findings (although corrected *P* values were provided). Future studies aiming to replicate these findings should define a priori hypotheses and correct for multiple comparisons where necessary.

### Conclusions

In conclusion, we found that a higher number of meditation sessions and a consistent daily practice were associated with the highest mood scores, most stable moods (equanimity), and fastest mood recovery over time (resilience). Increased session lengths as meditators gained experience also showed associations with higher mood and faster recovery times. Future works should aim to replicate these findings in controlled study environments and explore other avenues of personalization based on the therapeutic mechanisms of meditations and the clinical and transdiagnostic characteristics of meditators.

## Appendix

Multimedia Appendix 1 Supplementary information, tables, and figures related to the study’s methodology and results.

## Abbreviations

EMA: ecological momentary assessment
RCT: randomized controlled trial
SIM: single-item mood

## Data Availability

The data sets analyzed in this study are not publicly available because of user agreements and international user privacy laws. However, where informed consent is provided by a user, data are made available to Insight Timer research partners.
