# Supplementary material for: The Effects of Dose, Practice Habits, and Objects of Focus on Digital Meditation Effectiveness and Adherence: Longitudinal Study of 280,000 Digital Meditation Sessions Across 103 Countries
Source: J Med Internet Res. 2023 Sep 19;25:e43358. doi: 10.2196/43358 (PMC10548318; doi:10.2196/43358)

**Supplementary Appendix**

**Learning from over 280,000 digital meditation sessions across 103 countries: A longitudinal study of digital meditation efficacy, adherence, dose, practice habits, and objects of focus**

Micah Cearns^1^, Scott Clark^2^

^1^Insight Timer Research, Insight Timer, Sydney, NSW, Australia

^2^Discipline of Psychiatry, School of Medicine, University of Adelaide, Adelaide, SA, Australia

**Supplementary methods.**

**Table S1.** Combinations and relative frequencies of each meditation session time point and days per-week level.

**Table S2.** Combinations and relative frequencies of each meditation session time point and days since last meditation session level.

**Table S3.** Combinations and relative frequencies of each meditation session time point and session length factor level.

**Table S4.** Participant count and average mood (sd) by country.

**Table S5.** Continuous variable descriptive statistics for the Two Factor Equanimity Scale.

**Table S6.** Categorical variable descriptive statistics for the Two Factor Equanimity Scale.

**Table S7.** Continuous variable descriptive statistics for the adherence model sample.

**Table S8.** Categorical variable descriptive statistics for the adherence model sample.

**Table S9.** Continuous variable descriptive statistics for the binary interoceptive vs exteroceptive meditator sample.

**Table S10.** Categorical variable descriptive statistics for the binary interoceptive vs exteroceptive meditator sample.

**Figure S1.** Insight Timers in-app mood check-in screen.

**Figure S2.** User onboarding flow.

**Figure S3.** Schema for practice periods and shifted mood check-ins.

**Figure S4.** Distribution of the number of days since a user’s last meditation session.

**Figure S5.** Distribution of mood check-in counts by user and practice period.

**Figure S6.** Average number of mood check-ins per-week.

**Figure S7.** Cumulative number of meditation sessions distribution.

**Figure S8.** Counts and categorisation of interoceptive and exteroceptive meditations.

**Figure S9.** Session time point distribution pre and post trimming with the boxplot method and after harmonisation to nearest values of 5.

**Figure S10.** Session length distribution pre and post trimming with the boxplot method (A and B) and after harmonisation to nearest values of 5 (C).

**Figure S11.** Relative frequency plots for each session time point and practice habit level combinations from Tables S1 to S3.

**Supplementary methods**

**1. Single item scales – Previous uses, predictive, convergent, divergent, and concurrent validity**

Previous work by van Rijsbergen et al [31] found that SIMs were predictive of relapse in major depression and showed higher area under the receiver operator characteristic curve and positive predicted values than the clinically validated 17 item Hamilton Depression Rating Scale and the Inventory of Depressive Symptomology. In addition, Killgore [32] found that a SIM could classify depressed mood with a balanced accuracy of 72% compared to the full Profile of Mood States Depression scale, which achieved a balanced accuracy of 73.5%. Overall, the SIM significantly discriminated subjects by mood category and achieved a comparable hit rate, sensitivity, and specificity relative to other well validated mood scales^2^.

While not a SIM, work by Abdel-Khalek [33] found that a single item happiness measure showed strong correlations with the Oxford Happiness Inventory and the Satisfaction with Life Scale, denoting good concurrent validity. Moreover, the single item had good convergent validity because it was highly and positively correlated with optimism, hope, self-esteem, positive affect, extraversion, and self-ratings of both physical and mental health. Furthermore, the divergent validity of the single item was adequately demonstrated through its significant and negative correlations with anxiety, pessimism, negative affect, and insomnia. It was concluded that measuring happiness by a single item was reliable, valid, and viable in community surveys as well as in cross-cultural comparisons.

Finally, de Boer et al [34], found that a single item quality of life measure showed high correlations with the Medical Outcomes Study Short Form-20 scale and the Rotterdam Symptom Check-List (RSCL) in oesophageal adenocarcinoma patients. Concluding that repeated measures of quality of life with a single item had good validity, excellent reliability, moderate distribution-based responsiveness, and good anchor-based responsiveness compared to multi-item questionnaires.

**2. The Two-Factor Equanimity Scale**

To measure user’s equanimity, we used the Two-Factor Equanimity Scale. This scale was constructed to measure equanimity based on a literature review of different conceptualisations of equanimity and its associated items^5^. The two factors quantifying equanimity were derived via factor analysis and represent two distinct constructs: hedonic independence and even-minded state of mind. For the sake of brevity, the high risk of churn associated with mobile surveys, and the general correlations between these subscales and the FFMQ already used in this study, we chose to only use the eight Items from the Even Minded State of Mind subscale. After controlling for the hedonic independence subscale, Juneau et al [35] found that the even-minded state of mind subscale demonstrated negative correlations with measures of hyper-sensitivity and the avoidance and fusion scale and showed a strong negative correlation with neuroticism.

**3. Content categorisations**

IT content was categorised in-house by IT engineers using a three-step approach. First, content tags were provided by IT content providers (e.g., meditation teachers) when a content file was uploaded. Second, these tags were then used to start an exhaustive search process of content title keywords to both validate the accuracy of the uploaded content tags and expand the range of related keywords. For example, all content containing a specific content tag was selected and titles were then searched for their most common keywords. The related keywords for that content was then expanded iteratively until the process was exhausted and all relevant keywords were retrieved for a content subset. The content containing the subset of keywords were then classified accordingly. For example, for Christian content, all content with the uploaded content tag of “Christian” was selected. Keywords in this content subset were then ranked and new relevant keywords were obtained, e.g., “Jesus”, “Christ”, “Bible”, “God” etc. This process was repeated iteratively until all relevant keywords were exhausted (e.g., now content with the keyword “Jesus” in the title was subset and new keywords were derived, and so forth). Keywords that were mutually exclusive to a content category, e.g., “Jesus”, “Christ”, “Bible” were then used to further validate and categorise “Christian” content in addition to the “Christian” content tag. A final technique was then used to validate and expand the above content categorisations using graph-based network models. Firstly, stop-words were removed from content titles and network models were then constructed using the remaining title keywords and clustered using community detection algorithms. The top unique keywords for each cluster were then ranked and used to validate and expand the above content classifications. This process was used to classify practice types, content orientations, and worldviews.

**4. Sample size analysis for the two-way interaction models**

As discussed in the main manuscript, we used the relative frequency values reported in Tables S1-3 and Figure S9 to derive total session and practice habit cut-offs for each model. To further clarify using Table S1 below, we see that the most common combinations of total sessions and days per-week is the first meditation session with 1 day meditated in that sessions given week. Relative to this combination, the next most common combination was 2 meditation sessions completed over 2 distinct weeks, with a relative frequency of 90%. Using these frequency values, we derived cut-offs for each session time point and practice habit combination to quantify their relative frequencies to the most common session time point and practice habit values. This was important as the interpretation of each point along a curve will always be made relative to earlier points along that curve yet will be partially confounded by decreasing sample size as the number of meditation sessions increases. For example, the difference in mood at the 20^th^ meditation session for those who mostly meditated twice per-week, compared to those at their 5^th^ session who mostly meditated twice per-week will vary as a function of the number of sessions, this days per-week practice habit, the full covariate set, as well as changes in sample size arising from attrition. Therefore, to balance this trade-off and ensure reliable interpretation, we used these relative frequency values to derive cut-offs for the maximum number of sessions that should be included for each session and practice habit interaction model. At the 60^th^ meditation session, we found that there were relative frequencies in the range of 2-5% for each session time point and practice habit level and tended to have at least 100 meditation sessions across each level. Therefore, we chose the 60^th^ meditation session as the cut-off point for these GAM interaction models. For a graphical representation of these relative frequency values see Table S9 below.

**Table S1.** Combinations and relative frequencies of each meditation session time point and days per-week level.

| **Session number** | **Days per-week factor** | **Mean** | **N** | **SD** | **Alpha** |
| --- | --- | --- | --- | --- | --- |
| 1 | 1 | 3.34 | 8466.00 | 0.89 | 1.00 |
| 2 | 1 | 3.35 | 4937.00 | 0.90 | 0.58 |
| 2 | 2 | 3.43 | 1988.00 | 0.84 | 0.23 |
| 3 | 1 | 3.37 | 3905.00 | 0.91 | 0.46 |
| 3 | 2 | 3.47 | 1730.00 | 0.84 | 0.20 |
| 3 | 3 | 3.53 | 614.00 | 0.81 | 0.07 |
| 4 | 1 | 3.37 | 3548.00 | 0.91 | 0.42 |
| 4 | 2 | 3.45 | 1436.00 | 0.88 | 0.17 |
| 4 | 3 | 3.48 | 622.00 | 0.85 | 0.07 |
| 4 | 4 | 3.55 | 222.00 | 0.76 | 0.03 |
| 5 | 1 | 3.37 | 3184.00 | 0.92 | 0.38 |
| 5 | 2 | 3.48 | 1340.00 | 0.84 | 0.16 |
| 5 | 3 | 3.44 | 617.00 | 0.87 | 0.07 |
| 5 | 4 | 3.53 | 357.00 | 0.84 | 0.04 |
| 6 | 1 | 3.38 | 3129.00 | 0.90 | 0.37 |
| 6 | 2 | 3.48 | 1157.00 | 0.85 | 0.14 |
| 6 | 3 | 3.53 | 562.00 | 0.82 | 0.07 |
| 6 | 4 | 3.52 | 388.00 | 0.76 | 0.05 |
| 7 | 1 | 3.40 | 2820.00 | 0.90 | 0.33 |
| 7 | 2 | 3.48 | 1243.00 | 0.84 | 0.15 |
| 7 | 3 | 3.53 | 526.00 | 0.83 | 0.06 |
| 7 | 4 | 3.49 | 418.00 | 0.86 | 0.05 |
| 8 | 1 | 3.39 | 2622.00 | 0.92 | 0.31 |
| 8 | 2 | 3.50 | 1147.00 | 0.84 | 0.14 |
| 8 | 3 | 3.46 | 551.00 | 0.84 | 0.07 |
| 8 | 4 | 3.55 | 386.00 | 0.83 | 0.05 |
| 9 | 1 | 3.38 | 2540.00 | 0.92 | 0.30 |
| 9 | 2 | 3.55 | 1059.00 | 0.85 | 0.13 |
| 9 | 3 | 3.46 | 544.00 | 0.87 | 0.06 |
| 9 | 4 | 3.61 | 403.00 | 0.83 | 0.05 |
| 10 | 1 | 3.42 | 2340.00 | 0.88 | 0.28 |
| 10 | 2 | 3.49 | 1043.00 | 0.86 | 0.12 |
| 10 | 3 | 3.53 | 549.00 | 0.90 | 0.06 |
| 10 | 4 | 3.53 | 388.00 | 0.85 | 0.05 |
| 11 | 1 | 3.39 | 2220.00 | 0.89 | 0.26 |
| 11 | 2 | 3.52 | 964.00 | 0.89 | 0.11 |
| 11 | 3 | 3.52 | 503.00 | 0.89 | 0.06 |
| 11 | 4 | 3.58 | 415.00 | 0.82 | 0.05 |
| 12 | 1 | 3.42 | 2091.00 | 0.90 | 0.25 |
| 12 | 2 | 3.50 | 944.00 | 0.83 | 0.11 |
| 12 | 3 | 3.52 | 509.00 | 0.86 | 0.06 |
| 12 | 4 | 3.61 | 417.00 | 0.85 | 0.05 |
| 13 | 1 | 3.42 | 1996.00 | 0.91 | 0.24 |
| 13 | 2 | 3.51 | 862.00 | 0.85 | 0.10 |
| 13 | 3 | 3.51 | 460.00 | 0.88 | 0.05 |
| 13 | 4 | 3.60 | 412.00 | 0.90 | 0.05 |
| 14 | 1 | 3.42 | 1893.00 | 0.91 | 0.22 |
| 14 | 2 | 3.59 | 867.00 | 0.86 | 0.10 |
| 14 | 3 | 3.55 | 452.00 | 0.77 | 0.05 |
| 14 | 4 | 3.54 | 398.00 | 0.84 | 0.05 |
| 15 | 1 | 3.39 | 1835.00 | 0.92 | 0.22 |
| 15 | 2 | 3.51 | 794.00 | 0.89 | 0.09 |
| 15 | 3 | 3.62 | 453.00 | 0.88 | 0.05 |
| 15 | 4 | 3.56 | 401.00 | 0.79 | 0.05 |
| 16 | 1 | 3.40 | 1657.00 | 0.90 | 0.20 |
| 16 | 2 | 3.52 | 868.00 | 0.89 | 0.10 |
| 16 | 3 | 3.58 | 428.00 | 0.80 | 0.05 |
| 16 | 4 | 3.62 | 413.00 | 0.82 | 0.05 |
| 17 | 1 | 3.44 | 1646.00 | 0.89 | 0.19 |
| 17 | 2 | 3.50 | 769.00 | 0.85 | 0.09 |
| 17 | 3 | 3.57 | 423.00 | 0.89 | 0.05 |
| 17 | 4 | 3.60 | 389.00 | 0.85 | 0.05 |
| 18 | 1 | 3.44 | 1576.00 | 0.90 | 0.19 |
| 18 | 2 | 3.53 | 764.00 | 0.83 | 0.09 |
| 18 | 3 | 3.63 | 417.00 | 0.85 | 0.05 |
| 18 | 4 | 3.62 | 373.00 | 0.85 | 0.04 |
| 19 | 1 | 3.45 | 1566.00 | 0.90 | 0.18 |
| 19 | 2 | 3.54 | 713.00 | 0.82 | 0.08 |
| 19 | 3 | 3.54 | 393.00 | 0.84 | 0.05 |
| 19 | 4 | 3.61 | 347.00 | 0.84 | 0.04 |
| 20 | 1 | 3.42 | 1425.00 | 0.90 | 0.17 |
| 20 | 2 | 3.52 | 725.00 | 0.87 | 0.09 |
| 20 | 3 | 3.58 | 410.00 | 0.84 | 0.05 |
| 20 | 4 | 3.66 | 352.00 | 0.89 | 0.04 |
| 21 | 1 | 3.46 | 1344.00 | 0.92 | 0.16 |
| 21 | 2 | 3.56 | 739.00 | 0.84 | 0.09 |
| 21 | 3 | 3.47 | 387.00 | 0.91 | 0.05 |
| 21 | 4 | 3.55 | 359.00 | 0.85 | 0.04 |
| 22 | 1 | 3.48 | 1333.00 | 0.89 | 0.16 |
| 22 | 2 | 3.57 | 677.00 | 0.82 | 0.08 |
| 22 | 3 | 3.62 | 385.00 | 0.89 | 0.05 |
| 22 | 4 | 3.64 | 349.00 | 0.79 | 0.04 |
| 23 | 1 | 3.47 | 1292.00 | 0.90 | 0.15 |
| 23 | 2 | 3.54 | 659.00 | 0.88 | 0.08 |
| 23 | 3 | 3.56 | 376.00 | 0.85 | 0.04 |
| 23 | 4 | 3.70 | 349.00 | 0.78 | 0.04 |
| 24 | 1 | 3.48 | 1271.00 | 0.90 | 0.15 |
| 24 | 2 | 3.59 | 647.00 | 0.86 | 0.08 |
| 24 | 3 | 3.60 | 338.00 | 0.89 | 0.04 |
| 24 | 4 | 3.60 | 332.00 | 0.83 | 0.04 |
| 25 | 1 | 3.51 | 1191.00 | 0.88 | 0.14 |
| 25 | 2 | 3.54 | 621.00 | 0.86 | 0.07 |
| 25 | 3 | 3.64 | 344.00 | 0.89 | 0.04 |
| 25 | 4 | 3.65 | 360.00 | 0.82 | 0.04 |
| 26 | 1 | 3.53 | 1119.00 | 0.91 | 0.13 |
| 26 | 2 | 3.55 | 608.00 | 0.88 | 0.07 |
| 26 | 3 | 3.61 | 350.00 | 0.85 | 0.04 |
| 26 | 4 | 3.62 | 337.00 | 0.90 | 0.04 |
| 27 | 1 | 3.49 | 1144.00 | 0.91 | 0.14 |
| 27 | 2 | 3.53 | 592.00 | 0.90 | 0.07 |
| 27 | 3 | 3.50 | 317.00 | 0.86 | 0.04 |
| 27 | 4 | 3.73 | 317.00 | 0.81 | 0.04 |
| 28 | 1 | 3.50 | 1078.00 | 0.88 | 0.13 |
| 28 | 2 | 3.58 | 586.00 | 0.86 | 0.07 |
| 28 | 3 | 3.56 | 305.00 | 0.88 | 0.04 |
| 28 | 4 | 3.63 | 326.00 | 0.82 | 0.04 |
| 29 | 1 | 3.48 | 1040.00 | 0.91 | 0.12 |
| 29 | 2 | 3.52 | 539.00 | 0.88 | 0.06 |
| 29 | 3 | 3.57 | 321.00 | 0.88 | 0.04 |
| 29 | 4 | 3.69 | 336.00 | 0.83 | 0.04 |
| 30 | 1 | 3.50 | 988.00 | 0.89 | 0.12 |
| 30 | 2 | 3.54 | 528.00 | 0.92 | 0.06 |
| 30 | 3 | 3.58 | 286.00 | 0.90 | 0.03 |
| 30 | 4 | 3.66 | 355.00 | 0.82 | 0.04 |
| 31 | 1 | 3.51 | 978.00 | 0.87 | 0.12 |
| 31 | 2 | 3.57 | 513.00 | 0.87 | 0.06 |
| 31 | 3 | 3.64 | 317.00 | 0.84 | 0.04 |
| 31 | 4 | 3.69 | 310.00 | 0.86 | 0.04 |
| 32 | 1 | 3.54 | 923.00 | 0.88 | 0.11 |
| 32 | 2 | 3.54 | 508.00 | 0.79 | 0.06 |
| 32 | 3 | 3.56 | 269.00 | 0.86 | 0.03 |
| 32 | 4 | 3.72 | 317.00 | 0.86 | 0.04 |
| 33 | 1 | 3.54 | 870.00 | 0.89 | 0.10 |
| 33 | 2 | 3.59 | 534.00 | 0.83 | 0.06 |
| 33 | 3 | 3.61 | 300.00 | 0.80 | 0.04 |
| 33 | 4 | 3.70 | 307.00 | 0.87 | 0.04 |
| 34 | 1 | 3.50 | 848.00 | 0.93 | 0.10 |
| 34 | 2 | 3.62 | 472.00 | 0.83 | 0.06 |
| 34 | 3 | 3.62 | 308.00 | 0.90 | 0.04 |
| 34 | 4 | 3.74 | 318.00 | 0.76 | 0.04 |
| 35 | 1 | 3.49 | 876.00 | 0.90 | 0.10 |
| 35 | 2 | 3.51 | 452.00 | 0.86 | 0.05 |
| 35 | 3 | 3.65 | 279.00 | 0.81 | 0.03 |
| 35 | 4 | 3.79 | 301.00 | 0.78 | 0.04 |
| 36 | 1 | 3.56 | 803.00 | 0.86 | 0.09 |
| 36 | 2 | 3.55 | 496.00 | 0.88 | 0.06 |
| 36 | 3 | 3.69 | 285.00 | 0.80 | 0.03 |
| 36 | 4 | 3.73 | 297.00 | 0.87 | 0.04 |
| 37 | 1 | 3.56 | 788.00 | 0.87 | 0.09 |
| 37 | 2 | 3.52 | 435.00 | 0.86 | 0.05 |
| 37 | 3 | 3.59 | 280.00 | 0.89 | 0.03 |
| 37 | 4 | 3.76 | 303.00 | 0.89 | 0.04 |
| 38 | 1 | 3.54 | 746.00 | 0.89 | 0.09 |
| 38 | 2 | 3.62 | 433.00 | 0.88 | 0.05 |
| 38 | 3 | 3.59 | 259.00 | 0.79 | 0.03 |
| 38 | 4 | 3.71 | 308.00 | 0.87 | 0.04 |
| 39 | 1 | 3.52 | 721.00 | 0.94 | 0.09 |
| 39 | 2 | 3.59 | 422.00 | 0.89 | 0.05 |
| 39 | 3 | 3.55 | 261.00 | 0.85 | 0.03 |
| 39 | 4 | 3.73 | 295.00 | 0.82 | 0.03 |
| 40 | 1 | 3.55 | 736.00 | 0.84 | 0.09 |
| 40 | 2 | 3.58 | 417.00 | 0.85 | 0.05 |
| 40 | 3 | 3.70 | 258.00 | 0.89 | 0.03 |
| 40 | 4 | 3.73 | 265.00 | 0.78 | 0.03 |
| 41 | 1 | 3.57 | 685.00 | 0.85 | 0.08 |
| 41 | 2 | 3.60 | 439.00 | 0.80 | 0.05 |
| 41 | 3 | 3.64 | 255.00 | 0.82 | 0.03 |
| 41 | 4 | 3.67 | 287.00 | 0.84 | 0.03 |
| 42 | 1 | 3.58 | 695.00 | 0.84 | 0.08 |
| 42 | 2 | 3.66 | 393.00 | 0.90 | 0.05 |
| 42 | 3 | 3.63 | 265.00 | 0.83 | 0.03 |
| 42 | 4 | 3.70 | 266.00 | 0.83 | 0.03 |
| 43 | 1 | 3.57 | 667.00 | 0.85 | 0.08 |
| 43 | 2 | 3.62 | 403.00 | 0.88 | 0.05 |
| 43 | 3 | 3.64 | 231.00 | 0.83 | 0.03 |
| 43 | 4 | 3.60 | 281.00 | 0.81 | 0.03 |
| 44 | 1 | 3.57 | 636.00 | 0.85 | 0.08 |
| 44 | 2 | 3.65 | 405.00 | 0.80 | 0.05 |
| 44 | 3 | 3.62 | 239.00 | 0.88 | 0.03 |
| 44 | 4 | 3.69 | 252.00 | 0.79 | 0.03 |
| 45 | 1 | 3.56 | 611.00 | 0.85 | 0.07 |
| 45 | 2 | 3.59 | 393.00 | 0.84 | 0.05 |
| 45 | 3 | 3.65 | 227.00 | 0.81 | 0.03 |
| 45 | 4 | 3.71 | 284.00 | 0.80 | 0.03 |
| 46 | 1 | 3.51 | 612.00 | 0.92 | 0.07 |
| 46 | 2 | 3.64 | 345.00 | 0.86 | 0.04 |
| 46 | 3 | 3.69 | 229.00 | 0.82 | 0.03 |
| 46 | 4 | 3.67 | 274.00 | 0.88 | 0.03 |
| 47 | 1 | 3.58 | 570.00 | 0.87 | 0.07 |
| 47 | 2 | 3.62 | 357.00 | 0.83 | 0.04 |
| 47 | 3 | 3.69 | 204.00 | 0.81 | 0.02 |
| 47 | 4 | 3.68 | 265.00 | 0.81 | 0.03 |
| 48 | 1 | 3.60 | 616.00 | 0.86 | 0.07 |
| 48 | 2 | 3.57 | 341.00 | 0.84 | 0.04 |
| 48 | 3 | 3.64 | 199.00 | 0.79 | 0.02 |
| 48 | 4 | 3.65 | 235.00 | 0.83 | 0.03 |
| 49 | 1 | 3.60 | 602.00 | 0.85 | 0.07 |
| 49 | 2 | 3.62 | 324.00 | 0.89 | 0.04 |
| 49 | 3 | 3.64 | 196.00 | 0.86 | 0.02 |
| 49 | 4 | 3.74 | 254.00 | 0.78 | 0.03 |
| 50 | 1 | 3.57 | 560.00 | 0.85 | 0.07 |
| 50 | 2 | 3.61 | 329.00 | 0.83 | 0.04 |
| 50 | 3 | 3.64 | 223.00 | 0.90 | 0.03 |
| 50 | 4 | 3.87 | 229.00 | 0.78 | 0.03 |
| 51 | 1 | 3.60 | 539.00 | 0.89 | 0.06 |
| 51 | 2 | 3.66 | 346.00 | 0.79 | 0.04 |
| 51 | 3 | 3.69 | 191.00 | 0.88 | 0.02 |
| 51 | 4 | 3.74 | 238.00 | 0.81 | 0.03 |
| 52 | 1 | 3.57 | 487.00 | 0.92 | 0.06 |
| 52 | 2 | 3.65 | 328.00 | 0.88 | 0.04 |
| 52 | 3 | 3.69 | 218.00 | 0.93 | 0.03 |
| 52 | 4 | 3.81 | 253.00 | 0.78 | 0.03 |
| 53 | 1 | 3.61 | 514.00 | 0.91 | 0.06 |
| 53 | 2 | 3.69 | 319.00 | 0.86 | 0.04 |
| 53 | 3 | 3.60 | 199.00 | 0.84 | 0.02 |
| 53 | 4 | 3.72 | 237.00 | 0.79 | 0.03 |
| 54 | 1 | 3.58 | 509.00 | 0.91 | 0.06 |
| 54 | 2 | 3.68 | 305.00 | 0.87 | 0.04 |
| 54 | 3 | 3.60 | 202.00 | 0.85 | 0.02 |
| 54 | 4 | 3.61 | 231.00 | 0.79 | 0.03 |
| 55 | 1 | 3.57 | 486.00 | 0.88 | 0.06 |
| 55 | 2 | 3.73 | 289.00 | 0.85 | 0.03 |
| 55 | 3 | 3.82 | 182.00 | 0.84 | 0.02 |
| 55 | 4 | 3.75 | 231.00 | 0.83 | 0.03 |
| 56 | 1 | 3.61 | 472.00 | 0.88 | 0.06 |
| 56 | 2 | 3.64 | 285.00 | 0.88 | 0.03 |
| 56 | 3 | 3.69 | 192.00 | 0.91 | 0.02 |
| 56 | 4 | 3.75 | 235.00 | 0.85 | 0.03 |
| 57 | 1 | 3.68 | 437.00 | 0.86 | 0.05 |
| 57 | 2 | 3.62 | 301.00 | 0.90 | 0.04 |
| 57 | 3 | 3.59 | 181.00 | 0.89 | 0.02 |
| 57 | 4 | 3.78 | 229.00 | 0.80 | 0.03 |
| 58 | 1 | 3.63 | 431.00 | 0.91 | 0.05 |
| 58 | 2 | 3.66 | 279.00 | 0.92 | 0.03 |
| 58 | 3 | 3.60 | 181.00 | 0.92 | 0.02 |
| 58 | 4 | 3.67 | 255.00 | 0.83 | 0.03 |
| 59 | 1 | 3.63 | 447.00 | 0.88 | 0.05 |
| 59 | 2 | 3.64 | 249.00 | 0.85 | 0.03 |
| 59 | 3 | 3.66 | 178.00 | 0.87 | 0.02 |
| 59 | 4 | 3.63 | 228.00 | 0.83 | 0.03 |
| 60 | 1 | 3.64 | 451.00 | 0.90 | 0.05 |
| 60 | 2 | 3.59 | 254.00 | 0.87 | 0.03 |
| 60 | 3 | 3.69 | 179.00 | 0.82 | 0.02 |
| 60 | 4 | 3.75 | 214.00 | 0.82 | 0.03 |

**Table S2.** Combinations and relative frequencies of each meditation session time point and days since last meditation session level.

| **Session number** | **Days since last meditation factor** | **Mean** | **N** | **SD** | **Alpha** |
| --- | --- | --- | --- | --- | --- |
| 1 | 1 | 3.34 | 6783 | 0.89 | 0.79 |
| 1 | 2 | 3.36 | 795 | 0.87 | 0.09 |
| 1 | 3 | 3.31 | 582 | 0.86 | 0.07 |
| 1 | 4 | 3.28 | 406 | 0.90 | 0.05 |
| 2 | 1 | 3.38 | 5145 | 0.90 | 0.60 |
| 2 | 2 | 3.39 | 817 | 0.90 | 0.10 |
| 2 | 3 | 3.38 | 644 | 0.82 | 0.08 |
| 2 | 4 | 3.28 | 469 | 0.89 | 0.05 |
| 3 | 1 | 3.42 | 4669 | 0.89 | 0.55 |
| 3 | 2 | 3.41 | 796 | 0.87 | 0.09 |
| 3 | 3 | 3.34 | 574 | 0.85 | 0.07 |
| 3 | 4 | 3.31 | 377 | 0.88 | 0.04 |
| 4 | 1 | 3.42 | 4469 | 0.90 | 0.52 |
| 4 | 2 | 3.45 | 691 | 0.84 | 0.08 |
| 4 | 3 | 3.34 | 520 | 0.89 | 0.06 |
| 4 | 4 | 3.31 | 347 | 0.93 | 0.04 |
| 5 | 1 | 3.41 | 4244 | 0.89 | 0.50 |
| 5 | 2 | 3.43 | 666 | 0.88 | 0.08 |
| 5 | 3 | 3.42 | 473 | 0.91 | 0.06 |
| 5 | 4 | 3.38 | 323 | 0.93 | 0.04 |
| 6 | 1 | 3.43 | 4086 | 0.86 | 0.48 |
| 6 | 2 | 3.42 | 621 | 0.93 | 0.07 |
| 6 | 3 | 3.38 | 457 | 0.92 | 0.05 |
| 6 | 4 | 3.40 | 311 | 0.95 | 0.04 |
| 7 | 1 | 3.45 | 3972 | 0.86 | 0.46 |
| 7 | 2 | 3.44 | 610 | 0.91 | 0.07 |
| 7 | 3 | 3.39 | 413 | 0.97 | 0.05 |
| 7 | 4 | 3.39 | 306 | 0.93 | 0.04 |
| 8 | 1 | 3.45 | 3759 | 0.89 | 0.44 |
| 8 | 2 | 3.46 | 566 | 0.89 | 0.07 |
| 8 | 3 | 3.39 | 421 | 0.87 | 0.05 |
| 8 | 4 | 3.36 | 282 | 0.90 | 0.03 |
| 9 | 1 | 3.46 | 3658 | 0.89 | 0.43 |
| 9 | 2 | 3.44 | 570 | 0.90 | 0.07 |
| 9 | 3 | 3.39 | 394 | 0.91 | 0.05 |
| 9 | 4 | 3.41 | 257 | 0.93 | 0.03 |
| 10 | 1 | 3.45 | 3519 | 0.87 | 0.41 |
| 10 | 2 | 3.46 | 545 | 0.85 | 0.06 |
| 10 | 3 | 3.45 | 355 | 0.93 | 0.04 |
| 10 | 4 | 3.46 | 245 | 0.93 | 0.03 |
| 11 | 1 | 3.45 | 3375 | 0.90 | 0.39 |
| 11 | 2 | 3.42 | 525 | 0.88 | 0.06 |
| 11 | 3 | 3.45 | 316 | 0.95 | 0.04 |
| 11 | 4 | 3.49 | 244 | 0.89 | 0.03 |
| 12 | 1 | 3.49 | 3279 | 0.88 | 0.38 |
| 12 | 2 | 3.47 | 510 | 0.87 | 0.06 |
| 12 | 3 | 3.45 | 325 | 0.92 | 0.04 |
| 12 | 4 | 3.35 | 211 | 0.87 | 0.02 |
| 13 | 1 | 3.47 | 3132 | 0.89 | 0.37 |
| 13 | 2 | 3.46 | 486 | 0.93 | 0.06 |
| 13 | 3 | 3.40 | 290 | 0.94 | 0.03 |
| 13 | 4 | 3.46 | 196 | 0.83 | 0.02 |
| 14 | 1 | 3.50 | 3032 | 0.87 | 0.35 |
| 14 | 2 | 3.44 | 461 | 0.92 | 0.05 |
| 14 | 3 | 3.44 | 299 | 0.90 | 0.03 |
| 14 | 4 | 3.39 | 183 | 0.86 | 0.02 |
| 15 | 1 | 3.48 | 2957 | 0.88 | 0.35 |
| 15 | 2 | 3.42 | 416 | 0.92 | 0.05 |
| 15 | 3 | 3.47 | 292 | 0.94 | 0.03 |
| 15 | 4 | 3.32 | 186 | 0.98 | 0.02 |
| 16 | 1 | 3.50 | 2863 | 0.87 | 0.33 |
| 16 | 2 | 3.42 | 411 | 0.92 | 0.05 |
| 16 | 3 | 3.44 | 295 | 0.87 | 0.03 |
| 16 | 4 | 3.38 | 172 | 0.96 | 0.02 |
| 17 | 1 | 3.49 | 2794 | 0.88 | 0.33 |
| 17 | 2 | 3.43 | 420 | 0.93 | 0.05 |
| 17 | 3 | 3.45 | 253 | 0.86 | 0.03 |
| 17 | 4 | 3.30 | 159 | 0.93 | 0.02 |
| 18 | 1 | 3.52 | 2711 | 0.87 | 0.32 |
| 18 | 2 | 3.52 | 418 | 0.94 | 0.05 |
| 18 | 3 | 3.40 | 213 | 0.90 | 0.02 |
| 18 | 4 | 3.34 | 174 | 0.94 | 0.02 |
| 19 | 1 | 3.52 | 2629 | 0.86 | 0.31 |
| 19 | 2 | 3.47 | 401 | 0.89 | 0.05 |
| 19 | 3 | 3.44 | 225 | 0.87 | 0.03 |
| 19 | 4 | 3.33 | 150 | 0.92 | 0.02 |
| 20 | 1 | 3.51 | 2539 | 0.89 | 0.30 |
| 20 | 2 | 3.42 | 394 | 0.89 | 0.05 |
| 20 | 3 | 3.41 | 217 | 0.87 | 0.03 |
| 20 | 4 | 3.41 | 147 | 0.87 | 0.02 |
| 21 | 1 | 3.51 | 2490 | 0.89 | 0.29 |
| 21 | 2 | 3.45 | 361 | 0.87 | 0.04 |
| 21 | 3 | 3.44 | 221 | 0.97 | 0.03 |
| 21 | 4 | 3.45 | 151 | 0.94 | 0.02 |
| 22 | 1 | 3.54 | 2447 | 0.87 | 0.29 |
| 22 | 2 | 3.49 | 328 | 0.85 | 0.04 |
| 22 | 3 | 3.52 | 202 | 0.89 | 0.02 |
| 22 | 4 | 3.47 | 154 | 0.94 | 0.02 |
| 23 | 1 | 3.53 | 2419 | 0.87 | 0.28 |
| 23 | 2 | 3.47 | 303 | 0.89 | 0.04 |
| 23 | 3 | 3.39 | 207 | 0.89 | 0.02 |
| 23 | 4 | 3.57 | 145 | 0.93 | 0.02 |
| 24 | 1 | 3.55 | 2336 | 0.88 | 0.27 |
| 24 | 2 | 3.44 | 316 | 0.91 | 0.04 |
| 24 | 3 | 3.41 | 202 | 0.84 | 0.02 |
| 24 | 4 | 3.49 | 133 | 0.95 | 0.02 |
| 25 | 1 | 3.56 | 2276 | 0.87 | 0.27 |
| 25 | 2 | 3.56 | 310 | 0.90 | 0.04 |
| 25 | 3 | 3.47 | 210 | 0.90 | 0.02 |
| 25 | 4 | 3.44 | 127 | 0.87 | 0.01 |
| 26 | 1 | 3.56 | 2255 | 0.89 | 0.26 |
| 26 | 2 | 3.52 | 282 | 0.97 | 0.03 |
| 26 | 3 | 3.45 | 174 | 0.87 | 0.02 |
| 26 | 4 | 3.48 | 122 | 0.81 | 0.01 |
| 27 | 1 | 3.55 | 2188 | 0.90 | 0.26 |
| 27 | 2 | 3.41 | 288 | 0.87 | 0.03 |
| 27 | 3 | 3.41 | 180 | 0.88 | 0.02 |
| 27 | 4 | 3.42 | 123 | 0.89 | 0.01 |
| 28 | 1 | 3.56 | 2115 | 0.87 | 0.25 |
| 28 | 2 | 3.56 | 292 | 0.85 | 0.03 |
| 28 | 3 | 3.37 | 169 | 0.94 | 0.02 |
| 28 | 4 | 3.47 | 109 | 0.95 | 0.01 |
| 29 | 1 | 3.55 | 2111 | 0.88 | 0.25 |
| 29 | 2 | 3.43 | 262 | 0.89 | 0.03 |
| 29 | 3 | 3.42 | 168 | 0.98 | 0.02 |
| 29 | 4 | 3.46 | 99 | 0.93 | 0.01 |
| 30 | 1 | 3.58 | 2013 | 0.88 | 0.23 |
| 30 | 2 | 3.51 | 273 | 0.91 | 0.03 |
| 30 | 3 | 3.49 | 176 | 0.97 | 0.02 |
| 30 | 4 | 3.45 | 99 | 0.77 | 0.01 |
| 31 | 1 | 3.58 | 2031 | 0.87 | 0.24 |
| 31 | 2 | 3.50 | 245 | 0.90 | 0.03 |
| 31 | 3 | 3.43 | 160 | 0.89 | 0.02 |
| 31 | 4 | 3.46 | 99 | 0.80 | 0.01 |
| 32 | 1 | 3.58 | 1954 | 0.85 | 0.23 |
| 32 | 2 | 3.50 | 246 | 0.92 | 0.03 |
| 32 | 3 | 3.43 | 173 | 0.87 | 0.02 |
| 32 | 4 | 3.49 | 98 | 0.83 | 0.01 |
| 33 | 1 | 3.60 | 1944 | 0.85 | 0.23 |
| 33 | 2 | 3.51 | 238 | 0.85 | 0.03 |
| 33 | 3 | 3.41 | 166 | 0.95 | 0.02 |
| 33 | 4 | 3.40 | 93 | 0.98 | 0.01 |
| 34 | 1 | 3.59 | 1914 | 0.88 | 0.22 |
| 34 | 2 | 3.54 | 241 | 0.83 | 0.03 |
| 34 | 3 | 3.41 | 160 | 0.89 | 0.02 |
| 34 | 4 | 3.39 | 90 | 0.98 | 0.01 |
| 35 | 1 | 3.57 | 1855 | 0.86 | 0.22 |
| 35 | 2 | 3.54 | 251 | 0.89 | 0.03 |
| 35 | 3 | 3.55 | 137 | 0.91 | 0.02 |
| 35 | 4 | 3.43 | 92 | 0.94 | 0.01 |
| 36 | 1 | 3.59 | 1822 | 0.87 | 0.21 |
| 36 | 2 | 3.59 | 251 | 0.87 | 0.03 |
| 36 | 3 | 3.60 | 151 | 0.88 | 0.02 |
| 36 | 4 | 3.53 | 94 | 0.92 | 0.01 |
| 37 | 1 | 3.61 | 1789 | 0.87 | 0.21 |
| 37 | 2 | 3.55 | 231 | 0.84 | 0.03 |
| 37 | 3 | 3.45 | 116 | 0.90 | 0.01 |
| 37 | 4 | 3.53 | 81 | 0.85 | 0.01 |
| 38 | 1 | 3.61 | 1749 | 0.86 | 0.20 |
| 38 | 2 | 3.51 | 228 | 0.87 | 0.03 |
| 38 | 3 | 3.33 | 112 | 1.03 | 0.01 |
| 38 | 4 | 3.54 | 67 | 0.89 | 0.01 |
| 39 | 1 | 3.59 | 1709 | 0.89 | 0.20 |
| 39 | 2 | 3.47 | 213 | 0.94 | 0.02 |
| 39 | 3 | 3.39 | 110 | 0.95 | 0.01 |
| 39 | 4 | 3.60 | 75 | 0.84 | 0.01 |
| 40 | 1 | 3.61 | 1698 | 0.85 | 0.20 |
| 40 | 2 | 3.51 | 201 | 0.95 | 0.02 |
| 40 | 3 | 3.42 | 113 | 0.93 | 0.01 |
| 40 | 4 | 3.57 | 86 | 0.91 | 0.01 |
| 41 | 1 | 3.63 | 1699 | 0.83 | 0.20 |
| 41 | 2 | 3.61 | 194 | 0.89 | 0.02 |
| 41 | 3 | 3.52 | 121 | 0.90 | 0.01 |
| 41 | 4 | 3.42 | 71 | 0.90 | 0.01 |
| 42 | 1 | 3.63 | 1658 | 0.86 | 0.19 |
| 42 | 2 | 3.64 | 201 | 0.91 | 0.02 |
| 42 | 3 | 3.60 | 105 | 0.88 | 0.01 |
| 42 | 4 | 3.46 | 61 | 0.91 | 0.01 |
| 43 | 1 | 3.60 | 1648 | 0.87 | 0.19 |
| 43 | 2 | 3.65 | 200 | 0.80 | 0.02 |
| 43 | 3 | 3.55 | 99 | 0.87 | 0.01 |
| 43 | 4 | 3.59 | 64 | 0.97 | 0.01 |
| 44 | 1 | 3.65 | 1590 | 0.83 | 0.19 |
| 44 | 2 | 3.50 | 180 | 0.89 | 0.02 |
| 44 | 3 | 3.52 | 98 | 0.83 | 0.01 |
| 44 | 4 | 3.34 | 76 | 1.07 | 0.01 |
| 45 | 1 | 3.61 | 1585 | 0.86 | 0.19 |
| 45 | 2 | 3.56 | 191 | 0.87 | 0.02 |
| 45 | 3 | 3.56 | 98 | 0.86 | 0.01 |
| 45 | 4 | 3.63 | 59 | 0.89 | 0.01 |
| 46 | 1 | 3.61 | 1511 | 0.88 | 0.18 |
| 46 | 2 | 3.49 | 185 | 0.89 | 0.02 |
| 46 | 3 | 3.55 | 94 | 0.86 | 0.01 |
| 46 | 4 | 3.49 | 74 | 0.95 | 0.01 |
| 47 | 1 | 3.62 | 1483 | 0.84 | 0.17 |
| 47 | 2 | 3.39 | 168 | 0.89 | 0.02 |
| 47 | 3 | 3.61 | 82 | 0.91 | 0.01 |
| 47 | 4 | 3.57 | 68 | 0.82 | 0.01 |
| 48 | 1 | 3.61 | 1482 | 0.84 | 0.17 |
| 48 | 2 | 3.54 | 155 | 0.88 | 0.02 |
| 48 | 3 | 3.54 | 92 | 0.99 | 0.01 |
| 48 | 4 | 3.64 | 75 | 0.82 | 0.01 |
| 49 | 1 | 3.65 | 1440 | 0.84 | 0.17 |
| 49 | 2 | 3.49 | 148 | 0.92 | 0.02 |
| 49 | 3 | 3.60 | 92 | 0.90 | 0.01 |
| 49 | 4 | 3.53 | 75 | 0.86 | 0.01 |
| 50 | 1 | 3.67 | 1427 | 0.84 | 0.17 |
| 50 | 2 | 3.43 | 148 | 0.96 | 0.02 |
| 50 | 3 | 3.57 | 87 | 0.82 | 0.01 |
| 50 | 4 | 3.45 | 64 | 0.94 | 0.01 |
| 51 | 1 | 3.68 | 1400 | 0.84 | 0.16 |
| 51 | 2 | 3.49 | 159 | 0.85 | 0.02 |
| 51 | 3 | 3.64 | 86 | 0.85 | 0.01 |
| 51 | 4 | 3.47 | 60 | 0.83 | 0.01 |
| 52 | 1 | 3.68 | 1377 | 0.86 | 0.16 |
| 52 | 2 | 3.50 | 149 | 0.96 | 0.02 |
| 52 | 3 | 3.66 | 92 | 0.93 | 0.01 |
| 52 | 4 | 3.50 | 52 | 0.87 | 0.01 |
| 53 | 1 | 3.66 | 1369 | 0.85 | 0.16 |
| 53 | 2 | 3.55 | 138 | 0.91 | 0.02 |
| 53 | 3 | 3.64 | 107 | 0.95 | 0.01 |
| 53 | 4 | 3.57 | 42 | 0.94 | 0.00 |
| 54 | 1 | 3.64 | 1328 | 0.86 | 0.16 |
| 54 | 2 | 3.55 | 137 | 0.89 | 0.02 |
| 54 | 3 | 3.53 | 102 | 0.92 | 0.01 |
| 54 | 4 | 3.38 | 53 | 0.88 | 0.01 |
| 55 | 1 | 3.67 | 1300 | 0.85 | 0.15 |
| 55 | 2 | 3.56 | 131 | 0.86 | 0.02 |
| 55 | 3 | 3.51 | 89 | 0.79 | 0.01 |
| 55 | 4 | 3.73 | 49 | 0.84 | 0.01 |
| 56 | 1 | 3.67 | 1285 | 0.87 | 0.15 |
| 56 | 2 | 3.57 | 134 | 0.84 | 0.02 |
| 56 | 3 | 3.43 | 84 | 0.96 | 0.01 |
| 56 | 4 | 3.61 | 41 | 0.83 | 0.00 |
| 57 | 1 | 3.68 | 1253 | 0.84 | 0.15 |
| 57 | 2 | 3.46 | 117 | 0.92 | 0.01 |
| 57 | 3 | 3.44 | 73 | 0.93 | 0.01 |
| 57 | 4 | 3.23 | 48 | 0.97 | 0.01 |
| 58 | 1 | 3.65 | 1271 | 0.88 | 0.15 |
| 58 | 2 | 3.41 | 115 | 0.90 | 0.01 |
| 58 | 3 | 3.54 | 83 | 0.93 | 0.01 |
| 58 | 4 | 3.51 | 49 | 0.89 | 0.01 |
| 59 | 1 | 3.64 | 1218 | 0.86 | 0.14 |
| 59 | 2 | 3.57 | 121 | 0.85 | 0.01 |
| 59 | 3 | 3.54 | 72 | 1.01 | 0.01 |
| 59 | 4 | 3.60 | 48 | 0.82 | 0.01 |
| 60 | 1 | 3.68 | 1233 | 0.87 | 0.14 |
| 60 | 2 | 3.58 | 127 | 0.78 | 0.01 |
| 60 | 3 | 3.51 | 69 | 0.96 | 0.01 |
| 60 | 4 | 3.46 | 46 | 0.98 | 0.01 |
| 61 | 1 | 3.65 | 1164 | 0.87 | 0.14 |
| 61 | 2 | 3.55 | 123 | 0.89 | 0.01 |
| 61 | 3 | 3.75 | 65 | 0.79 | 0.01 |
| 61 | 4 | 3.40 | 43 | 1.18 | 0.01 |
| 62 | 1 | 3.71 | 1170 | 0.82 | 0.14 |
| 62 | 2 | 3.48 | 116 | 0.91 | 0.01 |
| 62 | 3 | 3.49 | 74 | 0.88 | 0.01 |
| 62 | 4 | 3.50 | 44 | 1.07 | 0.01 |
| 63 | 1 | 3.66 | 1131 | 0.85 | 0.13 |
| 63 | 2 | 3.59 | 125 | 0.83 | 0.01 |
| 63 | 3 | 3.50 | 72 | 0.93 | 0.01 |
| 63 | 4 | 3.59 | 46 | 0.83 | 0.01 |
| 64 | 1 | 3.64 | 1134 | 0.82 | 0.13 |
| 64 | 2 | 3.56 | 119 | 0.85 | 0.01 |
| 64 | 3 | 3.59 | 74 | 0.87 | 0.01 |
| 64 | 4 | 3.74 | 38 | 0.89 | 0.00 |
| 65 | 1 | 3.67 | 1144 | 0.82 | 0.13 |
| 65 | 2 | 3.57 | 109 | 0.79 | 0.01 |
| 65 | 3 | 3.51 | 68 | 0.84 | 0.01 |
| 65 | 4 | 3.50 | 32 | 0.92 | 0.00 |
| 66 | 1 | 3.67 | 1111 | 0.83 | 0.13 |
| 66 | 2 | 3.64 | 105 | 0.82 | 0.01 |
| 66 | 3 | 3.58 | 65 | 0.83 | 0.01 |
| 66 | 4 | 3.54 | 37 | 0.73 | 0.00 |
| 67 | 1 | 3.65 | 1098 | 0.86 | 0.13 |
| 67 | 2 | 3.65 | 101 | 0.84 | 0.01 |
| 67 | 3 | 3.59 | 70 | 0.88 | 0.01 |
| 67 | 4 | 3.33 | 33 | 0.74 | 0.00 |
| 68 | 1 | 3.66 | 1079 | 0.83 | 0.13 |
| 68 | 2 | 3.51 | 101 | 1.01 | 0.01 |
| 68 | 3 | 3.70 | 60 | 0.81 | 0.01 |
| 68 | 4 | 3.36 | 33 | 0.82 | 0.00 |
| 69 | 1 | 3.66 | 1046 | 0.85 | 0.12 |
| 69 | 2 | 3.56 | 100 | 0.94 | 0.01 |
| 69 | 3 | 3.72 | 57 | 0.92 | 0.01 |
| 69 | 4 | 3.49 | 35 | 0.61 | 0.00 |
| 70 | 1 | 3.68 | 1044 | 0.86 | 0.12 |
| 70 | 2 | 3.55 | 107 | 0.78 | 0.01 |
| 70 | 3 | 3.53 | 60 | 0.85 | 0.01 |
| 70 | 4 | 3.40 | 30 | 0.81 | 0.00 |
| 71 | 1 | 3.68 | 991 | 0.85 | 0.12 |
| 71 | 2 | 3.53 | 101 | 0.64 | 0.01 |
| 71 | 3 | 3.60 | 52 | 1.03 | 0.01 |
| 71 | 4 | 3.32 | 31 | 0.75 | 0.00 |
| 72 | 1 | 3.70 | 962 | 0.82 | 0.11 |
| 72 | 2 | 3.61 | 102 | 0.89 | 0.01 |
| 72 | 3 | 3.58 | 60 | 0.91 | 0.01 |
| 72 | 4 | 3.33 | 33 | 0.74 | 0.00 |
| 73 | 1 | 3.67 | 972 | 0.86 | 0.11 |
| 73 | 2 | 3.58 | 95 | 0.82 | 0.01 |
| 73 | 3 | 3.37 | 59 | 0.85 | 0.01 |
| 73 | 4 | 3.56 | 34 | 0.82 | 0.00 |
| 74 | 1 | 3.67 | 975 | 0.83 | 0.11 |
| 74 | 2 | 3.66 | 102 | 0.90 | 0.01 |
| 74 | 3 | 3.35 | 57 | 1.09 | 0.01 |
| 74 | 4 | 3.32 | 38 | 0.96 | 0.00 |
| 75 | 1 | 3.69 | 982 | 0.88 | 0.11 |
| 75 | 2 | 3.69 | 86 | 0.86 | 0.01 |
| 75 | 3 | 3.47 | 49 | 1.06 | 0.01 |
| 75 | 4 | 3.53 | 30 | 0.90 | 0.00 |
| 76 | 1 | 3.70 | 936 | 0.84 | 0.11 |
| 76 | 2 | 3.61 | 92 | 0.89 | 0.01 |
| 76 | 3 | 3.42 | 53 | 0.82 | 0.01 |
| 76 | 4 | 3.57 | 30 | 0.63 | 0.00 |
| 77 | 1 | 3.70 | 951 | 0.82 | 0.11 |
| 77 | 2 | 3.71 | 85 | 0.81 | 0.01 |
| 77 | 3 | 3.48 | 56 | 1.03 | 0.01 |
| 77 | 4 | 3.54 | 28 | 0.79 | 0.00 |
| 78 | 1 | 3.69 | 956 | 0.84 | 0.11 |
| 78 | 2 | 3.71 | 70 | 0.84 | 0.01 |
| 78 | 3 | 3.48 | 44 | 0.73 | 0.01 |
| 78 | 4 | 3.41 | 29 | 0.68 | 0.00 |
| 79 | 1 | 3.69 | 939 | 0.85 | 0.11 |
| 79 | 2 | 3.61 | 72 | 0.96 | 0.01 |
| 79 | 3 | 3.52 | 46 | 0.84 | 0.01 |
| 79 | 4 | 3.28 | 25 | 0.89 | 0.00 |
| 80 | 1 | 3.67 | 883 | 0.87 | 0.10 |
| 80 | 2 | 3.69 | 81 | 0.88 | 0.01 |
| 80 | 3 | 3.35 | 40 | 0.86 | 0.00 |
| 80 | 4 | 3.41 | 22 | 1.18 | 0.00 |
| 81 | 1 | 3.70 | 925 | 0.87 | 0.11 |
| 81 | 2 | 3.57 | 82 | 0.90 | 0.01 |
| 81 | 3 | 3.60 | 43 | 0.90 | 0.01 |
| 81 | 4 | 3.12 | 17 | 0.93 | 0.00 |
| 82 | 1 | 3.64 | 908 | 0.87 | 0.11 |
| 82 | 2 | 3.54 | 68 | 0.97 | 0.01 |
| 82 | 3 | 3.58 | 40 | 0.75 | 0.00 |
| 82 | 4 | 3.25 | 24 | 0.61 | 0.00 |
| 83 | 1 | 3.66 | 889 | 0.90 | 0.10 |
| 83 | 2 | 3.61 | 62 | 0.88 | 0.01 |
| 83 | 3 | 3.62 | 47 | 0.74 | 0.01 |
| 83 | 4 | 3.36 | 14 | 0.74 | 0.00 |
| 84 | 1 | 3.71 | 847 | 0.83 | 0.10 |
| 84 | 2 | 3.56 | 71 | 0.92 | 0.01 |
| 84 | 3 | 3.65 | 46 | 0.71 | 0.01 |
| 84 | 4 | 3.00 | 14 | 1.18 | 0.00 |
| 85 | 1 | 3.70 | 865 | 0.84 | 0.10 |
| 85 | 2 | 3.65 | 71 | 0.93 | 0.01 |
| 85 | 3 | 3.54 | 41 | 0.98 | 0.00 |
| 85 | 4 | 3.35 | 23 | 0.71 | 0.00 |
| 86 | 1 | 3.71 | 854 | 0.83 | 0.10 |
| 86 | 2 | 3.62 | 68 | 0.83 | 0.01 |
| 86 | 3 | 3.53 | 30 | 0.68 | 0.00 |
| 86 | 4 | 3.20 | 25 | 1.04 | 0.00 |
| 87 | 1 | 3.70 | 842 | 0.84 | 0.10 |
| 87 | 2 | 3.56 | 72 | 0.90 | 0.01 |
| 87 | 3 | 3.62 | 42 | 0.79 | 0.00 |
| 87 | 4 | 3.55 | 20 | 1.10 | 0.00 |
| 88 | 1 | 3.69 | 803 | 0.84 | 0.09 |
| 88 | 2 | 3.69 | 74 | 0.76 | 0.01 |
| 88 | 3 | 3.49 | 45 | 0.89 | 0.01 |
| 88 | 4 | 3.50 | 22 | 1.06 | 0.00 |
| 89 | 1 | 3.73 | 792 | 0.82 | 0.09 |
| 89 | 2 | 3.76 | 70 | 0.81 | 0.01 |
| 89 | 3 | 3.53 | 47 | 0.97 | 0.01 |
| 89 | 4 | 3.80 | 20 | 0.83 | 0.00 |
| 90 | 1 | 3.74 | 806 | 0.85 | 0.09 |
| 90 | 2 | 3.67 | 58 | 0.85 | 0.01 |
| 90 | 3 | 3.47 | 36 | 1.03 | 0.00 |
| 90 | 4 | 3.58 | 19 | 0.84 | 0.00 |

**Table S3.** Combinations and relative frequencies of each meditation session time point and session length factor.

| **Session number** | **Session length factor** | **Mean** | **N** | **SD** | **Alpha** |
| --- | --- | --- | --- | --- | --- |
| 1 | 5-10 | 3.35 | 5251 | 0.88 | 0.61 |
| 1 | 10-20 | 3.31 | 2610 | 0.89 | 0.30 |
| 1 | 20-30 | 3.35 | 705 | 0.94 | 0.08 |
| 2 | 5-10 | 3.41 | 4127 | 0.88 | 0.48 |
| 2 | 10-20 | 3.32 | 2383 | 0.89 | 0.28 |
| 2 | 20-30 | 3.28 | 565 | 0.93 | 0.07 |
| 3 | 5-10 | 3.42 | 3723 | 0.87 | 0.43 |
| 3 | 10-20 | 3.41 | 2145 | 0.89 | 0.25 |
| 3 | 20-30 | 3.27 | 548 | 0.96 | 0.06 |
| 4 | 5-10 | 3.45 | 3408 | 0.88 | 0.40 |
| 4 | 10-20 | 3.36 | 2086 | 0.92 | 0.24 |
| 4 | 20-30 | 3.33 | 533 | 0.87 | 0.06 |
| 5 | 5-10 | 3.43 | 3252 | 0.88 | 0.38 |
| 5 | 10-20 | 3.41 | 1990 | 0.90 | 0.23 |
| 5 | 20-30 | 3.30 | 464 | 0.92 | 0.05 |
| 6 | 5-10 | 3.44 | 3089 | 0.87 | 0.36 |
| 6 | 10-20 | 3.42 | 1877 | 0.85 | 0.22 |
| 6 | 20-30 | 3.38 | 509 | 0.98 | 0.06 |
| 7 | 5-10 | 3.45 | 2914 | 0.86 | 0.34 |
| 7 | 10-20 | 3.44 | 1893 | 0.88 | 0.22 |
| 7 | 20-30 | 3.35 | 494 | 0.92 | 0.06 |
| 8 | 5-10 | 3.46 | 2752 | 0.89 | 0.32 |
| 8 | 10-20 | 3.45 | 1819 | 0.89 | 0.21 |
| 8 | 20-30 | 3.32 | 457 | 0.92 | 0.05 |
| 9 | 5-10 | 3.48 | 2696 | 0.87 | 0.31 |
| 9 | 10-20 | 3.40 | 1724 | 0.92 | 0.20 |
| 9 | 20-30 | 3.46 | 459 | 0.95 | 0.05 |
| 10 | 5-10 | 3.47 | 2503 | 0.86 | 0.29 |
| 10 | 10-20 | 3.44 | 1725 | 0.89 | 0.20 |
| 10 | 20-30 | 3.42 | 436 | 0.89 | 0.05 |
| 11 | 5-10 | 3.47 | 2412 | 0.88 | 0.28 |
| 11 | 10-20 | 3.44 | 1629 | 0.91 | 0.19 |
| 11 | 20-30 | 3.34 | 419 | 0.94 | 0.05 |
| 12 | 5-10 | 3.53 | 2300 | 0.86 | 0.27 |
| 12 | 10-20 | 3.42 | 1591 | 0.87 | 0.19 |
| 12 | 20-30 | 3.37 | 434 | 0.96 | 0.05 |
| 13 | 5-10 | 3.52 | 2210 | 0.86 | 0.26 |
| 13 | 10-20 | 3.40 | 1514 | 0.93 | 0.18 |
| 13 | 20-30 | 3.37 | 380 | 0.96 | 0.04 |
| 14 | 5-10 | 3.53 | 2101 | 0.86 | 0.25 |
| 14 | 10-20 | 3.43 | 1480 | 0.90 | 0.17 |
| 14 | 20-30 | 3.44 | 394 | 0.85 | 0.05 |
| 15 | 5-10 | 3.48 | 2076 | 0.90 | 0.24 |
| 15 | 10-20 | 3.46 | 1423 | 0.89 | 0.17 |
| 15 | 20-30 | 3.39 | 352 | 0.93 | 0.04 |
| 16 | 5-10 | 3.51 | 2048 | 0.85 | 0.24 |
| 16 | 10-20 | 3.44 | 1312 | 0.90 | 0.15 |
| 16 | 20-30 | 3.46 | 381 | 0.92 | 0.04 |
| 17 | 5-10 | 3.48 | 1951 | 0.90 | 0.23 |
| 17 | 10-20 | 3.46 | 1301 | 0.87 | 0.15 |
| 17 | 20-30 | 3.48 | 374 | 0.88 | 0.04 |
| 18 | 5-10 | 3.52 | 1863 | 0.86 | 0.22 |
| 18 | 10-20 | 3.50 | 1292 | 0.91 | 0.15 |
| 18 | 20-30 | 3.43 | 361 | 0.92 | 0.04 |
| 19 | 5-10 | 3.52 | 1799 | 0.85 | 0.21 |
| 19 | 10-20 | 3.48 | 1288 | 0.88 | 0.15 |
| 19 | 20-30 | 3.48 | 318 | 0.88 | 0.04 |
| 20 | 5-10 | 3.52 | 1790 | 0.87 | 0.21 |
| 20 | 10-20 | 3.44 | 1184 | 0.92 | 0.14 |
| 20 | 20-30 | 3.50 | 323 | 0.90 | 0.04 |
| 21 | 5-10 | 3.50 | 1797 | 0.88 | 0.21 |
| 21 | 10-20 | 3.50 | 1096 | 0.89 | 0.13 |
| 21 | 20-30 | 3.46 | 330 | 1.00 | 0.04 |
| 22 | 5-10 | 3.56 | 1699 | 0.85 | 0.20 |
| 22 | 10-20 | 3.50 | 1115 | 0.89 | 0.13 |
| 22 | 20-30 | 3.50 | 317 | 0.90 | 0.04 |
| 23 | 5-10 | 3.56 | 1644 | 0.85 | 0.19 |
| 23 | 10-20 | 3.48 | 1111 | 0.91 | 0.13 |
| 23 | 20-30 | 3.43 | 319 | 0.92 | 0.04 |
| 24 | 5-10 | 3.56 | 1570 | 0.87 | 0.18 |
| 24 | 10-20 | 3.48 | 1090 | 0.89 | 0.13 |
| 24 | 20-30 | 3.52 | 327 | 0.96 | 0.04 |
| 25 | 5-10 | 3.58 | 1577 | 0.87 | 0.18 |
| 25 | 10-20 | 3.50 | 1032 | 0.88 | 0.12 |
| 25 | 20-30 | 3.55 | 314 | 0.90 | 0.04 |
| 26 | 5-10 | 3.57 | 1487 | 0.88 | 0.17 |
| 26 | 10-20 | 3.51 | 1040 | 0.89 | 0.12 |
| 26 | 20-30 | 3.55 | 306 | 0.95 | 0.04 |
| 27 | 5-10 | 3.53 | 1499 | 0.90 | 0.17 |
| 27 | 10-20 | 3.48 | 977 | 0.90 | 0.11 |
| 27 | 20-30 | 3.55 | 303 | 0.89 | 0.04 |
| 28 | 5-10 | 3.56 | 1413 | 0.86 | 0.16 |
| 28 | 10-20 | 3.53 | 990 | 0.87 | 0.12 |
| 28 | 20-30 | 3.51 | 282 | 0.95 | 0.03 |
| 29 | 5-10 | 3.55 | 1352 | 0.87 | 0.16 |
| 29 | 10-20 | 3.50 | 993 | 0.91 | 0.12 |
| 29 | 20-30 | 3.53 | 295 | 0.96 | 0.03 |
| 30 | 5-10 | 3.58 | 1313 | 0.86 | 0.15 |
| 30 | 10-20 | 3.51 | 948 | 0.90 | 0.11 |
| 30 | 20-30 | 3.60 | 300 | 0.95 | 0.04 |
| 31 | 5-10 | 3.57 | 1349 | 0.87 | 0.16 |
| 31 | 10-20 | 3.57 | 893 | 0.87 | 0.10 |
| 31 | 20-30 | 3.46 | 293 | 0.92 | 0.03 |
| 32 | 5-10 | 3.55 | 1340 | 0.85 | 0.16 |
| 32 | 10-20 | 3.57 | 866 | 0.87 | 0.10 |
| 32 | 20-30 | 3.53 | 265 | 0.86 | 0.03 |
| 33 | 5-10 | 3.59 | 1268 | 0.86 | 0.15 |
| 33 | 10-20 | 3.55 | 910 | 0.85 | 0.11 |
| 33 | 20-30 | 3.57 | 263 | 0.89 | 0.03 |
| 34 | 5-10 | 3.59 | 1285 | 0.88 | 0.15 |
| 34 | 10-20 | 3.55 | 855 | 0.87 | 0.10 |
| 34 | 20-30 | 3.49 | 265 | 0.93 | 0.03 |
| 35 | 5-10 | 3.58 | 1226 | 0.85 | 0.14 |
| 35 | 10-20 | 3.54 | 862 | 0.90 | 0.10 |
| 35 | 20-30 | 3.51 | 247 | 0.85 | 0.03 |
| 36 | 5-10 | 3.62 | 1222 | 0.87 | 0.14 |
| 36 | 10-20 | 3.53 | 808 | 0.86 | 0.09 |
| 36 | 20-30 | 3.66 | 288 | 0.88 | 0.03 |
| 37 | 5-10 | 3.60 | 1156 | 0.88 | 0.13 |
| 37 | 10-20 | 3.60 | 811 | 0.86 | 0.09 |
| 37 | 20-30 | 3.53 | 250 | 0.86 | 0.03 |
| 38 | 5-10 | 3.62 | 1137 | 0.83 | 0.13 |
| 38 | 10-20 | 3.55 | 793 | 0.91 | 0.09 |
| 38 | 20-30 | 3.51 | 226 | 0.93 | 0.03 |
| 39 | 5-10 | 3.61 | 1145 | 0.85 | 0.13 |
| 39 | 10-20 | 3.53 | 728 | 0.95 | 0.08 |
| 39 | 20-30 | 3.50 | 234 | 0.96 | 0.03 |
| 40 | 5-10 | 3.59 | 1111 | 0.89 | 0.13 |
| 40 | 10-20 | 3.58 | 759 | 0.84 | 0.09 |
| 40 | 20-30 | 3.61 | 228 | 0.84 | 0.03 |
| 41 | 5-10 | 3.66 | 1134 | 0.83 | 0.13 |
| 41 | 10-20 | 3.54 | 720 | 0.85 | 0.08 |
| 41 | 20-30 | 3.61 | 231 | 0.88 | 0.03 |
| 42 | 5-10 | 3.64 | 1047 | 0.84 | 0.12 |
| 42 | 10-20 | 3.59 | 758 | 0.87 | 0.09 |
| 42 | 20-30 | 3.64 | 220 | 0.92 | 0.03 |
| 43 | 5-10 | 3.64 | 1050 | 0.84 | 0.12 |
| 43 | 10-20 | 3.57 | 737 | 0.86 | 0.09 |
| 43 | 20-30 | 3.56 | 224 | 0.98 | 0.03 |
| 44 | 5-10 | 3.65 | 1039 | 0.82 | 0.12 |
| 44 | 10-20 | 3.55 | 675 | 0.88 | 0.08 |
| 44 | 20-30 | 3.64 | 230 | 0.86 | 0.03 |
| 45 | 5-10 | 3.61 | 1034 | 0.84 | 0.12 |
| 45 | 10-20 | 3.59 | 704 | 0.88 | 0.08 |
| 45 | 20-30 | 3.63 | 195 | 0.88 | 0.02 |
| 46 | 5-10 | 3.61 | 986 | 0.87 | 0.12 |
| 46 | 10-20 | 3.55 | 658 | 0.89 | 0.08 |
| 46 | 20-30 | 3.65 | 220 | 0.91 | 0.03 |
| 47 | 5-10 | 3.62 | 940 | 0.80 | 0.11 |
| 47 | 10-20 | 3.57 | 652 | 0.89 | 0.08 |
| 47 | 20-30 | 3.60 | 209 | 0.93 | 0.02 |
| 48 | 5-10 | 3.64 | 946 | 0.84 | 0.11 |
| 48 | 10-20 | 3.56 | 655 | 0.86 | 0.08 |
| 48 | 20-30 | 3.56 | 203 | 0.83 | 0.02 |
| 49 | 5-10 | 3.64 | 903 | 0.86 | 0.11 |
| 49 | 10-20 | 3.63 | 654 | 0.84 | 0.08 |
| 49 | 20-30 | 3.55 | 198 | 0.86 | 0.02 |
| 50 | 5-10 | 3.68 | 935 | 0.85 | 0.11 |
| 50 | 10-20 | 3.57 | 625 | 0.87 | 0.07 |
| 50 | 20-30 | 3.64 | 166 | 0.85 | 0.02 |
| 51 | 5-10 | 3.71 | 910 | 0.80 | 0.11 |
| 51 | 10-20 | 3.57 | 604 | 0.88 | 0.07 |
| 51 | 20-30 | 3.61 | 191 | 0.90 | 0.02 |
| 52 | 5-10 | 3.66 | 872 | 0.87 | 0.10 |
| 52 | 10-20 | 3.64 | 602 | 0.88 | 0.07 |
| 52 | 20-30 | 3.67 | 196 | 0.87 | 0.02 |
| 53 | 5-10 | 3.68 | 864 | 0.84 | 0.10 |
| 53 | 10-20 | 3.57 | 606 | 0.90 | 0.07 |
| 53 | 20-30 | 3.73 | 186 | 0.87 | 0.02 |
| 54 | 5-10 | 3.64 | 848 | 0.85 | 0.10 |
| 54 | 10-20 | 3.55 | 594 | 0.88 | 0.07 |
| 54 | 20-30 | 3.72 | 178 | 0.89 | 0.02 |
| 55 | 5-10 | 3.68 | 805 | 0.82 | 0.09 |
| 55 | 10-20 | 3.64 | 569 | 0.86 | 0.07 |
| 55 | 20-30 | 3.62 | 195 | 0.94 | 0.02 |
| 56 | 5-10 | 3.68 | 803 | 0.87 | 0.09 |
| 56 | 10-20 | 3.62 | 560 | 0.87 | 0.07 |
| 56 | 20-30 | 3.58 | 181 | 0.91 | 0.02 |
| 57 | 5-10 | 3.67 | 793 | 0.82 | 0.09 |
| 57 | 10-20 | 3.62 | 542 | 0.91 | 0.06 |
| 57 | 20-30 | 3.56 | 156 | 0.85 | 0.02 |
| 58 | 5-10 | 3.62 | 773 | 0.86 | 0.09 |
| 58 | 10-20 | 3.61 | 573 | 0.90 | 0.07 |
| 58 | 20-30 | 3.63 | 172 | 0.94 | 0.02 |
| 59 | 5-10 | 3.68 | 737 | 0.84 | 0.09 |
| 59 | 10-20 | 3.57 | 554 | 0.87 | 0.06 |
| 59 | 20-30 | 3.62 | 168 | 0.93 | 0.02 |
| 60 | 5-10 | 3.69 | 769 | 0.85 | 0.09 |
| 60 | 10-20 | 3.66 | 532 | 0.87 | 0.06 |
| 60 | 20-30 | 3.49 | 174 | 0.96 | 0.02 |
| 61 | 5-10 | 3.68 | 751 | 0.88 | 0.09 |
| 61 | 10-20 | 3.60 | 484 | 0.87 | 0.06 |
| 61 | 20-30 | 3.61 | 160 | 0.91 | 0.02 |
| 62 | 5-10 | 3.71 | 733 | 0.85 | 0.09 |
| 62 | 10-20 | 3.63 | 512 | 0.82 | 0.06 |
| 62 | 20-30 | 3.64 | 159 | 0.91 | 0.02 |
| 63 | 5-10 | 3.68 | 703 | 0.84 | 0.08 |
| 63 | 10-20 | 3.61 | 503 | 0.85 | 0.06 |
| 63 | 20-30 | 3.57 | 168 | 0.90 | 0.02 |
| 64 | 5-10 | 3.63 | 701 | 0.85 | 0.08 |
| 64 | 10-20 | 3.65 | 510 | 0.83 | 0.06 |
| 64 | 20-30 | 3.56 | 154 | 0.78 | 0.02 |
| 65 | 5-10 | 3.67 | 692 | 0.83 | 0.08 |
| 65 | 10-20 | 3.63 | 501 | 0.82 | 0.06 |
| 65 | 20-30 | 3.60 | 160 | 0.82 | 0.02 |
| 66 | 5-10 | 3.68 | 696 | 0.84 | 0.08 |
| 66 | 10-20 | 3.68 | 453 | 0.81 | 0.05 |
| 66 | 20-30 | 3.53 | 169 | 0.82 | 0.02 |
| 67 | 5-10 | 3.69 | 691 | 0.85 | 0.08 |
| 67 | 10-20 | 3.58 | 450 | 0.87 | 0.05 |
| 67 | 20-30 | 3.60 | 161 | 0.85 | 0.02 |
| 68 | 5-10 | 3.66 | 683 | 0.84 | 0.08 |
| 68 | 10-20 | 3.65 | 440 | 0.83 | 0.05 |
| 68 | 20-30 | 3.53 | 150 | 0.91 | 0.02 |
| 69 | 5-10 | 3.69 | 636 | 0.85 | 0.07 |
| 69 | 10-20 | 3.61 | 445 | 0.86 | 0.05 |
| 69 | 20-30 | 3.58 | 157 | 0.83 | 0.02 |
| 70 | 5-10 | 3.70 | 640 | 0.84 | 0.07 |
| 70 | 10-20 | 3.62 | 445 | 0.82 | 0.05 |
| 70 | 20-30 | 3.60 | 156 | 0.95 | 0.02 |
| 71 | 5-10 | 3.70 | 605 | 0.82 | 0.07 |
| 71 | 10-20 | 3.63 | 432 | 0.87 | 0.05 |
| 71 | 20-30 | 3.56 | 138 | 0.88 | 0.02 |
| 72 | 5-10 | 3.69 | 602 | 0.84 | 0.07 |
| 72 | 10-20 | 3.67 | 424 | 0.84 | 0.05 |
| 72 | 20-30 | 3.66 | 131 | 0.81 | 0.02 |
| 73 | 5-10 | 3.70 | 633 | 0.83 | 0.07 |
| 73 | 10-20 | 3.58 | 404 | 0.86 | 0.05 |
| 73 | 20-30 | 3.59 | 123 | 0.96 | 0.01 |
| 74 | 5-10 | 3.66 | 593 | 0.85 | 0.07 |
| 74 | 10-20 | 3.62 | 441 | 0.87 | 0.05 |
| 74 | 20-30 | 3.63 | 138 | 0.89 | 0.02 |
| 75 | 5-10 | 3.70 | 623 | 0.89 | 0.07 |
| 75 | 10-20 | 3.62 | 393 | 0.91 | 0.05 |
| 75 | 20-30 | 3.69 | 131 | 0.79 | 0.02 |
| 76 | 5-10 | 3.71 | 593 | 0.84 | 0.07 |
| 76 | 10-20 | 3.67 | 388 | 0.82 | 0.05 |
| 76 | 20-30 | 3.50 | 130 | 0.90 | 0.02 |
| 77 | 5-10 | 3.72 | 624 | 0.81 | 0.07 |
| 77 | 10-20 | 3.70 | 372 | 0.84 | 0.04 |
| 77 | 20-30 | 3.49 | 124 | 0.88 | 0.01 |
| 78 | 5-10 | 3.68 | 576 | 0.81 | 0.07 |
| 78 | 10-20 | 3.68 | 414 | 0.83 | 0.05 |
| 78 | 20-30 | 3.65 | 109 | 0.97 | 0.01 |
| 79 | 5-10 | 3.69 | 565 | 0.86 | 0.07 |
| 79 | 10-20 | 3.65 | 396 | 0.87 | 0.05 |
| 79 | 20-30 | 3.64 | 121 | 0.85 | 0.01 |
| 80 | 5-10 | 3.67 | 559 | 0.89 | 0.07 |
| 80 | 10-20 | 3.61 | 353 | 0.89 | 0.04 |
| 80 | 20-30 | 3.70 | 114 | 0.83 | 0.01 |
| 81 | 5-10 | 3.70 | 573 | 0.86 | 0.07 |
| 81 | 10-20 | 3.64 | 369 | 0.93 | 0.04 |
| 81 | 20-30 | 3.67 | 125 | 0.79 | 0.01 |
| 82 | 5-10 | 3.63 | 570 | 0.85 | 0.07 |
| 82 | 10-20 | 3.59 | 353 | 0.88 | 0.04 |
| 82 | 20-30 | 3.70 | 117 | 0.89 | 0.01 |
| 83 | 5-10 | 3.68 | 521 | 0.86 | 0.06 |
| 83 | 10-20 | 3.66 | 361 | 0.85 | 0.04 |
| 83 | 20-30 | 3.53 | 130 | 1.07 | 0.02 |
| 84 | 5-10 | 3.69 | 517 | 0.81 | 0.06 |
| 84 | 10-20 | 3.72 | 351 | 0.85 | 0.04 |
| 84 | 20-30 | 3.59 | 110 | 0.96 | 0.01 |
| 85 | 5-10 | 3.70 | 515 | 0.82 | 0.06 |
| 85 | 10-20 | 3.63 | 367 | 0.90 | 0.04 |
| 85 | 20-30 | 3.75 | 118 | 0.80 | 0.01 |
| 86 | 5-10 | 3.71 | 519 | 0.81 | 0.06 |
| 86 | 10-20 | 3.68 | 350 | 0.85 | 0.04 |
| 86 | 20-30 | 3.63 | 108 | 0.89 | 0.01 |
| 87 | 5-10 | 3.66 | 529 | 0.81 | 0.06 |
| 87 | 10-20 | 3.68 | 327 | 0.88 | 0.04 |
| 87 | 20-30 | 3.78 | 120 | 0.90 | 0.01 |
| 88 | 5-10 | 3.69 | 493 | 0.83 | 0.06 |
| 88 | 10-20 | 3.71 | 339 | 0.87 | 0.04 |
| 88 | 20-30 | 3.55 | 112 | 0.80 | 0.01 |
| 89 | 5-10 | 3.76 | 501 | 0.80 | 0.06 |
| 89 | 10-20 | 3.72 | 324 | 0.86 | 0.04 |
| 89 | 20-30 | 3.60 | 104 | 0.85 | 0.01 |
| 90 | 5-10 | 3.75 | 500 | 0.84 | 0.06 |
| 90 | 10-20 | 3.71 | 320 | 0.87 | 0.04 |
| 90 | 20-30 | 3.66 | 99 | 0.89 | 0.01 |

**Table S4.** Participant count and average mood (sd) by country.

| **Country** | **N** | **Mood score (mean)** | **Mood score (sd)** |
| --- | --- | --- | --- |
| Algeria | 1 | 3.07 | - |
| American Samoa | 2 | 2.72 | 1.02 |
| Angola | 1 | 3.8 | - |
| Anguilla | 2 | 3 | 0 |
| Argentina | 9 | 3.19 | 0.49 |
| Aruba | 1 | 4.03 | - |
| Australia | 838 | 3.38 | 0.65 |
| Austria | 18 | 3.49 | 0.52 |
| Bahamas | 1 | 3 | - |
| Bahrain | 1 | 3.62 | - |
| Belarus | 3 | 3.38 | 0.32 |
| Belgium | 40 | 3.24 | 0.53 |
| Bhutan | 1 | 3.47 | - |
| Brazil | 299 | 3.59 | 0.63 |
| Bulgaria | 5 | 3.69 | 0.52 |
| Canada | 745 | 3.35 | 0.65 |
| Chile | 6 | 3.57 | 0.76 |
| China | 8 | 3.78 | 0.63 |
| Colombia | 21 | 3.58 | 0.62 |
| Costa Rica | 7 | 3.45 | 0.26 |
| Croatia | 6 | 3.17 | 0.52 |
| Cyprus | 3 | 3.41 | 0.62 |
| Czech Republic | 11 | 3.3 | 0.45 |
| Denmark | 26 | 3.39 | 0.52 |
| Dominican Republic | 3 | 3.45 | 0.46 |
| Ecuador | 5 | 3.15 | 0.64 |
| Egypt | 4 | 3.53 | 0.21 |
| El Salvador | 1 | 4.33 | - |
| Estonia | 6 | 3.72 | 0.89 |
| Faroe Islands | 2 | 3.36 | 0.16 |
| Finland | 22 | 3.05 | 0.73 |
| France | 70 | 3.47 | 0.57 |
| Georgia | 1 | 3.5 | - |
| Germany | 152 | 3.49 | 0.52 |
| Ghana | 1 | 3.25 | - |
| Greece | 5 | 3.05 | 0.75 |
| Guadeloupe | 1 | 3.39 | - |
| Guatemala | 1 | 3.55 | - |
| Hong Kong | 21 | 3.34 | 0.65 |
| Hungary | 11 | 3.53 | 0.59 |
| Iceland | 3 | 3.38 | 0.33 |
| India | 110 | 3.34 | 0.8 |
| Indonesia | 11 | 3.65 | 0.7 |
| Iran, Islamic Republic of | 1 | 4.67 | - |
| Ireland | 117 | 3.28 | 0.67 |
| Israel | 22 | 3.06 | 0.93 |
| Italy | 45 | 3.21 | 0.59 |
| Japan | 13 | 3.33 | 0.72 |
| Kazakhstan | 1 | 3 | - |
| Kenya | 5 | 3.97 | 0.62 |
| Korea, Republic of | 11 | 3.64 | 0.95 |
| Kuwait | 1 | 3.04 | - |
| Latvia | 3 | 3.48 | 0.26 |
| Lebanon | 2 | 3.04 | 0.96 |
| Lithuania | 4 | 3.74 | 0.61 |
| Luxembourg | 2 | 3.73 | 0.47 |
| Malawi | 1 | 3.78 | - |
| Malaysia | 10 | 3.57 | 0.73 |
| Malta | 2 | 3.15 | 0.02 |
| Mauritius | 2 | 3.88 | 0.17 |
| Mexico | 68 | 3.55 | 0.75 |
| Morocco | 1 | 4 | - |
| Myanmar | 4 | 3.82 | 0.86 |
| Netherlands | 178 | 3.3 | 0.59 |
| New Zealand | 140 | 3.41 | 0.69 |
| Nicaragua | 1 | 3.38 | - |
| Nigeria | 4 | 4.15 | 0.69 |
| Norfolk Island | 1 | 3.45 | - |
| Norway | 30 | 3.15 | 0.6 |
| Oman | 1 | 4.83 | - |
| Pakistan | 3 | 3.92 | 1.02 |
| Panama | 3 | 3.11 | 1.01 |
| Papua New Guinea | 1 | 2.96 | - |
| Peru | 1 | 3.8 | - |
| Philippines | 32 | 3.48 | 0.84 |
| Poland | 23 | 3.35 | 0.56 |
| Portugal | 75 | 3.39 | 0.67 |
| Puerto Rico | 5 | 3.34 | 0.81 |
| Qatar | 2 | 3.65 | 0.78 |
| Romania | 24 | 3.58 | 0.68 |
| Russian Federation | 46 | 3.59 | 0.74 |
| Saudi Arabia | 5 | 3.53 | 0.64 |
| Serbia | 2 | 3.31 | 0.15 |
| Singapore | 39 | 3.27 | 0.78 |
| Slovakia | 2 | 3.5 | 0.24 |
| Slovenia | 12 | 3.16 | 0.56 |
| South Africa | 132 | 3.47 | 0.58 |
| Spain | 44 | 3.53 | 0.66 |
| Sri Lanka | 2 | 3.31 | 0.06 |
| Sweden | 147 | 3.35 | 0.63 |
| Switzerland | 30 | 3.43 | 0.67 |
| Taiwan, Province of China | 16 | 3.5 | 0.71 |
| Thailand | 10 | 3.55 | 0.72 |
| Trinidad and Tobago | 3 | 3.46 | 0.33 |
| Turkey | 18 | 3.43 | 0.55 |
| Ukraine | 14 | 3.53 | 0.5 |
| United Arab Emirates | 19 | 3.75 | 0.71 |
| United Kingdom | 1099 | 3.28 | 0.66 |
| United States | 5413 | 3.43 | 0.63 |
| Vietnam | 28 | 3.93 | 0.7 |
| Virgin Islands, U.S. | 1 | 3.92 | - |
| Zambia | 2 | 4 | 0 |
| Zimbabwe | 1 | 3 | - |

**Table S5.** Continuous variable descriptive statistics for the Two Factor Equanimity Scale – Even Minded State of Mind Subscale and the engineered equanimity score (A) (5 session rolling mood standard deviation) sub-sample and the Two Factor Equanimity Scale – Even Minded State of Mind Subscale and the engineered resilience score (B) (n sessions to recovery) sub-sample.

|  | **A) Equanimity score sub-sample** | | **B) Resilience score sub-sample** | |
| --- | --- | --- | --- | --- |
|  | *N* | *Mean (sd)* | *N* | *Mean (sd)* |
| **Age** | 497 | 38.45 (12.66) | 277 | 39.66 (12.63) |
| **Baseline mood** | 497 | 3.42 (0.8) | 277 | 3.43 (0.86) |
| **Practice period** | 497 | 4.72 (3.59) | 277 | 5.26 (3.48) |
| **Days between practice periods** | 497 | 14.19 (20.47) | 277 | 3.48 (1.76) |
| **Mood check-in** | 497 | 3.55 (0.64) | 277 | 2.62 (0.62) |
| **Equanimity score (rolling mood SD)** | 497 | 3.99 (0.48) | 243 | 3.64 (0.42) |
| **N sessions to recovery (resilience)** | 206 | 1.56 (0.33) | 277 | 1.42 (0.38) |
| **Meditation sessions** | 497 | 67.98 (69.58) | 277 | 84.33 (62.48) |
| **Session length** | 497 | 12.27 (4.01) | 277 | 12.91 (5.3) |
| **Days per-week** | 497 | 2.11 (0.62) | 277 | 1.99 (0.81) |
| **Days since last meditation** | 497 | 2.13 (1.06) | 277 | 1.7 (1.09) |
| **Rating score** | 497 | 4.73 (0.23) | 277 | 4.75 (0.1) |
| **Rating count** | 497 | 16974.35 (19386.25) | 277 | 14680.81 (19102.5) |
| **Play count** | 497 | 419373.48 (533144.06) | 277 | 381325.63 (530783.27) |
| **Number of practice types completed** | 497 | 6.89 (2.79) | 277 | 6.97 (2.79) |
| **Non-body to body sessions ratio** | 365 | 3.57 (5.9) | 28 | 0.49 (0.93) |
| **Two Factor Equanimity Scale - (E-MSM sub-scale)** |  |  |  |  |
| *Q1 - Whatever happens I remain serene.* | 497 | 2.8 (0.86) | 277 | 2.79 (0.86) |
| *Q2 - I am not easily disturbed by something unexpected.* | 497 | 2.72 (0.93) | 277 | 2.71 (0.9) |
| *Q3 ­- I can hardly tolerate uncomfortable emotions.* | 497 | 2.79 (0.97) | 277 | 2.79 (0.96) |
| *Q4 - I can easily get carried away by an annoyance.* | 497 | 3.08 (0.93) | 277 | 3.05 (0.91) |
| *Q5 - I feel that I am a calm person, even in moments of stress and tension.* | 497 | 3.15 (0.95) | 277 | 3.16 (0.88) |
| *Q6 - Stressful situations emotionally disturb me.* | 497 | 3.34 (0.88) | 277 | 3.39 (0.87) |
| *Q7 - It’s hard for me to be serene during the difficult moments of everyday life.* | 497 | 3.1 (0.93) | 277 | 3.15 (0.91) |
| *Q8 - I feel that the problems in my life are temporary and that they have solutions.* | 497 | 3.83 (0.92) | 277 | 3.84 (0.89) |
| ***EQUA (E-MSM) total*** | 497 | 24.19 (5.33) | 277 | 24.13 (5.07) |

**Table S6.** Categorical variable descriptive statistics for the Two Factor Equanimity Scale – Even Minded State of Mind Subscale and the engineered equanimity score (A) (5 session rolling mood standard deviation) sub-sample and the Two Factor Equanimity Scale – Even Minded State of Mind Subscale and the engineered resilience score (B) (n sessions to recovery) sub-sample.

|  | **Equanimity score sub-sample** | | **Resilience score sub-sample** | |
| --- | --- | --- | --- | --- |
|  | *N %* | *Mood mean (sd)* | *N %* | *Mood mean (sd)* |
| **Continent** |  |  |  |  |
| *Africa* | 11 (1.25) | 3.54 (0.57) | 4 (1.44) | 2.52 (0.81) |
| *Asia* | 27 (3.06) | 3.34 (0.66) | 6 (2.17) | 2.67 (0.61) |
| *Australia* | 56 (6.35) | 3.35 (0.7) | 17 (6.14) | 2.75 (0.66) |
| *Europe* | 116 (13.15) | 3.35 (0.6) | 44 (15.88) | 2.45 (0.73) |
| *North America* | 666 (75.51) | 3.43 (0.6) | 205 (74.01) | 2.64 (0.59) |
| *South America* | 6 (0.68) | 3.3 (0.86) | 1 (0.36) | 2.25 (nan) |
| **Experience** |  |  |  |  |
| *Local class (Yes)* | 308 (34.92) | 3.47 (0.6) | 101 (36.46) | 2.68 (0.61) |
| *Meditation apps (Yes)* | 657 (74.49) | 3.43 (0.61) | 213 (76.9) | 2.62 (0.64) |
| *Mentoring (Yes)* | 157 (17.8) | 3.59 (0.57) | 60 (21.66) | 2.76 (0.62) |
| *No experience (Yes)* | 121 (13.72) | 3.2 (0.57) | 26 (9.39) | 2.53 (0.57) |
| *Online course (Yes)* | 256 (29.02) | 3.47 (0.64) | 87 (31.41) | 2.75 (0.64) |
| *Retreats (Yes)* | 138 (15.65) | 3.56 (0.59) | 44 (15.88) | 2.69 (0.53) |
| **Practice type** |  |  |  |  |
| *Alternative* | 474 (53.74) | 3.48 (0.59) | 139 (50.18) | 2.66 (0.62) |
| *Body scan* | 41 (4.65) | 3.11 (0.45) | 18 (6.5) | 2.33 (0.52) |
| *Breathing meditation* | 63 (7.14) | 3.33 (0.48) | 26 (9.39) | 2.53 (0.63) |
| *Compassion meditation* | 21 (2.38) | 3.19 (0.59) | 6 (2.17) | 2.58 (0.53) |
| *Contemplation* | 20 (2.27) | 3.3 (0.78) | 13 (4.69) | 2.28 (0.52) |
| *Guided imagery* | 82 (9.3) | 3.23 (0.76) | 23 (8.3) | 2.61 (0.66) |
| *Loving-kindness (Metta)* | 4 (0.45) | 3.45 (0.67) | 1 (0.36) | 3.0 (nan) |
| *MBCT/MBSR* | 5 (0.57) | 3.58 (0.61) | 4 (1.44) | 2.66 (0.47) |
| *Mindfulness meditation* | 111 (12.59) | 3.37 (0.55) | 23 (8.3) | 2.71 (0.56) |
| *Positive affirmations* | 37 (4.2) | 3.75 (0.68) | 19 (6.86) | 2.87 (0.7) |
| *Relaxation meditation* | 9 (1.02) | 3.3 (0.93) | 2 (0.72) | 1.75 (1.06) |
| *Vipassana* | 15 (1.7) | 3.43 (0.46) | 3 (1.08) | 2.44 (0.51) |
| **Meditation worldview** |  |  |  |  |
| *Buddhism* | 76 (8.62) | 3.36 (0.56) | 24 (8.66) | 2.68 (0.63) |
| *Christianity* | 8 (0.91) | 3.37 (0.55) | 6 (2.17) | 2.52 (0.42) |
| *Hinduism* | 3 (0.34) | 3.13 (1.02) | 2 (0.72) | 2.25 (0.35) |
| *Modernism* | 201 (22.79) | 3.25 (0.55) | 65 (23.47) | 2.51 (0.56) |
| *Niches* | 107 (12.13) | 3.61 (0.73) | 35 (12.64) | 2.95 (0.69) |
| *Other* | 476 (53.97) | 3.44 (0.6) | 143 (51.62) | 2.59 (0.61) |
| Niches | 165 (18.71) | 3.51 (0.67) | 66 (23.83) | 2.82 (0.6) |
| **Meditation orientation** |  |  |  |  |
| *Positivity Based* | 279 (31.63) | 3.57 (0.53) | 75 (27.08) | 2.78 (0.55) |
| *Problem Focused* | 328 (37.19) | 3.21 (0.62) | 109 (39.35) | 2.36 (0.61) |
| *Techniques* | 110 (12.47) | 3.44 (0.53) | 27 (9.75) | 2.68 (0.6) |
| **Reason for meditating** |  |  |  |  |
| *Anxiety (Yes)* | 607 (68.82) | 3.32 (0.6) | 189 (68.23) | 2.52 (0.61) |
| *Sadness (Yes)* | 340 (38.55) | 3.25 (0.66) | 106 (38.27) | 2.44 (0.63) |
| *Stress (Yes)* | 596 (67.57) | 3.34 (0.61) | 176 (63.54) | 2.55 (0.61) |
| *Wellbeing (Yes)* | 599 (67.91) | 3.41 (0.59) | 193 (69.68) | 2.61 (0.62) |

**Table S7.** Continuous variable descriptive statistics for the adherence model sample (odds of making it to the 150^th^ session based on practice habits in the first 30 sessions) and t-statistics and p-values for differences between adherers and non-adherers.

|  | **A) Did not reach 150^th^ session** | | **B) Reached 150^th^ session** | |  | | |
| --- | --- | --- | --- | --- | --- | --- | --- |
|  | *N* | *Mean (sd)* | *N* | *Mean (sd)* | *t-statistic* | *p* | *p (fdr)** |
| **Age** | 1569 | 37.17 (12.56) | 326 | 41.84 (14.22) | -5.97 | 2.88e^-09^ | 3.17^e-08^ |
| **Baseline mood** | 1569 | 3.37 (0.73) | 326 | 3.48 (0.76) | -2.38 | 0.017 | 0.114 |
| **Practice period** | 1569 | 4.61 (3.12) | 326 | 1.95 (1.25) | 15.156 | 4.51^e-49^ | 7.22^e-48^ |
| **Days between practice periods** | 1569 | 15.6 (17.75) | 326 | 8.13 (15.01) | 7.09 | 1.92^e-12^ | 2.34^e-11^ |
| **Mood check-in** | 1569 | 3.48 (0.56) | 326 | 3.57 (0.54) | -2.75 | 0.006 | 0.048 |
| **Equanimity score (5 session rolling mood SD)** | 1523 | 4.04 (0.42) | 325 | 4.23 (0.36) | -7.66 | 2.90^e-14^ | 4.06^e-13^ |
| **N sessions to recovery (resilience)** | 745 | 1.62 (0.32) | 282 | 1.4 (0.14) | 10.91 | 2.75^e-26^ | 4.12^e-25^ |
| **Session count** | 1569 | 20.16 (6.59) | 326 | 20.39 (6.62) | -0.57 | 0.565 | 0.918 |
| **Session length** | 1569 | 11.21 (3.57) | 326 | 11.18 (3.91) | 0.14 | 0.888 | 0.918 |
| **Days per-week** | 1569 | 1.14 (0.47) | 326 | 1.36 (0.7) | -7.10 | 1.80^e-12^ | 2.34^e-11^ |
| **Days since last meditation** | 1569 | 1.44 (1.05) | 326 | 1.13 (0.5) | 5.27 | 1.55^e-07^ | 1.55^e-06^ |
| **Rating score** | 1569 | 4.72 (0.08) | 326 | 4.72 (0.08) | 0.49 | 0.626 | 0.918 |
| **Rating count** | 1569 | 20075.12 (16582.3) | 326 | 21903.68 (18791.66) | -1.77 | 0.077 | 0.294 |
| **Play count** | 1569 | 479618.58 (456033.62) | 326 | 544841.1 (548778.58) | -2.26 | 0.024 | 0.134 |
| **Number of practice types completed** | 1569 | 6.66 (1.83) | 326 | 6.45 (1.85) | 1.83 | 0.067 | 0.294 |
| **Non-body to body sessions ratio** | 1569 | 2.71 (2.67) | 326 | 3.75 (5.25) | -5.24 | 1.77^e-07^ | 1.60^e-06^ |

**Table S8.** Categorical variable descriptive statistics for the adherence model sample (odds of making it to the 150^th^ session based on practice habits in the first 30 sessions) and Chi-square and p-values for differences between adherers and non-adherers.

|  | **A) Did not reach 150^th^ session** | | **B) Reached 150^th^ session** | |  |  |  |
| --- | --- | --- | --- | --- | --- | --- | --- |
|  | *N (%)* | *Mood mean (sd)* | *N (%)* | *Mood mean (sd)* | *X^2^* | *p* | *fdr-p* |
| **Continent** |  |  |  |  |  |  |  |
| *Africa* | 27 (1.72) | 3.74 (0.58) | 7 (2.15) | 3.61 (0.37) | 0.279 | 0.597 | 1 |
| *Asia* | 47 (3.0) | 3.49 (0.61) | 13 (3.99) | 3.62 (0.62) | 0.867 | 0.352 | 1 |
| *Australia* | 167 (10.64) | 3.48 (0.56) | 31 (9.51) | 3.65 (0.69) | 0.371 | 0.542 | 1 |
| *Europe* | 297 (18.93) | 3.39 (0.56) | 65 (19.94) | 3.41 (0.55) | 0.178 | 0.673 | 1 |
| *North America* | 1023 (65.2) | 3.5 (0.55) | 209 (64.11) | 3.6 (0.49) | 0.141 | 0.707 | 1 |
| *South America* | 8 (0.51) | 3.11 (0.59) | 1 (0.31) | 5.0 (nan) | 0.236 | 0.627 | 1 |
| **Experience** |  |  |  |  |  |  |  |
| *Local Class (Yes)* | 438 (27.92) | 3.52 (0.54) | 98 (30.06) | 3.55 (0.61) | 0.613 | 0.433 | 1 |
| *Meditation Apps (Yes)* | 1188 (75.72) | 3.49 (0.56) | 250 (76.69) | 3.57 (0.54) | 0.139 | 0.709 | 1 |
| *Mentoring (Yes)* | 239 (15.23) | 3.57 (0.54) | 53 (16.26) | 3.66 (0.57) | 0.218 | 0.641 | 1 |
| *No Experience (Yes)* | 231 (14.72) | 3.38 (0.56) | 42 (12.88) | 3.49 (0.42) | 0.741 | 0.389 | 1 |
| *Online Course (Yes)* | 369 (23.52) | 3.55 (0.57) | 93 (28.53) | 3.65 (0.54) | 3.674 | 0.055 | 0.834 |
| *Retreats (Yes)* | 229 (14.6) | 3.54 (0.55) | 53 (16.26) | 3.68 (0.46) | 0.589 | 0.443 | 1 |
| **Practice type** |  |  |  |  |  |  |  |
| *Body Scan* | 67 (4.27) | 3.27 (0.55) | 15 (4.6) | 3.36 (0.6) | 0.071 | 0.789 | 1 |
| *Breathing Meditation* | 141 (8.99) | 3.56 (0.56) | 27 (8.28) | 3.48 (0.49) | 0.166 | 0.684 | 1 |
| *Compassion Meditation* | 32 (2.04) | 3.19 (0.53) | 7 (2.15) | 3.28 (0.82) | 0.016 | 0.901 | 1 |
| *Contemplation* | 41 (2.61) | 3.4 (0.7) | 5 (1.53) | 3.14 (0.39) | 1.328 | 0.249 | 1 |
| *Guided Imagery* | 183 (11.66) | 3.4 (0.54) | 47 (14.42) | 3.62 (0.6) | 1.919 | 0.166 | 1 |
| *MBCT/MBSR* | 7 (0.45) | 3.34 (0.43) | 2 (0.61) | 3.35 (0.43) | 0.159 | 0.689 | 1 |
| *Mindfulness Meditation* | 321 (20.46) | 3.46 (0.58) | 61 (18.71) | 3.55 (0.47) | 0.511 | 0.474 | 1 |
| *Other* | 659 (42.0) | 3.53 (0.54) | 140 (42.94) | 3.64 (0.53) | 0.099 | 0.754 | 1 |
| *Positive Affirmations* | 84 (5.35) | 3.63 (0.47) | 21 (6.44) | 3.63 (0.48) | 0.610 | 0.435 | 1 |
| *Relaxation Meditation* | 9 (0.57) | 3.03 (0.53) | 1 (0.31) | 2.73 (nan) | 0.366 | 0.545 | 1 |
| **Meditation worldview** |  |  |  |  |  |  |  |
| *Buddhism* | 80 (5.1) | 3.55 (0.55) | 9 (2.76) | 3.67 (0.43) | 3.296 | 0.069 | 0.885 |
| *Christianity* | 12 (0.76) | 3.37 (0.64) | 2 (0.61) | 4.12 (0.17) | 0.084 | 0.772 | 1 |
| *Modernism* | 380 (24.22) | 3.33 (0.56) | 67 (20.55) | 3.42 (0.54) | 2.014 | 0.156 | 1 |
| *Niches* | 143 (9.11) | 3.63 (0.59) | 32 (9.82) | 3.52 (0.58) | 0.159 | 0.690 | 1 |
| *Other* | 952 (60.68) | 3.51 (0.54) | 215 (65.95) | 3.62 (0.53) | 3.175 | 0.075 | 0.895 |
| **Meditation orientation** |  |  |  |  |  |  |  |
| *Niches* | 202 (12.87) | 3.6 (0.56) | 39 (11.96) | 3.57 (0.55) | 0.202 | 0.653 | 1 |
| *Positivity Based* | 662 (42.19) | 3.58 (0.49) | 161 (49.39) | 3.65 (0.54) | 5.686 | 0.017 | 0.444 |
| *Problem Focused* | 539 (34.35) | 3.28 (0.56) | 94 (28.83) | 3.39 (0.51) | 3.695 | 0.055 | 0.834 |
| *Techniques* | 166 (10.58) | 3.57 (0.65) | 32 (9.82) | 3.7 (0.5) | 0.1684 | 0.682 | 1 |
| **Reason for meditating** |  |  |  |  |  |  |  |
| *Sadness (Yes)* | 560 (35.69) | 3.35 (0.57) | 107 (32.82) | 3.43 (0.49) | 0.974 | 0.324 | 1 |
| *Stress (Yes)* | 1055 (67.24) | 3.45 (0.56) | 198 (60.74) | 3.48 (0.51) | 5.097 | 0.024 | 0.551 |
| *Wellbeing (Yes)* | 1036 (66.03) | 3.49 (0.56) | 216 (66.26) | 3.56 (0.51) | 0.006 | 0.937 | 1 |

**Table S9.** Continuous variable descriptive statistics for the binary interoceptive vs exteroceptive meditator sample.

|  | **A) Interoceptive meditator** | | **B) Exteroceptive meditator** | |  |  |  |
| --- | --- | --- | --- | --- | --- | --- | --- |
|  | *N* | *Mean (sd)* | *N* | *Mean (sd)* | *t-statistic* | *p* | *p (fdr)** |
| **Age** | 1080 | 36.8 (13.29) | 5829 | 37.39 (12.56) | -9.55 | 1.70^e-21^ | 2.04^e-20^ |
| **Baseline mood** | 1080 | 3.2 (0.77) | 5829 | 3.35 (0.72) | -4.42 | 1.01^e-05^ | 5.04^e-05^ |
| **Practice period** | 1080 | 5.01 (3.4) | 5829 | 6.12 (3.84) | -2.18 | 0.029 | 0.059 |
| **Days between practice periods** | 1080 | 34.08 (37.46) | 5829 | 29.82 (34.32) | 11.36 | 1.25^e-29^ | 1.62^e-28^ |
| **Mood check-in** | 1080 | 3.29 (0.7) | 5829 | 3.42 (0.64) | -8.16 | 3.89^e-16^ | 3.50^e-15^ |
| **Equanimity score (5 session rolling mood SD)** | 565 | 4.05 (0.5) | 3674 | 4.03 (0.46) | -8.73 | 3.65^e-18^ | 3.65^e-17^ |
| **N sessions to recovery (resilience)** | 166 | 1.56 (0.3) | 1342 | 1.55 (0.31) | 13.29 | 3.36^e-38^ | 4.71^e-37^ |
| **Session count** | 1080 | 35.42 (50.66) | 5829 | 51.56 (64.1) | -79.85 | 0 | 0 |
| **Session length** | 1080 | 11.55 (4.76) | 5829 | 11.28 (3.89) | -2.48 | 0.013 | 0.039 |
| **Days per-week** | 1080 | 1.08 (0.33) | 5829 | 1.05 (0.25) | 0.94 | 0.346 | 0.346 |
| **Days since last meditation** | 1080 | 1.7 (1.37) | 5829 | 1.63 (1.32) | 8.93 | 5.31^e-19^ | 5.84^e-18^ |
| **Rating score** | 1080 | 4.72 (0.1) | 5829 | 4.73 (0.12) | -6.03 | 1.76^e-09^ | 1.24^e-08^ |
| **Rating count** | 1080 | 18478.33 (19427.09) | 5829 | 18000.3 (18451.31) | 5.85 | 5.09^e-09^ | 3.06^e-08^ |
| **Play count** | 1080 | 506094.85 (591225.78) | 5829 | 420299.2 (475761.17) | 3.57 | 3.64^e-04^ | 0.001 |
| **Number of practice types completed** | 1080 | 4.78 (2.2) | 5829 | 6.16 (2.73) | -32.85 | 4.91^e-220^ | 7.37^e-219^ |
| **Non-body to body sessions ratio** | 1080 | 0.4 (1.91) | 5829 | 2.83 (3.59) | -6.73 | 1.82^e-11^ | 1.45^e-10^ |

**Table S10.** Categorical variable descriptive statistics for the interoceptive vs exteroceptive sample and Chi-square and p-values for differences between interoceptive and exteroceptive meditators.

|  | **A) Body meditator** | | **B) Non-body meditator** | |  |  |  |
| --- | --- | --- | --- | --- | --- | --- | --- |
|  | *N (%)* | *Mood mean (sd)* | *N (%)* | *Mood mean (sd)* | *X^2^* | *p* | *fdr-p* |
| **Continent** |  |  |  |  |  |  |  |
| *Africa* | 14 (1.3) | 3.61 (0.59) | 104 (1.78) | 3.56 (0.54) | 1.292 | 0.256 | 0.829 |
| *Asia* | 35 (3.24) | 3.43 (0.59) | 220 (3.77) | 3.49 (0.78) | 0.729 | 0.393 | 0.864 |
| *Australia* | 104 (9.63) | 3.34 (0.65) | 598 (10.26) | 3.42 (0.64) | 0.395 | 0.529 | 0.896 |
| *Europe* | 215 (19.91) | 3.19 (0.71) | 1136 (19.49) | 3.33 (0.64) | 0.102 | 0.750 | 0.938 |
| *North America* | 702 (65.0) | 3.3 (0.7) | 3669 (62.94) | 3.44 (0.62) | 1.657 | 0.198 | 0.786 |
| *South America* | 10 (0.93) | 3.25 (0.77) | 102 (1.75) | 3.58 (0.77) | 3.879 | 0.049 | 0.424 |
| **Experience** |  |  |  |  |  |  |  |
| *Local Class (Yes)* | 246 (22.78) | 3.39 (0.69) | 1687 (28.94) | 3.45 (0.61) | 17.179 | 3.40^e-05^ | 5.10^e-04^ |
| *Meditation Apps (Yes)* | 695 (64.35) | 3.3 (0.68) | 4225 (72.48) | 3.45 (0.62) | 29.382 | 5.94^e-08^ | 1.25^e-06^ |
| *Mentoring (Yes)* | 102 (9.44) | 3.33 (0.67) | 838 (14.38) | 3.53 (0.61) | 18.856 | 1.41^e-05^ | 2.33^e-04^ |
| *No Experience (Yes)* | 278 (25.74) | 3.26 (0.74) | 1027 (17.62) | 3.31 (0.67) | 39.232 | 3.76^e-10^ | 9.41^e-09^ |
| *Online Course (Yes)* | 193 (17.87) | 3.33 (0.69) | 1395 (23.93) | 3.48 (0.64) | 18.914 | 1.37^e-05^ | 2.33^e-04^ |
| *Retreats (Yes)* | 109 (10.09) | 3.32 (0.83) | 843 (14.46) | 3.51 (0.61) | 14.644 | 1.3^e-04^ | 1.81^e-03^ |
| **Practice type** |  |  |  |  |  |  |  |
| *Body Scan* | 169 (15.65) | 3.25 (0.6) | 222 (3.81) | 3.18 (0.68) | 239.230 | 5.79^e-54^ | 1.91^e-52^ |
| *Breathing Meditation* | 206 (19.07) | 3.35 (0.68) | 552 (9.47) | 3.39 (0.65) | 86.048 | 1.76^e-20^ | 4.92^e-19^ |
| *Compassion Meditation* | 26 (2.41) | 2.95 (0.78) | 135 (2.32) | 3.27 (0.66) | 0.033 | 0.855 | 0.938 |
| *Contemplation* | 12 (1.11) | 3.22 (1.02) | 183 (3.14) | 3.36 (0.65) | 13.668 | 2.18^e04^ | 2.83^e-03^ |
| *Guided Imagery* | 63 (5.83) | 3.07 (0.85) | 715 (12.27) | 3.36 (0.65) | 37.735 | 8.11^e-10^ | 1.95^e-08^ |
| *Loving-Kindness (Metta)* | 6 (0.56) | 2.33 (0.98) | 52 (0.89) | 3.17 (0.65) | 1.239 | 0.266 | 0.829 |
| *MBCT/MBSR* | 22 (2.04) | 3.21 (0.61) | 35 (0.6) | 2.95 (0.74) | 22.983 | 1.63^e-06^ | 3.11^e-05^ |
| *Mindfulness Meditation* | 382 (35.37) | 3.37 (0.59) | 901 (15.46) | 3.39 (0.65) | 238.939 | 6.70^e-54^ | 2.14^e-52^ |
| *Other* | 107 (9.91) | 3.23 (0.94) | 2578 (44.23) | 3.51 (0.59) | 451.703 | 3.07^e-100^ | 1.11^e-98^ |
| *Positive Affirmations* | 15 (1.39) | 3.57 (1.02) | 305 (5.23) | 3.55 (0.61) | 30.474 | 3.38^e-08^ | 7.44e^e-07^ |
| *Relaxation Meditation* | 39 (3.61) | 3.21 (0.65) | 87 (1.49) | 3.1 (0.82) | 22.842 | 1.76^e-06^ | 3.17^e-05^ |
| *Vipassana* | 33 (3.06) | 3.34 (0.68) | 64 (1.1) | 3.27 (0.75) | 25.225 | 5.10^e-07^ | 1.02^e-05^ |
| **Meditation worldview** |  |  |  |  |  |  |  |
| *Buddhism* | 163 (15.09) | 3.4 (0.67) | 353 (6.06) | 3.3 (0.68) | 107.670 | 3.17^e-25^ | 9.52^e-24^ |
| *Christianity* | 4 (0.37) | 3.25 (1.71) | 53 (0.91) | 3.14 (0.78) | 3.233 | 0.072 | 0.527 |
| *Modernism* | 515 (47.69) | 3.23 (0.64) | 1239 (21.26) | 3.32 (0.67) | 336.008 | 4.72^e-75^ | 1.65^e-73^ |
| *Niches* | 59 (5.46) | 3.35 (0.89) | 673 (11.55) | 3.53 (0.65) | 35.591 | 2.43^e-09^ | 5.60^e-08^ |
| *Other* | 338 (31.3) | 3.33 (0.74) | 3501 (60.06) | 3.46 (0.61) | 305.365 | 2.23E-68 | 7.59^e-67^ |
| **Meditation orientation** |  |  |  |  |  |  |  |
| *Niches* | 95 (8.8) | 3.34 (0.9) | 990 (16.98) | 3.5 (0.65) | 46.144 | 1.10^e-11^ | 2.97^e-10^ |
| *Positivity Based* | 258 (23.89) | 3.42 (0.7) | 2305 (39.54) | 3.57 (0.56) | 95.695 | 1.34^e-22^ | 3.89^e-21^ |
| *Problem Focused* | 465 (43.06) | 3.16 (0.67) | 1924 (33.01) | 3.21 (0.66) | 40.668 | 1.80^e-10^ | 4.69^e-09^ |
| *Techniques* | 262 (24.26) | 3.39 (0.62) | 610 (10.46) | 3.43 (0.66) | 157.216 | 4.59^e-36^ | 1.42^e-34^ |
| **Reason for meditating** |  |  |  |  |  |  |  |
| *Sadness (Yes)* | 380 (35.19) | 3.15 (0.72) | 2205 (37.83) | 3.29 (0.63) | 2.718 | 0.099 | 0.609 |
| *Stress (Yes)* | 740 (68.52) | 3.26 (0.7) | 3869 (66.38) | 3.38 (0.63) | 1.885 | 0.169 | 0.774 |
| *Wellbeing (Yes)* | 655 (60.65) | 3.31 (0.71) | 3809 (65.35) | 3.43 (0.62) | 8.794 | 0.003 | 0.036 |

**Figure S1.** Insight Timers in-app mood check-in screen.

**
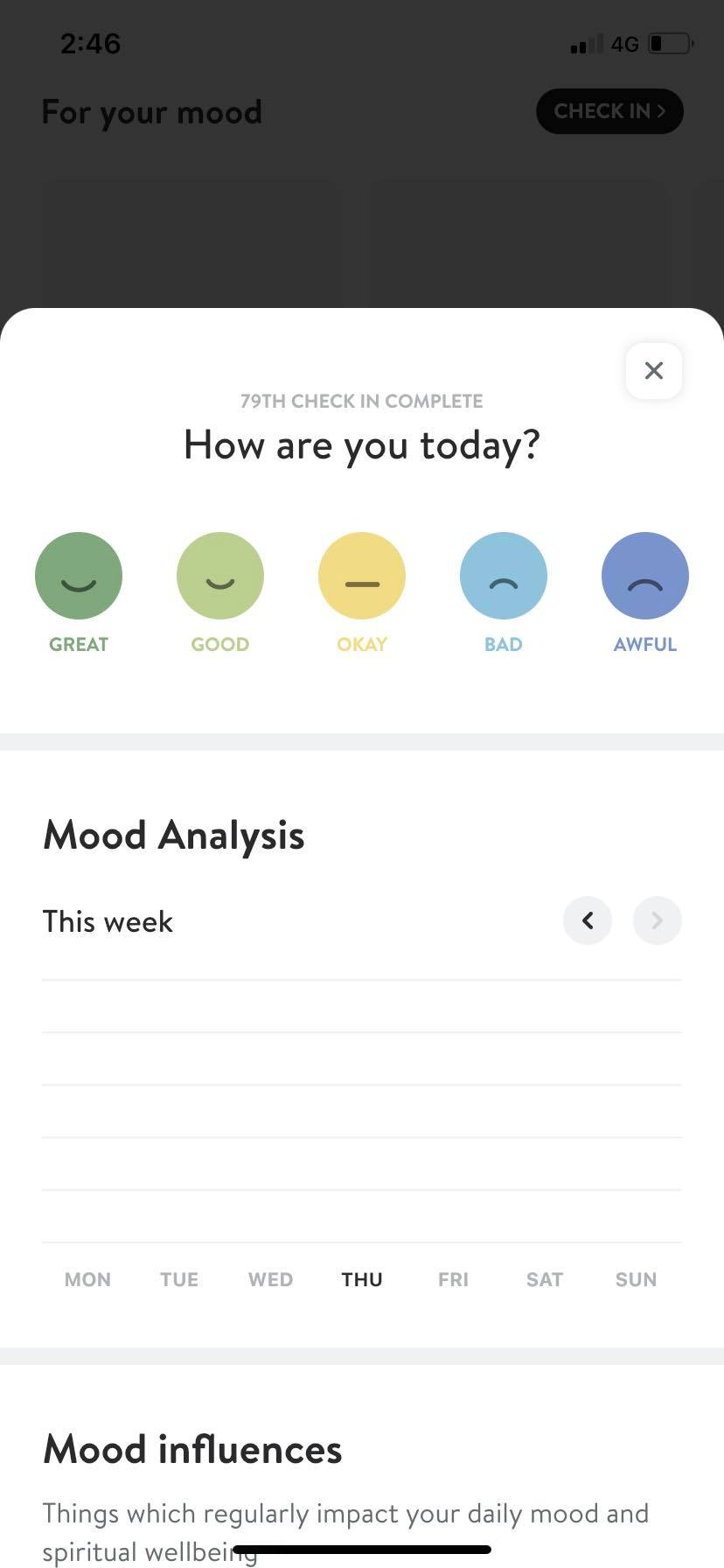
**

**Figure S2.** User onboarding was composed of a sign-up flow that asked the following questions. Answers to these questions were used as covariates in all analyses.

**1. 2. 3.**


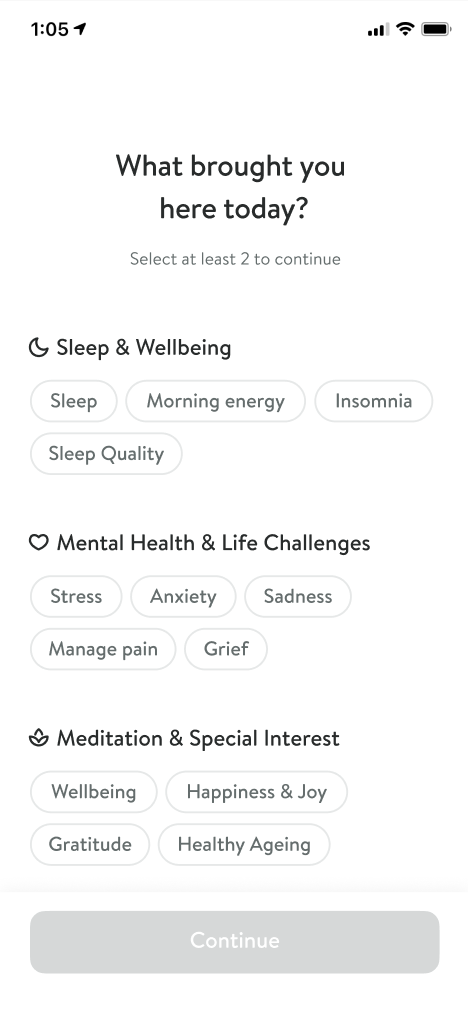

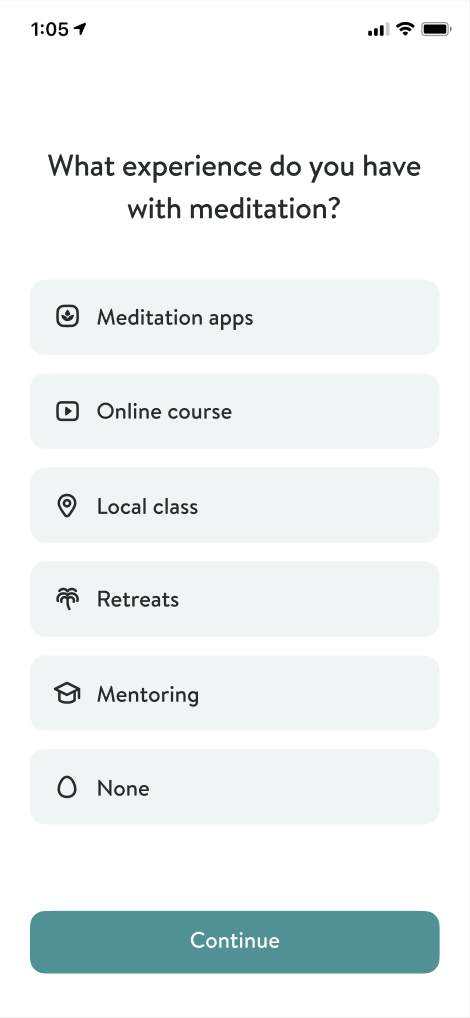

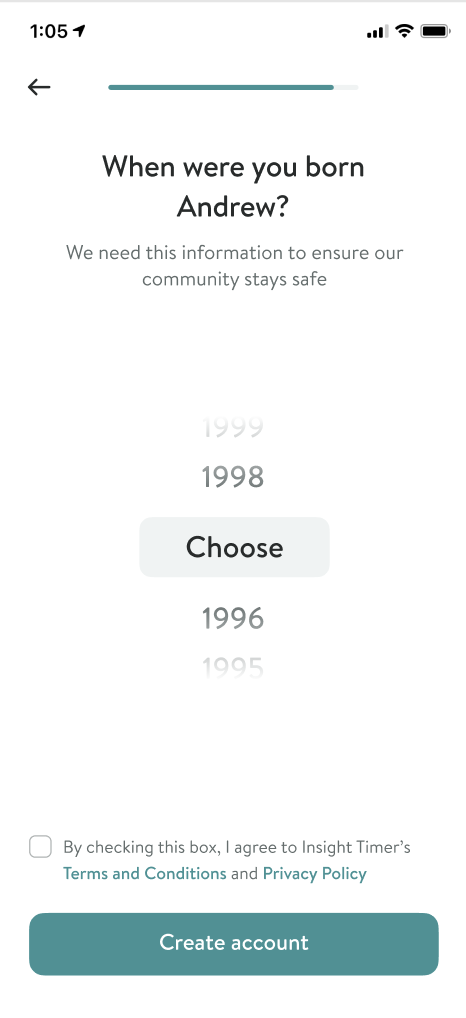


**Figure S3.** Schema for practice periods and shifted mood check-ins.

**
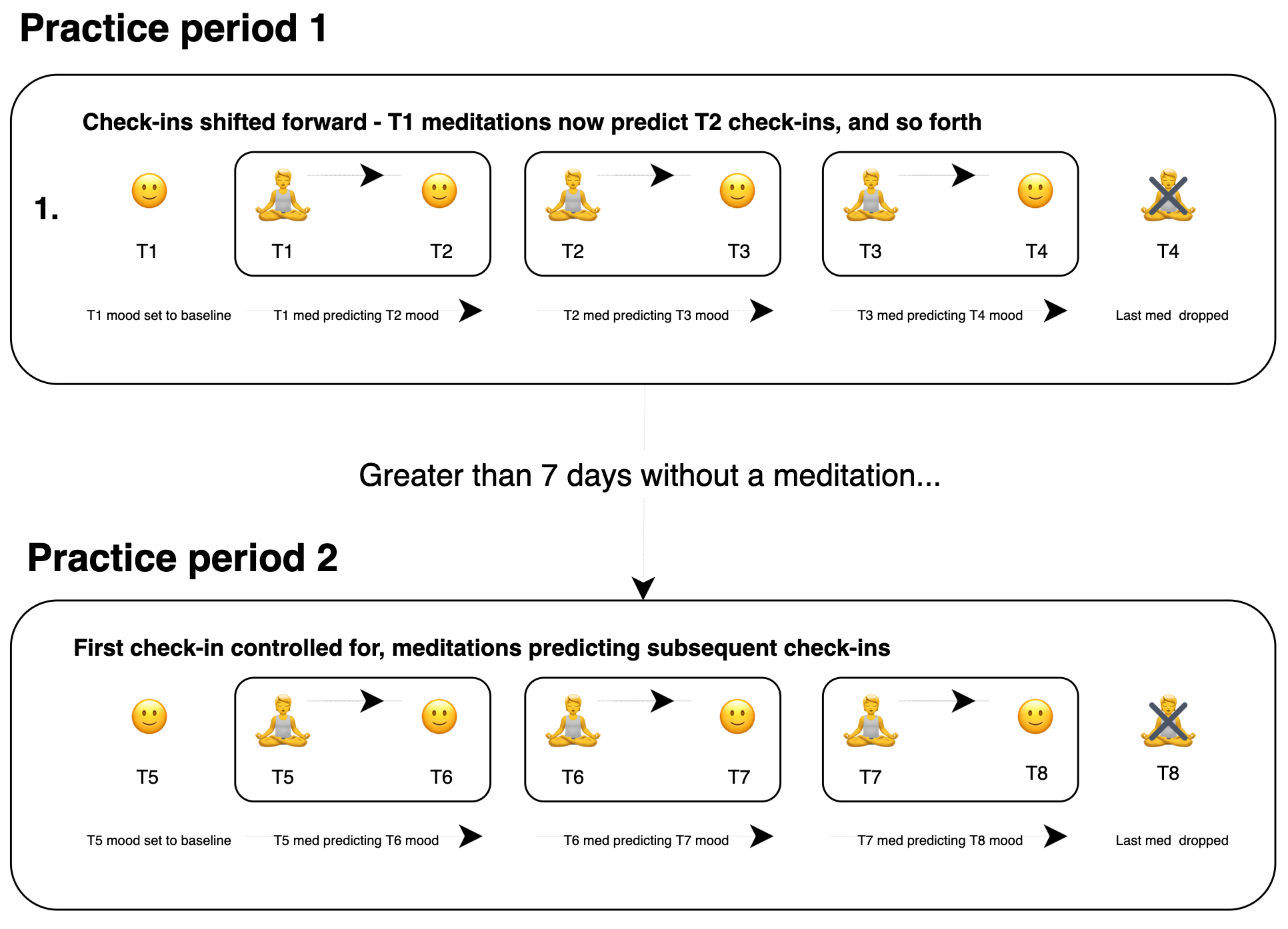
**

**Figure S4.** Distribution of the number of days since a user’s last meditation session.

**
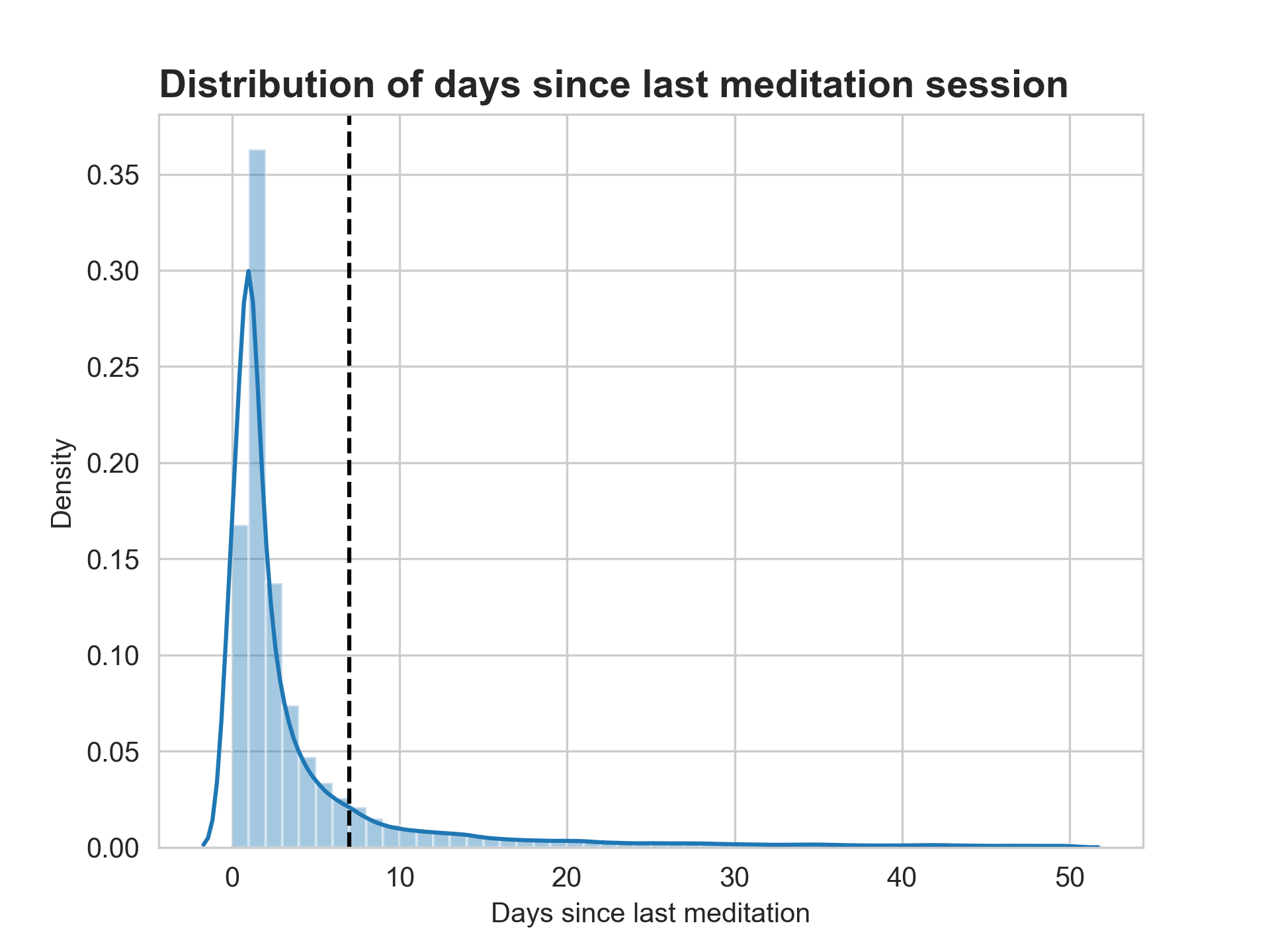
**

**Figure S5.** Distribution of mood check-in counts by user and practice period. Black dashed line = the median number of mood check-ins within a user’s practice period. This value equates to 5.


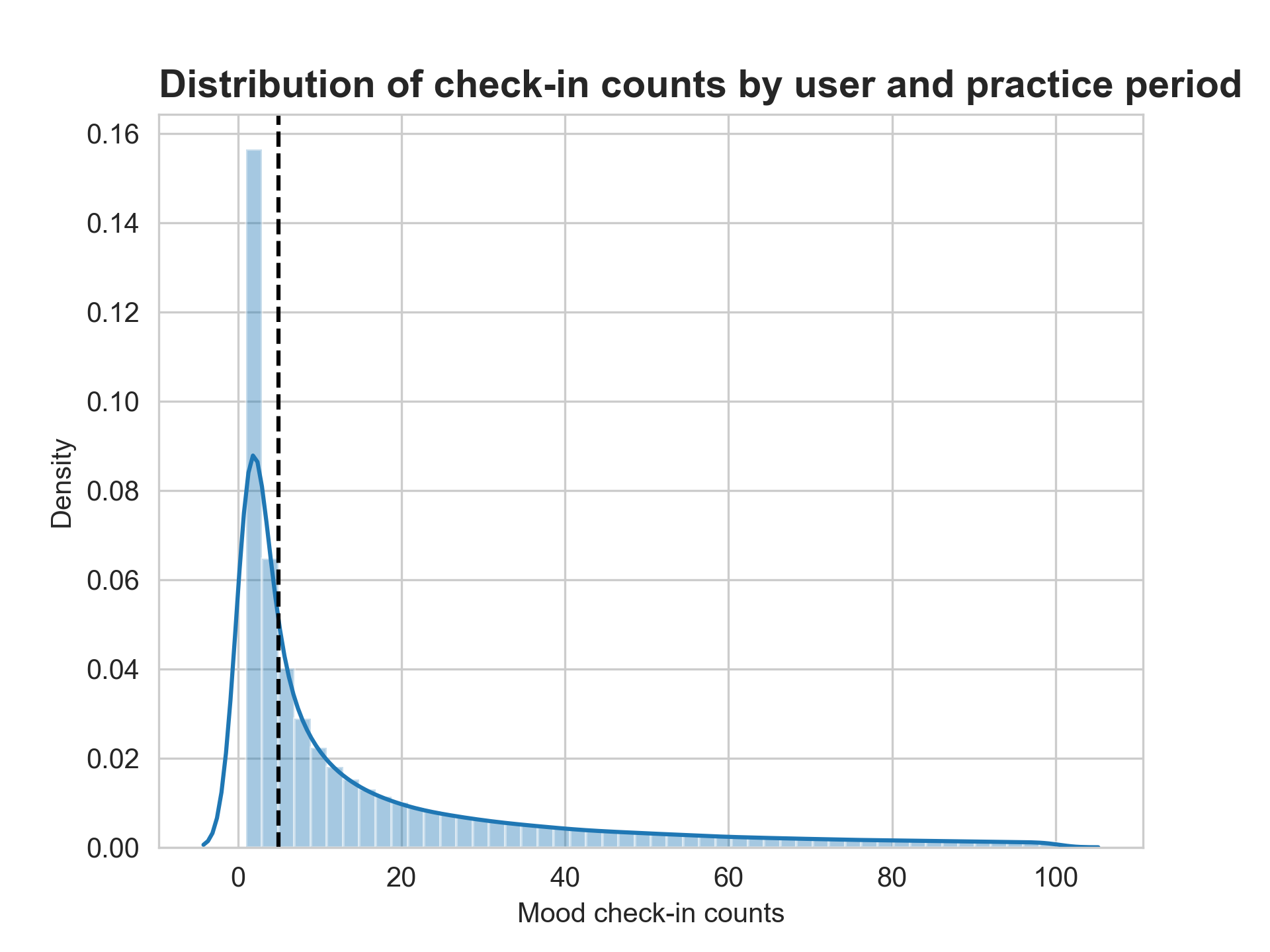


**Figure S6.** Average number of mood check-ins per-week. For the resilience measure, a user’s most recent mood check-in was compared to their prior week’s average check-in.

**
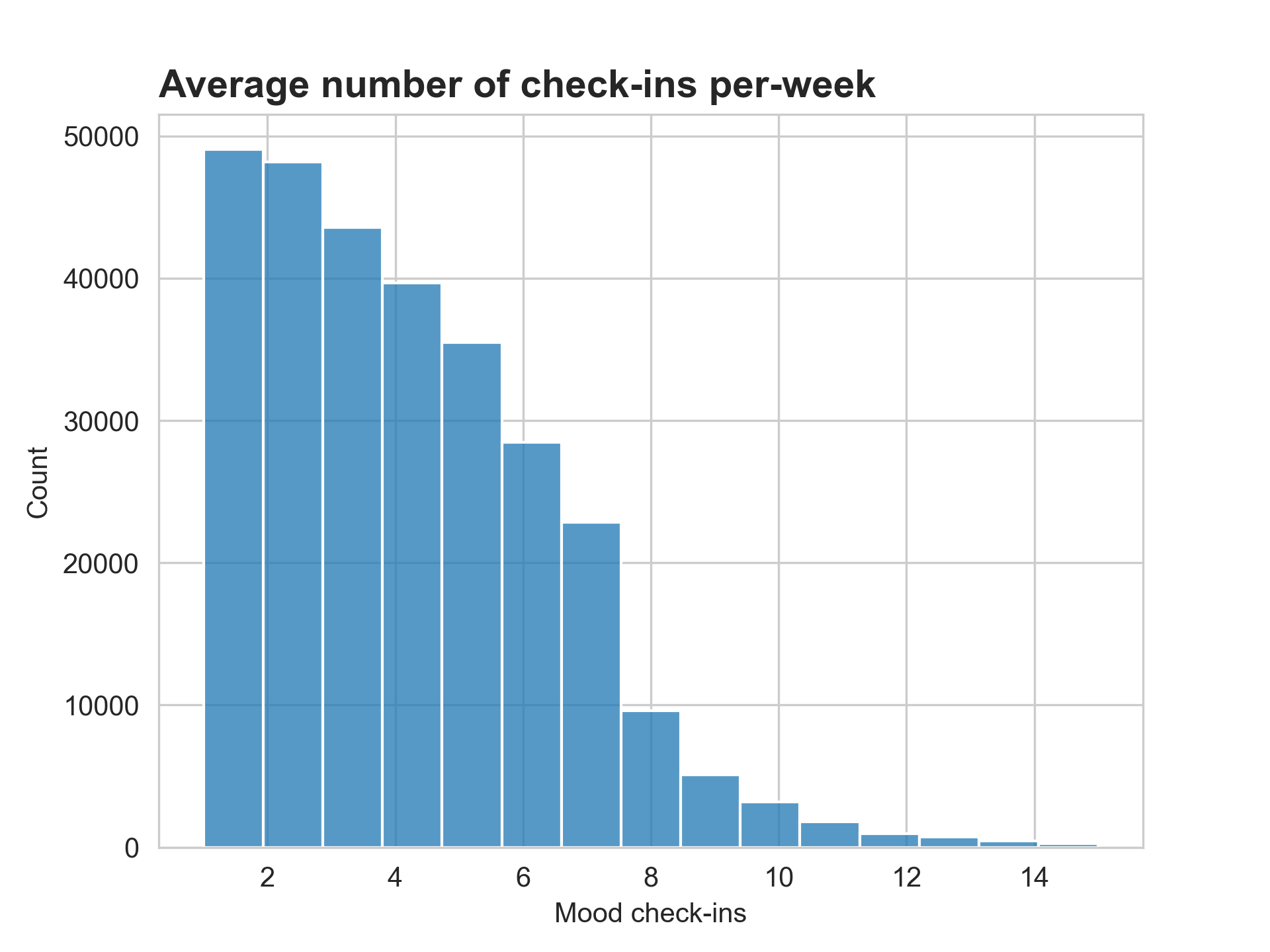
**

**Figure S7.** Cumulative number of meditation sessions distribution. The black dashed line on the left corresponds to the median value (30 sessions) while the black dashed line on the right corresponds to the top deciles value (150 sessions). Meditators practice habits from the first 30 sessions were used to predict the odds of reaching the 150^th^ session.

**
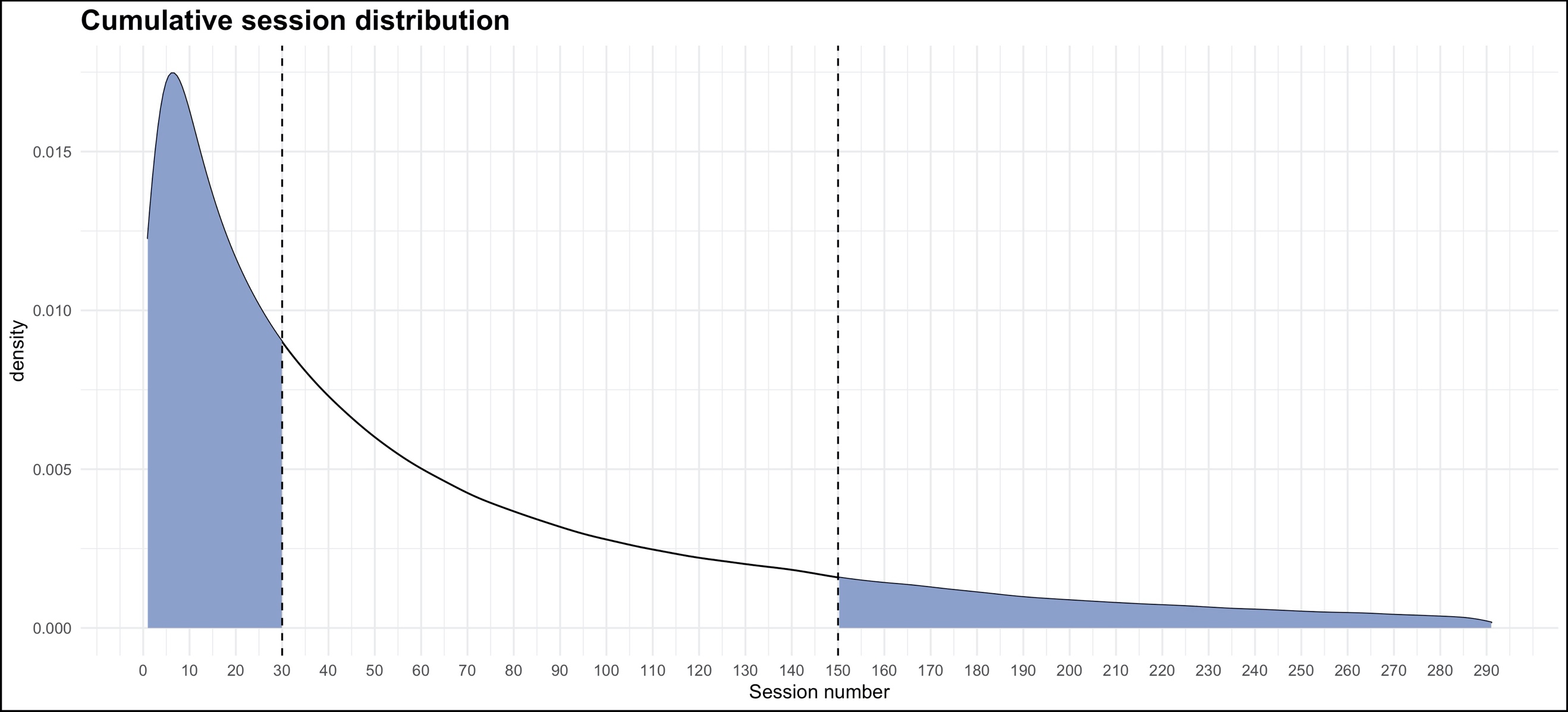
**

**Figure S8.** Counts and categorisation of body (interoceptive meditations) and non-body (exteroceptive meditations).

**
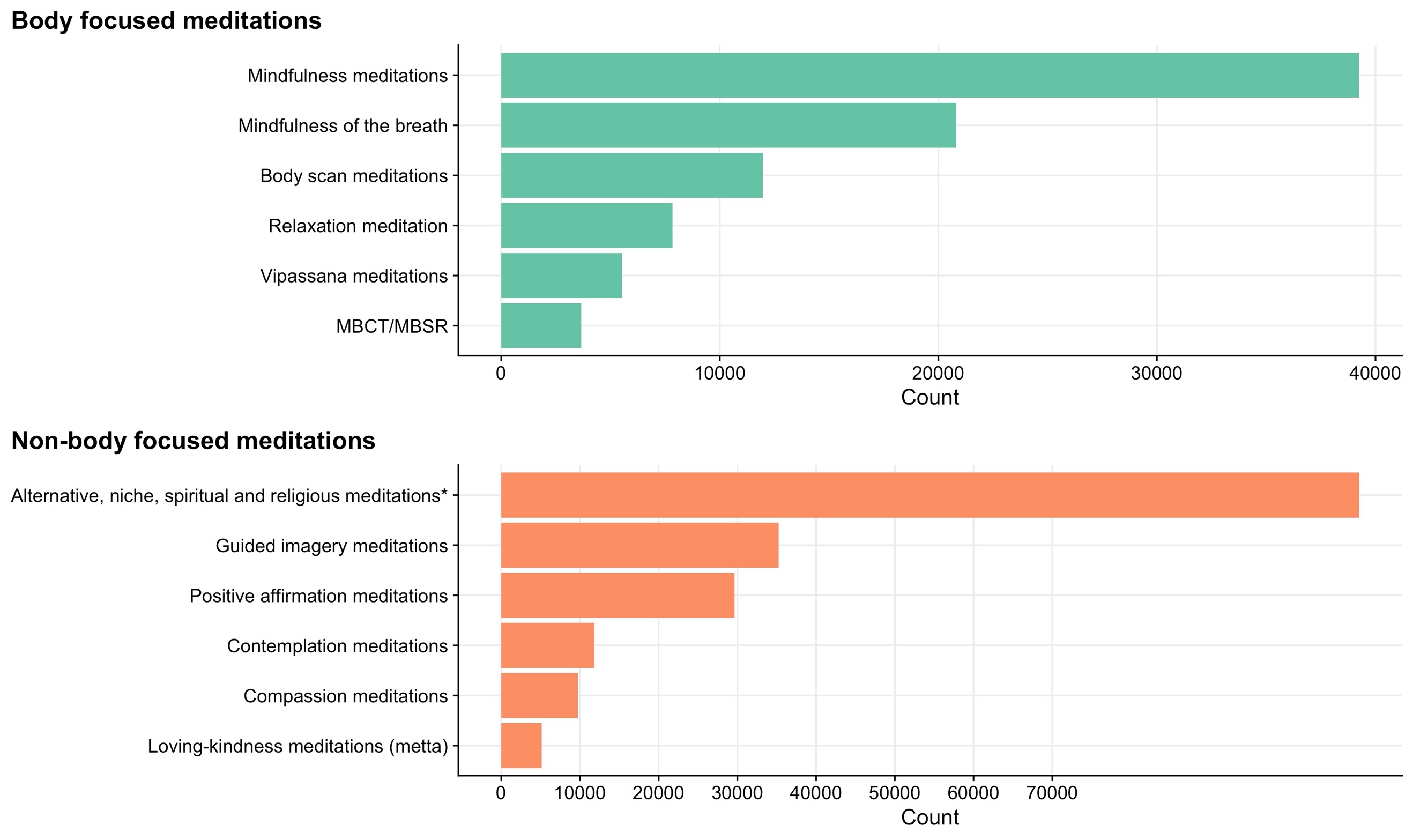
**

**Figure S9.** Session time point distribution pre and post trimming with the boxplot method (A and B) and after harmonisation to nearest values of 5 (C).


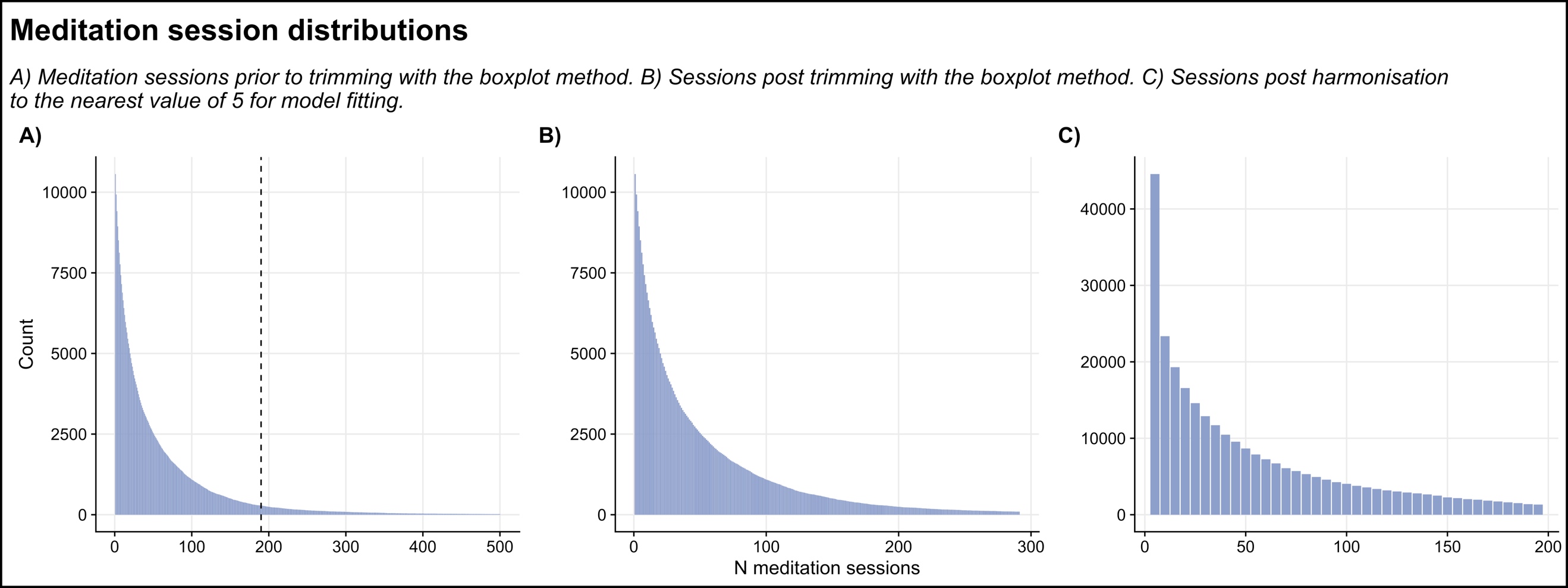


**Figure S10.** Session length distribution pre and post trimming with the boxplot method (A and B) and after harmonisation to nearest values of 5 (C).


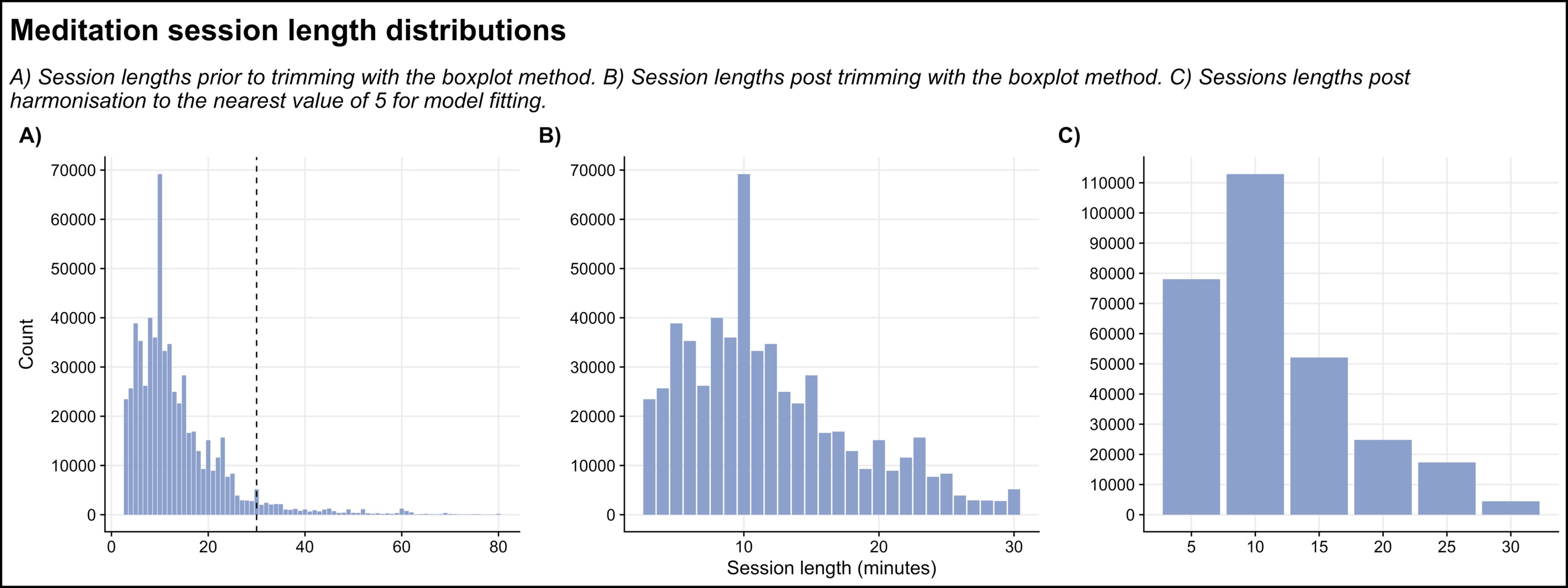


**Figure S11.** Relative frequency plots for each session time point and practice habit level combinations from Tables S1 to S3.


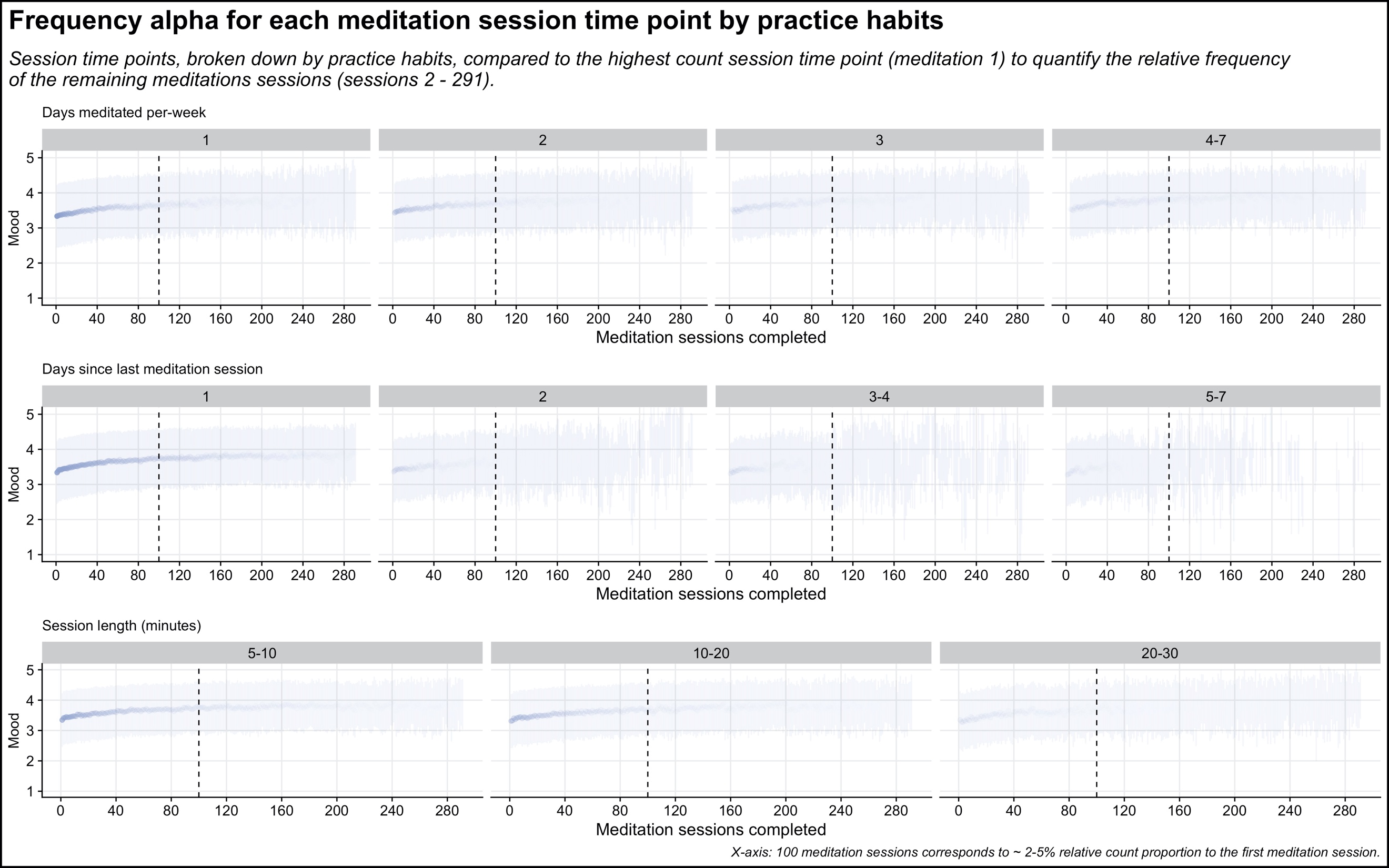

Supplement: Multimedia Appendix 1 [file jmir_v25i1e43358_app1.docx]
